# B chromosome contains active genes and impacts the transcription of A chromosomes in maize (*Zea mays L.*)

**DOI:** 10.1186/s12870-016-0775-7

**Published:** 2016-04-16

**Authors:** Wei Huang, Yan Du, Xin Zhao, Weiwei Jin

**Affiliations:** National Maize Improvement Center of China, Beijing Key Laboratory of Crop Genetic Improvement, China Agricultural University, Beijing, 100193 China

**Keywords:** Maize, B chromosome, RNA-seq, Transcription, Evolution

## Abstract

**Background:**

The dispensable maize (*Zea mays L.*) B chromosome is highly heterochromatic and widely believed to be devoid of functional genes. Although low-copy B chromosome causes no obvious phenotype variation, its existence might influence A genome gene expression. Previous studies suggested that B chromosomes are evolved from standard chromosomes; therefore, they might contain genic regions showing homology with A chromosome sequences.

**Results:**

Our data suggested that maize B chromosome influences the A-genome transcription with stronger effect associated with an increase in copy number of B chromosome. In total 130 differently expressed genes were detected in comparison between with and without B chromosome lines. These differentially expressed genes are mainly involved in cell metabolism and nucleotide binding. Using Starter + B, we amplified ten B chromosome loci with high sequence similarity to A-genome genes. Fluorescence *in situ* hybridization (FISH) confirmed that at least four ~5 kb-sized genes are located on the B chromosome. In addition, through *de novo* assembly of the reads not unmapped to maize B73 reference genome together with PCR validation, we found three B-located LTR; in particular, one of them, the 3.2 kb comp75688, is expressed in a B-dosage dependent manner.

**Conclusion:**

We found that in the presence of maize B chromosome, the transcription of A genome genes was altered, with more impact by the increase of the B chromosome number. The B-located transcriptionally active genes showed high similarity to their A-genome homologues, and retrotransposons on B chromosome also have partial homologous to A genome sequences. Our data shed more lights on the genome structure and evolution of the maize B chromosome.

**Electronic supplementary material:**

The online version of this article (doi:10.1186/s12870-016-0775-7) contains supplementary material, which is available to authorized users.

## Background

The B chromosomes are supernumerary ones not necessary for the normal growth and development of an organism. They have been documented in a wide range of species from fungi to higher eukaryotes, including plants and animals [1]. B chromosomes are called selfish chromosomes because their existence does not confer any obvious advantages to the hosts, and they do not pair or recombine with the A chromosomes and accumulate through a non-Mendelian manner [2]. The B chromosome of maize has been studied for several decades. It is a highly heterochromatic chromosome with a centric heterochromatin and four heterochromatic blocks in the long arm [3–5], along with a proximal and a distal euchromatic region [6]. This supernumerary chromosome is only present in a few maize varieties, which indicates that the maintenance of the B chromosome is not through the fitness selection, but through an accumulation mechanism which is nondisjunction at the second pollen mitosis and preferential fertilization of the egg by the sperm containing the B chromosomes [7, 8]. Several regions control the nondisjunction feature, including the distal euchromatic tip [6], a site in the proximal euchromatin [9], its centromere and centric knob [10, 11].

The origin of Bs is assumed to be derived from standard A chromosomes of either the same or related species [12]. Studies on *Canidae* identified several chromosome regions of domestic dog that show co-hybridization to wild canid B chromosomes [13]. Recently, the utilization of next generation sequencing revealed that the B chromosomes of fish species *Astatolilapia latifasciata* and *Astyanax paranae* [14, 15] were originated from multiple As. Sequencing of rye B chromosome showed that the B chromosome was originated from chromosomes 3R and 7R; it then accumulated large amounts of specific repetitive elements and insertions of organellar sequences during the independent evolution process [16]. Similar results have been obtained in maize. Researchers found that the B specific repeats ZmBs is homologous to Cent4 (centromere specific repeats of chromosome 4) [17, 18], raising the possibility that the centromere of chromosome 4 might be the donor of B chromosome centromere. Cheng and Lin microdissected B chromosome and cloned 19 B chromosome sequences, with only one being the B-specific CL-repeat and the remainder being present on both A and B chromosomes [19]. Recently, by using the Random Amplified Polymorphic DNA (RAPD) technology, four short repetitive sequences were found to locate on both A and B chromosomes [20]. However, it is still difficult to reveal the origin of B chromosome specific repetitive sequences.

It is widely believed that B chromosomes are highly heterochromatic and not essential, as they do not carry any genes that are indispensable for plant development [2, 21]. However, the B chromosome is not genetically inert. The presence of maize B chromosomes alters the recombination frequency of A chromosomes [22], causes leaf stripping [23] and reduces fertility and vigor when present in multiple copies [24]. More evidence supports the transcriptional nature of B chromosomes. The B-derived rRNA transcripts were found in the grasshopper *Eyprepocnemis plorans* [25, 26] and plant *Crepis capillaris* [27]. Some genes on B chromosome of cichlid *A. latifasciata* were largely intact but the expression of three cell cycle related genes was confirmed [15]. Protein coding genes on the B chromosome were also found in the fungus *Nectria haematococca* [28] and the Siberian roe deer *Capreolus pygargus* [29]. In rye, parts of pseudogene-like fragments on Bs were transcribed, and the presence of B chromosome affected the transcription of A-genome genes [30]. In maize, the portion of StarkB, a large DNA repeat element which is composed of fragments homologous to A genome and B-specific sequences, was confirmed to have transcriptional activity with Northern Blotting and RT-PCR [31]. Two B chromosome-located RAPD fragments, which are homologous to retrotransposon Grande1 and GrandeB, were also transcribed [20]. In another study, researchers determined four B-related short transcripts (~200 bp on average) via the cDNA-AFLP (cDNA-amplified fragment length polymorphism) method [32], and two of which showed B-specific transcription and the other two were transcribed in tissues with or without B chromosome. Current evidence suggests that the maize B chromosome is transcriptionally active and that the presence of B chromosome might negatively affect A-genome gene expression [32]. However,due to the limitation of cDNA-AFLP method, they failed to provide the details regarding the genome-wide impact of B chromosome on A-genome gene transcription, especially the expression level variation of genes which are expressed in lines with or without B chromosome, let alone the function of differentially expressed genes. In addition, it is still not clear whether the short transcripts are part of protein-coding genes. Moreover, up to now, few discernible genes have been revealed on the B chromosomes in maize.

In this study, we applied RNA-seq to analyze the transcriptome of maize with varying copies of B chromosome (B73 + 0B, B73 + 1B and B73 + 6Bs). We found that the expression of A-genome genes is indeed influenced in the presence of B chromosomes, with more B chromosomes having greater effect. Using oat-maize-addition line containing maize B chromosome we amplified four upregulated genes, each ~5 kb in length. These four genes acquired multiple SNPs or insertions/deletions compared to their A-homologies, and their location on B chromosome was confirmed by FISH assay. Since there is no reference genome sequence of B chromosome, we used *de novo* assembly of unmapped (to maize B73 reference genome) reads to identify putative B-derived transcripts, and then verified their origin through maize and oat-maize-addition line with or without- B chromosome. We successfully identified three B-located LTR sequences, a 484 bp comp30393 whose A chromosome homology has several SNPs, the 1,633 bp comp74447 and 3.2 kb comp75688 that are B chromosome specific sequences. Especially, the comp75688 showed B-dosage dependent expression, the expression level in B73 + 6Bs is about six-fold of that in B73 + 1B tissue. Our results shed more lights on the genome structure and evolution of the maize B chromosome.

## Results

### Generation of RNA-seq libraries

**Fig. 1:**
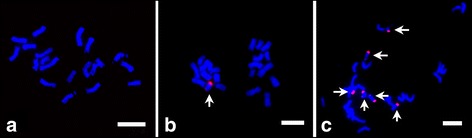
FISH identification of B chromosome number. The red signal is digoxingenin-labeled ZmBs. The B chromosome number was confirmed by counting ZmBs signals and total chromosome number. (**a**) B73 + 0B, (**b**) B73 + 1B, (**c**) B73 + 6Bs. Arrows indicate the B chromosome. Bar = 5 μm

To investigate the transcription of maize B chromosome and the effect of B chromosome on maize A-genome expression, we generated RNA-seq libraries of 14-day young leaves from maize seedlings carrying varying number of B chromosome. A B73 inbred line containing B chromosome was self-pollinated, and the number of B chromosome in offspring varied because of the unique transmission of B chromosomes. We used fluorescence *in situ* hybridization (FISH) with maize B-specific repeats ZmBs as probe to determine the number of maize B chromosomes. As shown in Fig. 1a, the cell of B73 + 0B contains 20 chromosomes (2n = 2x = 20), the B73 + 1B has 21 chromosomes, with one B chromosome (2n = 2x = 20 + 1B, Fig. 1b), and six B chromosomes presented in the B73 + 6Bs plants (Fig. 1c, 2n = 2x = 20 + 6B).

Then, we screened two batches of seedlings. For each batch, nine with-/without-B seedlings cultured in the light chamber under the same growth condition (28 °C 16 h day, 20 °C 8 h night) were chosen for RNA extraction. The 101 bp pair-end RNA-seq libraries were constructed with mRNA of 14-day young leaves from each plant. The first batch has four RNA-seq libraries, one for B73 + 6Bs (6B_1), one for B73 + 1B (1B_1), and two without B chromosome (0B_1 and 0B_2); and the seocnd batch contains one 0B (0B_3), two B73 + 1B (1B_2 and 1B_3) and two B73 + 6Bs (6B_2 and 6B_3). In sum, we got three groups of data with 0, 1 and 6 B chromosome(s), and three samples for each group.

### The expression of A-genome genes was affected by the presence of B chromosome

The nine RNA-seq libraries had between 36,491,110 and 97,545,922 reads, with at least 28,279,554 reads aligned uniquely to the reference sequence (Additional file 1: Table S1). Both spearman and pearson correlation indicates that the three libraries in each group showed a great correlation (Additional file 2: Table S2). The weakest correlation is between 0B_2 from batch1 and 0B_3 from the batch2 (>0.91), and the three replicates in the B73 + 6Bs group have the highest correlation (>0.95).

**Fig. 2:**
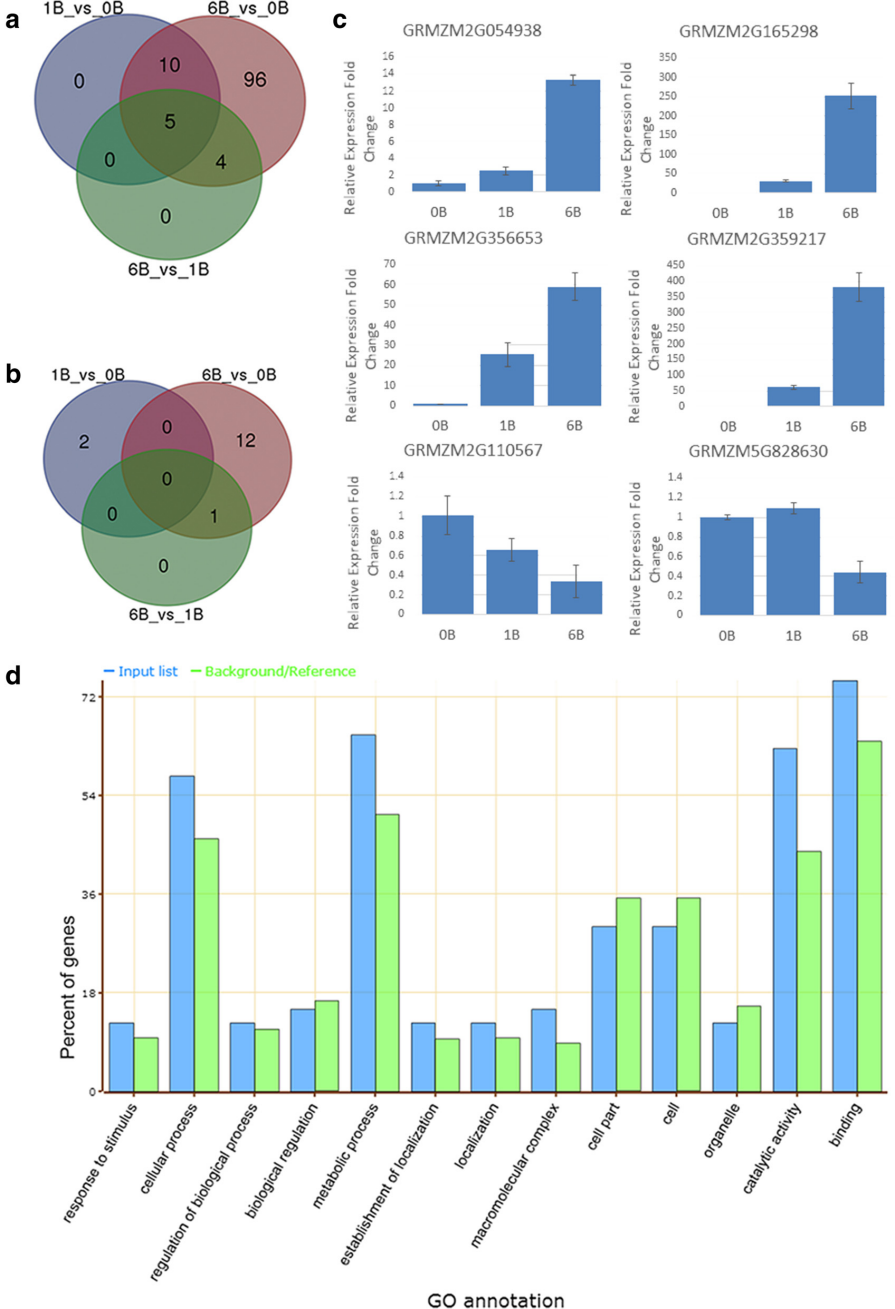
Differential gene expression in the presence/absence of B chromosome. (**a**) Up-regulated genes in both groups. (**b**) Down-regulated genes in both groups. (**c**) qRT-PCR validation of differentially expressed genes. (**d**) Gene Ontology annotation of up-regulated genes by Singular Enrichment Analysis (SEA)

To study the expression variation of A genome in the presence of B chromosomes, we compared the transcriptome of maize lines B73 with and without B chromosomes. Considering the batch effect, we analyzed the gene expression level in the two batches (with 4 and 5 samples) separately. The number of differentially expressed genes (logFC ≥ 3.0) varied between the two batches (Additional file 3: Figure S1), which might be due to the environmental difference. We thus pulled out the differentially expressed genes identified in both batches. Among the 130 differentially expressed genes, 115 were upregulated (Fig. 2a, Additional file 4) and only 15 genes were down-regulated (Fig. 2b, Additional file 4). Of the 115 upregulated genes, 15 were upregulated in B73 + 1B vs. B73 + 0B comparison, 9 in the comparison between B73 + 6Bs and B73 + 1B, and all belonged to the B73 + 6Bs vs. B73 + 0B comparison. Real-time PCR confirmation was also conducted (Fig. 2c). Only a few genes were down-regulated at the presence of B chromosomes, including 13 genes in 6B vs. 0B comparison and 2 genes in 1B vs. 0B comparison (Fig. 2b). Our results indicated that, despite the limited number of differentially expressed genes, the presence of B chromosome does affect the expression of A genome genes and more B chromosomes cause more impact.

We applied AgriGO [33] (Website http://bioinfo.cau.edu.cn/agriGO/) to do functional analysis for genes up-regulated in plants containing B chromosome (Additional file 4). The most significant terms in biological process are cellular process and metabolic process, and the catalytic activity and binding terms are more significant in the category of molecular function (Fig. 2d). Genes up-regulated in + B leaves vs. 0B leaves were significantly enriched in 12 GO terms (Table 1) including hydrolase activity, with most genes involved in ribonucleotide and deoxyribonucleotide binding. The up-regulated genes were mostly involved in basal metabolism.

**Table 1:** Significant GO terms of up-regulated genes

GO term	Ontology	Description	Number in input list	Number in BG/Ref	*p*-value	FDR
GO:0016817	F	hydrolase activity, acting on acid anhydrides	8	1980	0.00077	0.017
GO:0017111	F	nucleoside-triphosphatase activity	8	1822	0.00045	0.017
GO:0016818	F	hydrolase activity, acting on acid anhydrides, in phosphorus-containing anhydrides	8	1958	0.00071	0.017
GO:0016462	F	pyrophosphatase activity	8	1907	0.0006	0.017
GO:0005524	F	ATP binding	12	5133	0.0041	0.046
GO:0032559	F	adenyl ribonucleotide binding	12	5137	0.0041	0.046
GO:0032555	F	purine ribonucleotide binding	13	5800	0.0038	0.046
GO:0032553	F	ribonucleotide binding	13	5800	0.0038	0.046
GO:0001883	F	purine nucleoside binding	12	5398	0.0061	0.046
GO:0001882	F	nucleoside binding	12	5400	0.0062	0.046
GO:0017076	F	purine nucleotide binding	13	6079	0.0058	0.046
GO:0030554	F	adenyl nucleotide binding	12	5398	0.0061	0.046

Note: Total query of input is 40, and the total background number is 39203. BG/Ref = Background/Reference, *p* < 0.01, FDR: False Discovery Rate

### B-located pseudogenes/genes show high similarity to their A chromosome counterparts

Since there are many more upregulated genes than downregulated genes, especially in the 6B libraries, we asked whether any of the upregulated genes also express from the B chromosome. However, it has been always difficult to discriminate those genes that are located on both A and B chromosomes by sequences analysis. Fortunately, an oat-maize-addition (OMA) line carrying a maize B chromosome with Starter line background, a valuable tool for genetics studies of the complex, duplicated maize genome in an alien background [34], making it possible (Additional file 5: Figure S2).

First, we selected thirteen most upregualted A genome gene sequences for primer designing, and then amplified with these primers using B73 + 0B, B73 + 1B, Starter and Starter + B as templates. In total, 11 pairs of primer from 10 genes got amplification in the OMA line containing maize B chromosome, hereafter Starter + B, but with no product in the OMA background line Starter. GRMZM2G702253-2 F/2R generated three different sized bands in Starter + B. Except the smallest one that was amplified from Starter genome, the other two bands were likely amplified from B chromosome. The other 10 pairs of primers designed from 9 A chromosome genes got amplifications with the same size as their A-genome counterparts (Additional file 6: Figure S3), so they might be located on B chromosome. And the rest 4 pairs of primers from three genes had no amplification in both Starter and Starter + B, which might be due to their absence on B chromosome or the sequence variations on the primer sites. We sequenced the PCR products amplified from Starter + B and then blasted against the B73 genome (Additional file 7: Table S3; Additional file 8). Among the 12 B-located fragments, 11 had best alignments to their A-genome homologous genes from which we designed primers, while the GRMZM2G165298_2 had best hits to AF466202.2_FG007, which has high sequence similarity to the GRMZM2G165298. Since all these genes have many paralogous genes in the A-genome, we used specific primers to amplify regions in the four genes with B73 + 1B gDNA as a template, which should amplify both the A-genome and B-genome genes. As expected, the sequencing graph of B73 + 1B has double peaks in the SNP sites or in the upstream/downstream near nucleotide deletion site, confirming that those genes are both A- and B-located (Additional file 9: Figure S4).

Furthermore, we generated full length of four B-located genes (Additional file 8) with the Starter + B and cloned into plasmid for sequencing and FISH assay. We blasted the four sequences against the maize reference genome, and designated the B-located loci as GRMZM2G013761B, GRMZM2G356718B, AF466202.2_FG007B and GRMZM2G356653B according to the most homologous A-genome genes. As shown in Table 2, while each gene shows high similarity to its A-homologue, there are also insertions/deletions and SNPs between the A and B chromosome genes (Additional file 10). We predicted the coding sequence based on the A-genome gene. AF466202.2_FG007B has no change in its coding region, GRMZM2G356653B has only one amino acid substitution in the non-conservative region; the predicted protein of GRMZM2G356718B has multiple amino acid substitutions including conserved domain; and the GRMZM2G013761B shows dramatic change by the SNPs and InDels, which would cause frame shift and premature stop codons, and they likely became nonfunctional pseudogene. To test if these B-located genes are transcribed, we applied Tophat2.0 package at the stringent parameter settings, using the four B-located genes sequence as reference. We indeed found reads that were mapped to the B-specific SNP sites in coding regions with 100 % identity, indicating that they were transcribed from B chromosome (Additional file 11).

**Table 2:** Sequence analysis of four B-located genes

	A-homologues information			
B chromosome loci name	A chromosome gene	Location	A chromosome gene description	Alignment information
GRMZM2G013761B	GRMZM2G013761	chr4:21720959..21725847	DEAD-box ATP-dependent RNA helicase 7	Identity = 99.07 %(4693/4737) Gap = 14.71 %(817/5554)
GRMZM2G356718B	GRMZM2G356718	chr1:281831441..281837582	Myb-like DNA-binding domain	Identity = 99.02 %(5655/5711) Gap = 1.33 %(77/5788)
AF466202.2_FG007B	AF466202.2_FG007	chr10:138520169:138528483	Putative aldose reductase-related protein	Identity = 99.99 %(7677/7678) Gap = 0.01 %(1/7679)
GRMZM2G356653B	GRMZM2G356653	chr1:178546582..178551203	Conserved mid region of cactin	Identity = 99.77 %(4424/4434) Gap = 0.00 %(0/4434)

The four B-located genes were designated according to their A-homologous genes

**Fig. 3:**
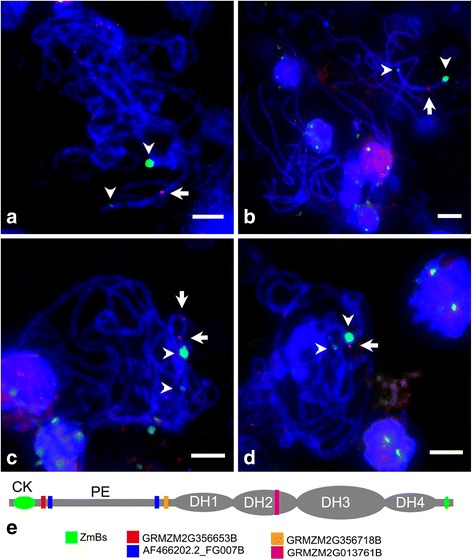
A-homologous genes located on B chromosome. (**a** to **d**) Fluorescence in situ hybridization of B-located genes, pachytene stage chromosomes were probed with plasmids of B-located gene (red) and ZmBs (green). The signals of GRMZM2G013761B appeared on the DH2 heterochromatic region of B chromosome (**a**); the GRMZM2G054938B was located on the proximal euchromatic (PE) region near DH1 side (**b**); AF466202.2_FG007B had two foci on PE region (**c**); and GRMZM2G356653B was close to centromeric knob (**d**). The relative location of these four B chromosome genes were illustrated in (**e**). Arrowheads indicate the ZmBs signals, and arrows indicate the signals of B-located genes. Bar = 10 μm

Then, we did FISH on the B73 + 1B pachytene chromosomes with the four B-located genes as probes. As shown in the Fig. 3, obvious signal of GRMZM2G013761B appeared on the second distal heterochromatic (DH2) region (Fig. 3a), GRMZM2G356718B was located on the proximal euchromatic (PE) region near the DH1 side (Fig. 3b), AF466202.2_FG007B had two loci on the PE region near the centromeric knob and DH1 side (Fig. 3c), and GRMZM2G356653B was located on the PE region close to the centromeric knob (Fig. 3d). The relative locations of these four genes are illustrated in Fig. 3e. Obviously, there are signals on the A chromosome which might be the locations of their A-homologous or paralogous genes. Taken together, our sequencing and FISH results confirmed that there are transcriptionally active genes on maize B chromosome which show high similarity to their A chromosome counterparts.

### Three transcripts derived from B chromosome specific loci

In addition to the location of some A-homologous genes on B chromosome, we ask: are there any transcripts that are B chromosome specific? Since there is high similarity of A- and B-genome, it is hard to distinguish the reads even if they are actually transcribed from the B chromosome. So far, there is no reference sequence of maize B chromosome. Considering that, to pry the unique transcription of maize B chromosome, we utilized the Trinity, a powerful tool to assemble full length transcripts without a reference [35], for *de novo* assembly using the unmapped reads only. Although the maize materials we used were near-inbred-lines (NILs) with a background of B73, there are still some transcripts of B73 + 0B that could not be mapped to the B73 reference genome. To eliminate the interference of those transcripts shared by plants with and without B chromosome, we pooled unmapped reads from all 9 samples for *de novo* assembling. The assembly would generate more complete transcripts belonging to both with and without B chromosomes groups. As a result, 49 assembled genes and 73 isoform genes were supported by reads and we further validated those candidates.

We blasted the 49 genes and 73 isoform genes sequences against NCBI nr database. In total, 31 genes/isoform genes had no or only partial hits, and they were considered candidates of B-specific transcripts. PCR experiments further confirmed that the candidates were actually B-located. We designed PCR primer pairs with at least one primer locating in the region that has no hits in the nr database, and amplified the genomic DNA of maize NILs with and without B chromosome, oat line Starter and Stater + B. Three fragments (comp75688_c6_seq20, comp30393_c0_seq1, and comp74447_c2_seq14) were amplified in Starter + B but not in Starter (Additional file 12: Figure S5A, S5C and S5D), thus they were actually present on maize B chromosome.

**Fig. 4:**
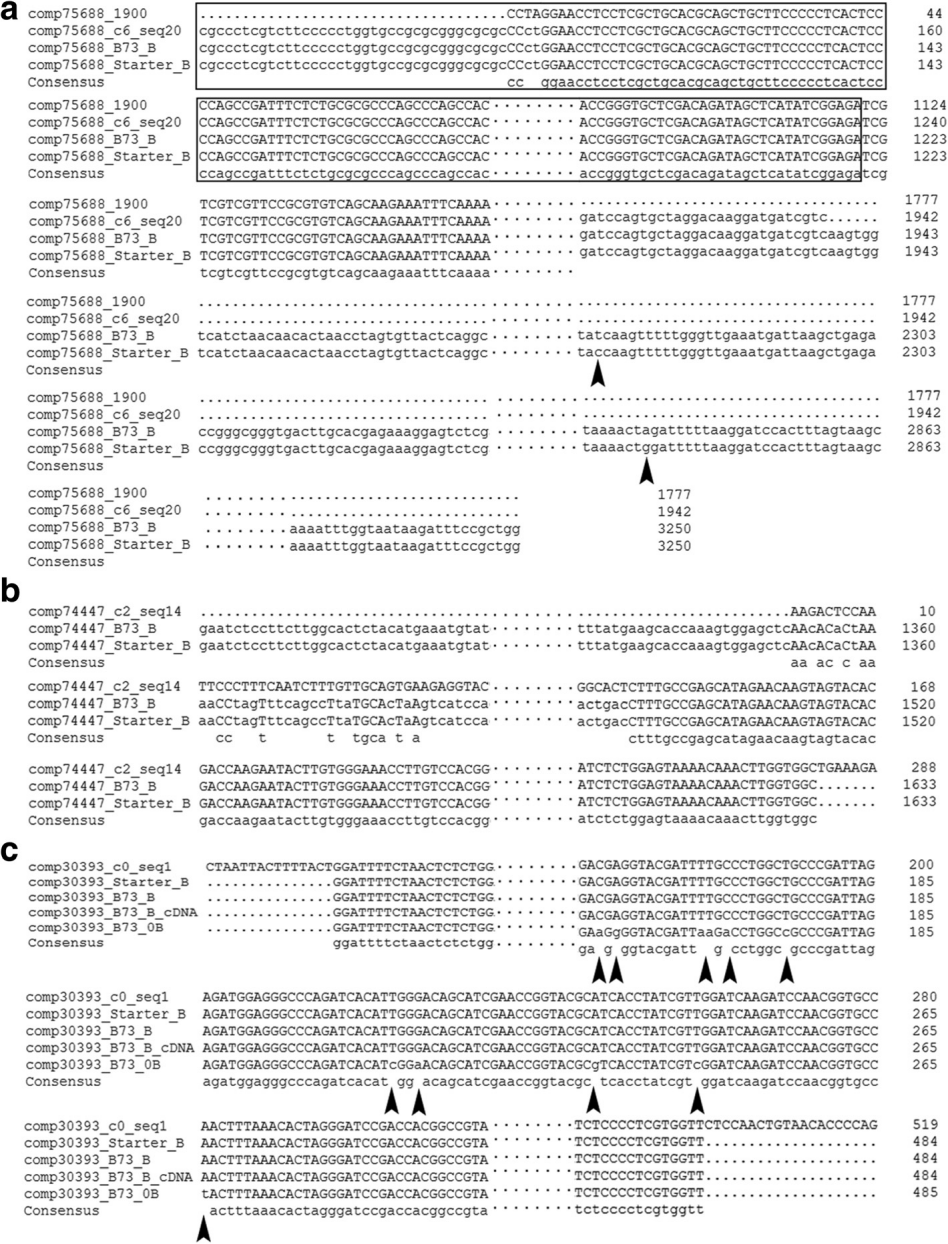
Alignment of 3 B chromosome located sequences. (**a**) Alignment of the assembled sequence comp75688_c6_seq20, the 1900 bp fragment of comp75688, and the full-length comp75688 from B73 + B and Starter + B. Sequence in black box was the newly discovered B-specific sequence. (**b**) Alignment between the *de novo* assembled sequence and 1.6 kb B-located sequence. (**c**) Comparison of the assembled comp30393_c0_seq1, the B-located sequences comp30393_Starter_B and comp30393_B73_B, and the transcribed sequence; these four sequences showed 100 % identity to one another but were significantly different from their A-genome homologues. Arrowheads indicate the SNPs between sequences

The predicted size of comp75688_c6_seq20 is 1,942 bp, part of the sequence has similarity with pB3-201 retrotransposon GrandeB which is a retrotransposon element of B chromosome repetitive sequence *StarkB* [36]. We used the primers comp75688-1 F/4R to amplify this sequence and found that there are two bands in Stater + B and B73 + B (Additional file 12: Figure S5A), the larger one only present in lines plus B chromosome and the smaller one (~500 bp) present in all B73 + 0B, B73 + B and Starter + B lines. Therefore, the two sequences (1.9 kb and 500 bp in length) should be on the B chromosome based on the amplification in Starter + B line. We sequenced the two fragments and found that the 500 bp sequence of B73 + 0B, B73 + B and Starter + B are composed of different fragments in each sample. So we only analyzed the larger one in detail. Sequencing results indicated that the products of B73 + B and Starter + B had 100 % identity to the RNA-seq *de novo* assembled sequences. To generate the larger fragment, we applied 5’-RACE and 3’-RACE with gene specific primers (RACE_comp75688_F and RACE_comp75688_R) located in the newly defined B-specific region. The longest sequence is 3.2 kb (Additional file 12: Figure S5B; Additional file 8). We blasted this sequence against the NCBI nr database, and the best hit is *Zea mays* clone pB3-201 retrotransposon GrandeB (with 85 % identity), while the remaining 1.2 kb sequence is a newly discovered B-specific sequence. We then amplified the 3.2 kb sequence with gDNA of B73 + B and Starter + B and sequenced the products. Sequence analysis of B chromosome sequence comp75688 amplified from B73 + B and starter + B revealed 2 SNPs (Fig. 4a, Additional file 13), which might be generated during the transmission process of the maize B chromosome. PCR and sequencing indicate that the 3.2 kb comp75688 is B-located and transcribed in leaves with B chromosome.

To examine another B-specific transcript candidate, comp74447_c2_14, we designed primers to amplify a 291 bp fragment. However, the PCR product was 1,633 bp and could only be amplified in the presence of B chromosome, namely using gDNA of B73 + B and Starter + B as templates (Additional file 12: Figure S5C; Additional file 8). Sequence analysis indicates that 106 bp of the comp74447_c2_seq14 fully aligned to the amplified sequence (Fig. 4b, Additional file 13). Thus, the 1,633 bp fragment of comp74447 is also B chromosome specific and transcribed in leaves with B chromosome.

Comp30393_c0_seq1 is a 1,355 bp B-derived transcript candidate, with partial hits in maize reference genome database (Additional file 14: Table S4). We only amplified a 484 bp fragment from both maize gDNA and cDNA with- and without-B (Additional file 12: Figure S5D; Additional file 8). The product can also be amplified with oats containing maize B chromosome. As shown in Fig. 4c, the sequences of Starter + B, B73 + B gDNA and cDNA showed 100 % identity with part of comp30393_c0_seq1. The B-derived sequence has several SNPs from the PCR products of B73 + 0B gDNA and cDNA, i.e. its A genome homologue sequence (Additional file 13). Thus, the comp30393 is also located on B chromosome and shows high similarity to its A-genome homologue.

In total, we have identified three maize B chromosome fragments that are transcribed in leaves. We have detected transcripts of comp75688 and comp30393, with the same lengths as their respective genomic sequences.

### Three B-located sequences are LTRs

**Fig. 5:**
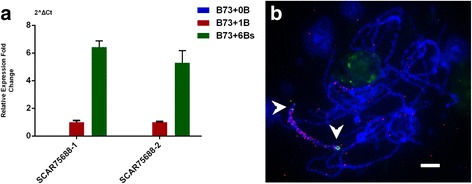
Expression and chromosome location of B-specific fragment comp75688. (**a**) qRT-PCR detected the expression of comp75688 with two SCARs, and comp75688 was expressed in a B-dosage dependent manner. (**b**) FISH detection of the location of comp75688, the 3.2 kb comp75688 was digoxigenin-labeled (red) and the ZmBs was biotin-labeled (green). More condense comp75688 signal was detected on the long arm of the B chromosome. Arrowheads indicate the ZmBs signals. Bar = 5 μm

To investigate these three B-located fragments in detail, we applied RepeatMasker (http://www.repeatmasker.org/cgi-bin/WEBRepeatMasker) to determine if they were repetitive elements. As shown in Table 3, the comp74447 and comp75688 are Gypsy family LTRs and the comp30393 is a Copia family LTR. Thus, all three fragments are non-genic sequences. We did BLAST search of these three sequences against maize reference genome. Part of comp75688 has homology in the maize B73 genome (Additional file 14: Table S4). We then conducted qRT-PCR analysis of comp75688 expression with two sequence characterized amplified region primers (SCARs). While nearly no product was amplified in the B73 + 0B lines, the expression level in the B73 + 6Bs lines was about six fold of that in the B73 + 1B lines (Fig. 5a, Additional file 15: Figure S6). In a previous study, a partial starkB was reported to be expressed [27]. These researchers used northern hybridization and RT-PCR to detect several B-derived transcripts. They failed to amplify any products with the cDNA generated with Oligo-dT priming, and they assumed that StarkB was not poly-adenylated. However, we successfully detected the B-derived transcript comp75688, which might also be part of the tandem StarkB, through RNA-seq, 3’-RACE and Oligo-dT priming cDNA. All three methods we applied here require a poly-adenylated tail, which suggests that the comp75688 we found is a newly discovered B-located sequence.

**Table 3:** RepeatMasker analysis of the three B-located fragments

					Position in query					Position in repeat		
Score	% div.	% del.	% ins.	Query sequence	Begin	End	(left)	Matching repeat	Repeat class/family	(left) begin	End	Begin (left)
387	35.3	2.3	2.7	comp74447	330	588	(1045)	Gypsy-196_ZM-I	LTR/Gypsy	(8584)	1386	1129
1980	27.5	2.4	3.7	comp74447	578	1446	(187)	Gypsy-207_ZM-LTR	LTR/Gypsy	(2160)	1919	1102
291	35.5	4.1	4.6	comp30393	39	431	(53)	PREM1A_ZM_LTR	LTR/Copia	(1836)	1572	1182
8117	4.7	2.6	0.6	comp75688	1974	3009	(241)	Gypsy67-ZM_LTR	LTR/Gypsy	2581	3638	(423)

Score: Smith-Waterman score of the match

% div.: % substitutions in matching region compared to the consensus

% del.: % of bases opposite a gap in the query sequence (deleted bp)

% ins.: % of bases opposite a gap in the repeat consensus (inserted bp)

Furthermore, we conducted FISH on the pachytene chromosomes of B73 + B using the 3.2 kb full length of comp75688 as the probe. As shown in Fig. 5b, while the signals of comp75688 diffused along all the chromosomes, the signals on the whole long arm of B chromosome were much more condensed, which is in agreement with the inference that about half of the comp75688 is B-specific and the rest is partially homologous to the A-genomic sequence. Together, the three B-specific sequences are LTR sequences, and one of them, comp75688, was expressed in a B-dosage dependent manner.

## Discussion

Although the extra chromosomes are neither essential for growth and development nor conferring any obviously benefits to their hosts [37], their existence would affect the A-genome in some way [22, 23]. Studies on rye and maize B chromosome transcription incidentally revealed several A-derived transcripts that are affected by the presence of B chromosome [30, 32]. However, no detailed investigation on the differential gene expression in the presence of B chromosomes has been conducted in plants, especially at whole genome level. Although several maize B-located sequences were found, they are either repetitive elements or short gene-like fragments. Limited sequences on B chromosome are not enough to illustrate the relationship between maize A and B chromosome.

In our study, we employed RNA-seq of B73 NILs with and without B chromosomes and found that the maize gene expression changed in the presence of maize B chromosome. In total, two batches of RNA-seq data revealed 115 genes as being upregulated but only 15 down-regulated in + B vs. no B chromosome plants. On the other hand, only 15 genes were upregulated between 1B vs. 0B. Therefore, lower copy number of B chromosome had less influence on the expression of A genome. Our results are in agreement with that low B chromosome number does not cause significant phenotype variation and that phenotypic effect becomes obvious as copy number increases. For example, maize plant reduces fertility and vigor when the B chromosome is present in many copies [24]. In our study, we also found the transcription of B chromosome genes. Since the B-genes are highly similar to their A genome homologues, which might cause the erroneous mapping of some B-derived transcripts to A chromosome genes. Therefore, the upregulated gene number might be overestimated in our study, it could cause the discrepancy between our results and the previous cDNA-AFLP studies, which showed a negative effect of the B chromosome on the expression of A chromosome located genes [32, 38]. It is unclear whether the upregulation is directly contributed by the transcripts derived from B chromosome or A chromosome genes are upregulated in the presence of B chromosome. However, in the presence of B chromosome, not all the differentially expressed genes were upregulated and some were also downregulated in our study. Furthermore, not all upregulated genes were altered in a dosage dependent manner. Therefore, we propose both scenarios might exist, which might be similar to the case in aneuploids, the expression of identical A- and B-genes might be affected not only by gene dosage effect, but also by the direct trans effect [39].

Among the total 115 upregulated genes, five genes were upregulated in the comparison between + B plants vs. no B chromosome plants and between B chromosome plants with different number of copies. Four of the five genes have protein annotation, GRMZM2G113652 encodes a kinesin motor domain containing protein which is involved in microtubule-binding, GRMZM2G013761 is a DEAD box RNA helicase coding gene which is involved in various aspects of RNA metabolism, and GRMZM2G356718 encodes a TSL-kinase interacting protein playing a role in chromatin metabolism. GO analysis indicates that these upregulated genes are mainly involved in the cell metabolism and nucleotide binding (Table 1, Fig. 2d). Low copy of B chromosome might not lead to significant phenotype, and the existence of extra B chromosomes might be more energy consumption and require more nucleic acid metabolism activity.

Using oat-maize-addition line carrying a maize B chromosome, we amplified 12 gene fragments of 10 genes and further generated four ~5 kb full-length genes/pseudogenes with the primers designed from the A-genome genes (Additional file 8). FISH assay with the four 5-kb genes as probes showed that three genes are located on the proximal euchromatic region and one is on DH2 region, and the signals of four genes also appeared on A chromosomes with multi foci (Fig. 3a to d). Sequence analysis indicated that they have homologous genes on chromosomes 1, 4 and 10, respectively (Table 2), and that the seven relatively short fragments might also have been derived from A-genome genes, with their A-homologues located on chromosomes 1, 3, 8 and 9 (Additional file 7: Table S3). In addition to the A-derived genes, we found three B-located LTR sequences following *de novo* assembly of the unmapped reads (to B73 reference genome) by Trinity. The three LTRs are comp75688 (3250 bp in length), comp74447 (1,633 bp in length) and comp30393 (484 bp in length), each showing partial homology to A-genome sequence (Additional file 14: Table S4; Additional file 8). Therefore, the distribution pattern of B-located sequences with A-homologues indicates that the B chromosome is most likely formed in a chimeric manner. Sequence analysis of B located A-homologous genes and LTR sequences supports the hypothesis that maize B chromosome might be derived from A chromosomes [17, 20, 32, 38, 40, 41]. In that way, the B chromosome located genes might be retained along with the proto extra chromosome derived from standard A chromosome; alternatively, the A homologous genes or gene fragments might be carried into B chromosome by transposon like helitrons.

Although all A-derived genes show high similarity to the A-genome counterparts, there are some Insertions/Deletions and SNPs between them (Table 2 and Additional file 10). GRMZM2G013761B was dramatically different from the A-genome homologue, and the SNPs and InDels would cause frame shift and premature stop codons, thus changing the protein coding sequences. In other words, the B chromosome genes might become pseudogenes. Pseudogene transcripts can generate endogenous siRNAs or miRNA-binding sites and act as gene regulators [42]. Although some B-located genes have lost protein coding capacity, they might regulate the A-genome genes *in trans*. GRMZM2G356718B also accumulated many amino acid substitutions in its protein sequence which might affect the protein function. While some genes show more conservation with their A-homologues, i.e., between AF466202.2_FG007B and GRMZM2G356653B, prediction showed that they could be typical protein coding genes but less likely to be dosage sensitive. Otherwise they should cause remarkable phenotype [43]. As for the three LTR sequences, the 3’end of comp75688 is homologous to an A genome sequence, while 1.2 kb 5’end sequence is a novel B chromosome specific sequence; comp74447 can only be amplified from B chromosome and shows high divergence from A-genome homologous sequences; although comp30393 can be amplified from both A and B chromosome, the two homologues have multiple SNPs. The B chromosome is inherited independently from the A-genome, and there is no selective pressure during its fast evolutionary process [43] that contributes to the variation observed. Sequencing of microdissected rye B chromosomes revealed that the B chromosome had a higher SNP frequency than their A-homologs [16]. Like in the case of the rye B chromosome, our study suggests that during the rapid evolution of the maize B chromosome, the B-located loci accumulated variation with their A-counterparts. In addition to A-derived genes, the maize B chromosome gained B-specific sequences.

We detected reads in the B73 + 1B RNA-seq libraries that carried B chromosome specific SNPs, suggesting that the maize B chromosome genes are also transcriptionally active (Additional file 11). Furthermore, the three B-located LTRs are transcribed. We identified these LTRs fromB73 + B RNA-seq libraries. In addition, we detected the B-derived transcripts of comp30393 and comp75688 within B73 + B cDNA; especially, the transcription level of comp75688 is positively correlated with the number of B chromosome. Our results, together with previous studies [20, 31, 32], provide evidence that the maize B chromosome genes and retrotransposon loci have transcriptional ability.

Nevertheless, only a few B located genes were validated. It might be due to the following reasons, 1) the maize A- and B-genome may have high similarity, especially in the protein-coding genes, making it hard to distinguish them from their A-genome counterparts; 2) the number of functional genes on B chromosome might be limited. During the independent evolution process, the genic region always accumulates high sequence variations. In this case, some B located pseudogenes might be transcriptionally inactive; and 3) Some B-located genes are expressed in a specific spatiotemporal or genetic background dependent way. In rye and wheat, some B-derived transcripts are confined to specific tissues [30]. In both rye and maize, some B-derived transcripts could only be detected in certain lines [30, 32]. We analyzed the transcriptome of B chromosome and its impact on the A-genome expression using only young leaf tissue in the background of B73, the chance to find more genes is even dimed. It is expected that the ongoing genome sequencing of maize B chromosome combined with RNA-seq data will provide deeper insights into the transcriptome and evolution of maize B chromosome.

## Conclusion

In this study, by comparing the maize leaves transcriptome with varied copies of B chromosome, we found that the maize genes expression was altered in the presence of B chromosome and increasing copies of B chromosome had more influence. Using an oat-maize-addition line, Starter + B, we validated twelve B chromosome located gene fragments and three LTR sequences. FISH results directly illustrated the distribution of four ~5 kb sized genes and a newly discovered retrotransposon, comp75688, on maize B chromosome. The twelve B-located gene fragments had high similarity to their A chromosome counterparts, but also accumulated many sequence variations like SNPs and Insertions/Deletions. Using the B chromosome sequences we assembled as reference, we could detect the B-derived transcripts. We conclude that the B chromosome is transcriptional active and its presence would alter the maize transcriptome. The maize B chromosome contains either A-homologous genes or B-specific retrotransposons.

## Methods

### Plant materials

The maize inbred line containing B chromosome was kindly provided by Dr. James A. Birchler (University of Missouri, Columbia). The seedlings were transplanted into field after the B chromosome was counted with FISH assay, each plant was self-pollinated. The B73 + 0B, B73 + 1B and B73 + 6Bs kernels were selected and isolated from the progenies. A maize B chromosome carrying oat-maize-addition line (Starter background) was kindly provided by Dr. Ralf G. Kynast (Royal Botanic Gardens, Kew) and used with the permission of the Board of Trustees of the Royal Botanic Gardens, Kew.

### The preparation of root tips and meiotic anther for FISH assay

Analyses on root tip chromosomes were performed according to the procedures described previously [44]. The kernels from the B73, B73 + Bs and Oats (Starter line and Starter carrying maize B chromosome) were germinated at 28 °C. After 2 ~ 3 days, root tips were harvested and immediately treated with nitrous oxide at 2 atm for 2 h, fixed in Carnoy’s solution (Ethanol:Glacial acetic acid = 3:1), and stored at −20 °C until use. Tassels of lines contained B chromosome at the meiotic stage were also fixed with Carnoy’s solution and stored at −20 °C.

### FISH/GISH assay

The plasmid containing B chromosome specific repeat ZmBs, B-related fragments and the maize genomic DNA were labeled with digoxigein-11-dUTP (Roche) or biotin-11-dUTP (Vector Laboratoried) via nick translation. The digoxigenin- and biotin-labeled probes were detected by antidigoxigenin antibody conjugated with Rhodamin (Roche) and antiavidin antibody conjugated with fluorescein isothiocyanate (Vector Laboratories), respectively. Sequential FISH/GISH was conducted according to Zhao et al. [45]. Anthers were squashed and then staged with phase contrast microscope, pachytene stage meiocytes were selected for FISH assay. The Olympus BX61 epifluorescence microscope, equipped with a CCD camera (QImaging; RETGA-SRV FAST 1394), was used to capture the FISH/GISH images. The Image-Pro Plus 6.0 software (Media Cybernetics) and Adobe Photoshop CS 3 were used to analyze and adjust the digital images.

### RNA isolation and cDNA amplification

Each maize kernel was cultivated in a climate chamber (28 °C day 16 h, 20 °C night 8 h, 50 % humility). Total RNA was isolated from the second euphylla of plants at the four-leaf stage following the instruction provided in the RNAprep pure Plant Kit (Tiangen, Beijing, China, #DP432). About 1 μg total RNA was used to synthesize the first strand cDNA with Moloney murine leukemia virus reverse transcriptase (Invitrogen) and oligo(dT)18 primers.

### RNA-seq analysis

The RNA-seq library construction was performed according the manufacturer’s protocol in the Illumina Standard mRNA-seq library preparation kit (Illumina) and sequenced from both ends to 101 bases on the Illumina HiSeq2000 platform.

Reads from each sample were aligned to the maize reference genome (version ZmB73_5a.59) using the spliced read aligner TopHat and the gene expression value (FPKM) was generated with cufflink program (Version 2.02) [46]. The edgeR package was used to perform differential expression (DE) analysis [47] with the raw read count that was estimated from ht-seq program [48]. Counts were normalized with TMM (trimmed mean of M-values) provided in edgeR package. A p-value was calculated for each gene based on negative binomial model, and these raw *p*-values were adjusted with FDR (false discovery rate correction) approach. Both adjusted *p*-value (≤0.05) and fold change difference (≥3) were used to determine differentially expressed genes. Genes that have mean low read counts less than 5 CPM (counts per million reads mapped) in numerator condition were filtered out from the list of DE genes.

### Gene ontology analysis

GO enrichment was performed by Singular Enrichment Analysis (SEA) tool in agriGO database (http://bioinfo.cau.edu.cn/agriGO/analysis.php) with default parameters, using the maize genome (zea mays ssp V5a) as annotation.

### B-specific transcript analysis

The unmapped reads from samples with B chromosome were pooled. We did *de novo* assembly with Trinity [49] to identify the B chromosome specific genes. Each gene was blasted against *Zea mays* nucleotide collection (nr/nt) database in National Center for Biotechnology Information (NCBI) website. The B chromosome specific transcripts, which have partial hits or without any hit in the database, were validated by PCR amplification of gDNA and/or cDNA of maize and oats with/without B chromosome.

To generate the full-length B-specific genes, 5’-RACE and 3’-RACE were conducted following the manual of SMARTer RACE 5'/3' Kit (Clontech, CA, USA).

### Real-time qRT-PCR

The qRT-PCR was conducted using SYBR Green PCR master mix (Takara). Three replicates were performed. *ZmActin1* was used as the internal reference to normalize the expression data. Relative expression levels were calculated according to the 2^ΔCt method [50]. The primers are listed in Additional file 16.

### Availability of data and material

All sequence reads have been deposited in NCBI Sequence Read Archive (http://www.ncbi.nlm.nih.gov/sra). The BioProject and SRA accession are PRJNA317037 and SRP072810, respectively.

## Additional files

Additional file 1: Table S1. Summary of RNA-seq read mapping results. (XLSX 9 kb) Additional file 2: Tabel S2. The correlation of RNA-seq data in each of the three groups. (XLSX 9 kb) Additional file 3: Figure S1. Venn Diagram of up-regulated and down-regulated genes in each group (GLMfit, fold change ≥3, FDR = 0.1). (A) Up-regulated genes in group1; (B) Down-regulated genes in group1; (C) Up-regulated genes in group2; (D) Down-regulated gens in group2. (TIF 2204 kb) Additional file 4: Data Set S1. The list of differentially expressed genes. (XLSX 71 kb) Additional file 5: Figure S2. Characterization of maize chromosomes in the Oat-maize-addition line. The green signal is biotin-labeled maize genomic DNA, and the red signal is digoxingenin-labeled ZmBs repetitive elements. There are two maize B chromosomes (arrowhead) in this oat mitosis cell. Bar = 10 μm. (TIF 481 kb) Additional file 6: Figure S3. Amplification gene fragments on maize B chromosome. In each group, the first lane is gDNA of B73, the second lane is gDNA of B73 + 1B, the third lane is gDNA of oats Starter line, and the last lane is gDNA of oat-maize-addition line- Starter + B. The eleven groups used primers designed based on eight A-genome gene sequences, except groups 1, 2, 4 and 5 that have no amplification with Starter + B, the PCR products of Starter + B in the other groups are the same size to the maize gDNA. The last group is amplified with primers designed from oats mRNA sequence (GenBank accession: M73232.1) encoding vacuolar H + −ATPase 16 kDa proteolipid subunit (vatp-P1) protein, amplification generates product only with oats gDNA as the template. (TIF 6469 kb) Additional file 7: Table S3. BLAST analysis of 12 B-located gene fragments. (XLSX 14 kb) Additional file 8: A list of B-located gene fragments, four long length genes and three LTRs. (XLSX 21 kb) Additional file 9: Figure S4. Sequencing graph of PCR products of four A and B chromosome homologous gene fragments. The arrows indicate the double peaks of SNP sites, and the arrowheads indicate the insertion/deletion sites, the double peaks appear on the upstream/downstream of the InDel sites. (TIF 3434 kb) Additional file 10: Multiple Sequences alignment of A- and B-genes. The exons A-genome homologue are shown in red character, and the variation sites are highlighted. (PDF 532 kb) Additional file 11: Data Set S2. Allele specific analysis of B-derived transcripts in B73 + 1B RNA-seq libraries. (XLS 1762 kb) Additional file 12: Figure S5. PCR amplification of B-located sequences. Based on the de novo assembled sequences, we amplified 1.9 kb + 500 bp sequences of comp75688_c6_seq19 (A), 1.6 kb comp74447 (C) and 484 bp comp30393 (D). We amplified the full 3.2 kb comp75688 sequence (B) using primers designed with RACE results. (TIF 3251 kb) Additional file 13: Sequencing analysis of B-chromosome located LTR fragments. (DOCX 18 kb) Additional file 14: Table S4. BLAST results of seven B-located gene fragments. (XLSX 11 kb) Additional file 15: Figure S6. The qPCR validation of comp75688 expression in plants with 0B, 1B and 2Bs. The expression of comp75688 was further detected with another batch of plants containing 0B, 1B and 2Bs, the expression level in 2Bs plants was about 2 fold of 1B plants. (TIF 1335 kb) Additional file 16: Primers used in this study. (XLSX 11 kb)

## Abbreviations

FISH: fluorescent *in situ* hybridization
bp: base pair
SNP: single nucleotide polymorphism
InDel: Insertion/Deletion
GO: gene ontology
OMA line: oat-maize-addition line
LTR: long terminal repeat
