# Supplementary figures and images for: B chromosome contains active genes and impacts the transcription of A chromosomes in maize (Zea mays L.)
Source: BMC Plant Biol. 2016 Apr 16;16:88. doi: 10.1186/s12870-016-0775-7 (PMC4833949; doi:10.1186/s12870-016-0775-7)

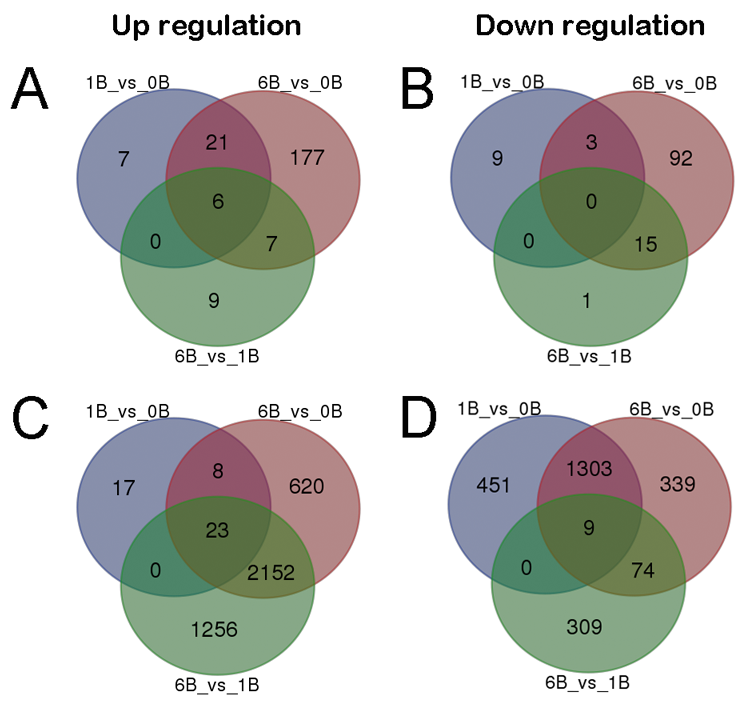

Supplement: Additional file 3: Figure S1. — Venn Diagram of up-regulated and down-regulated genes in each group (GLMfit, fold change ≥3, FDR = 0.1). (A) Up-regulated genes in group1; (B) Down-regulated genes in group1; (C) Up-regulated genes in group2; (D) Down-regulated gens in group2. (TIF 2204 kb) [file 12870_2016_775_MOESM3_ESM.tif]

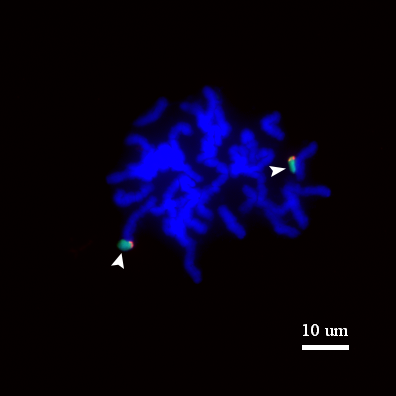

Supplement: Additional file 5: Figure S2. — Characterization of maize chromosomes in the Oat-maize-addition line. The green signal is biotin-labeled maize genomic DNA, and the red signal is digoxingenin-labeled ZmBs repetitive elements. There are two maize B chromosomes (arrowhead) in this oat mitosis cell. Bar = 10 μm. (TIF 481 kb) [file 12870_2016_775_MOESM5_ESM.tif]

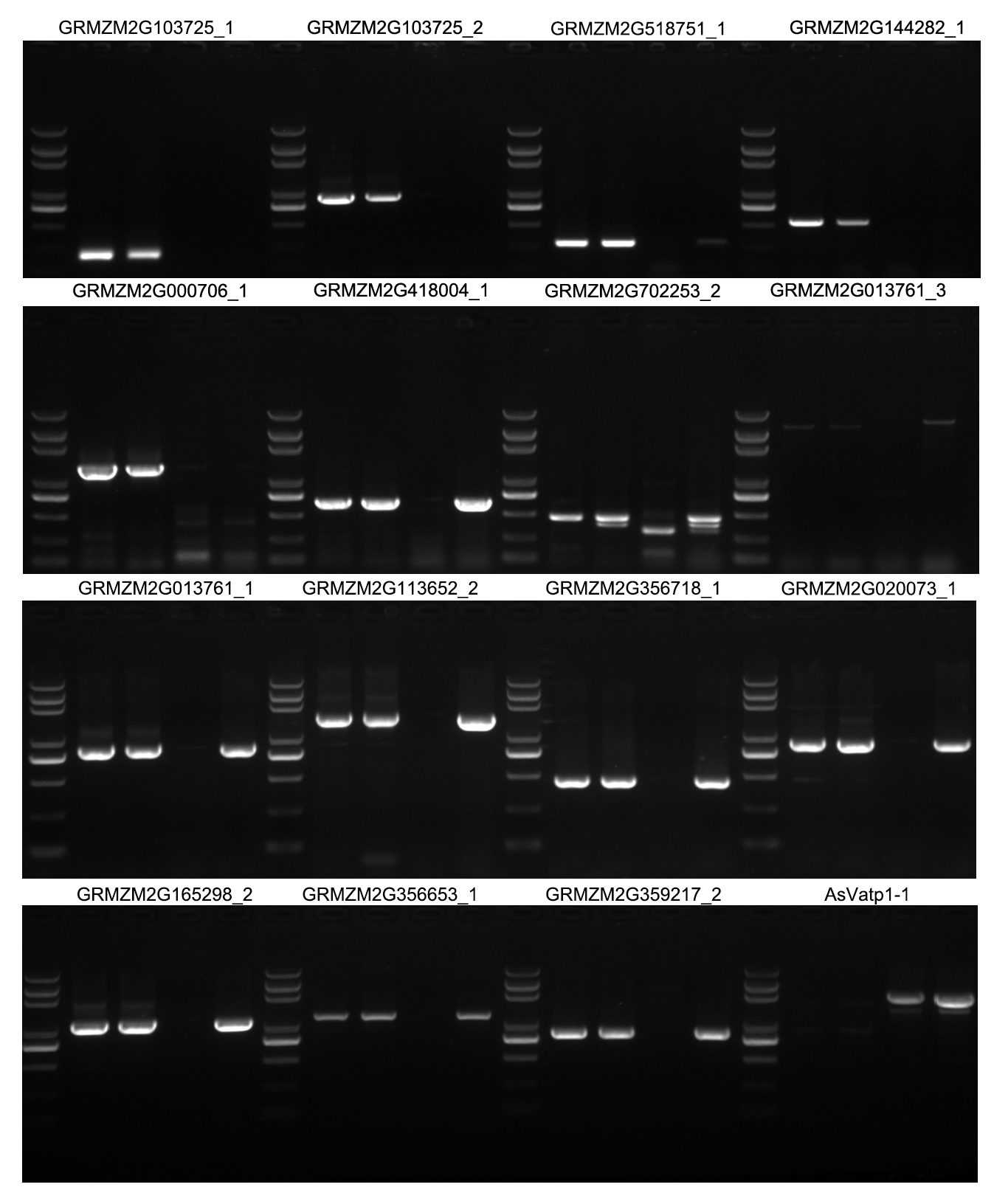

Supplement: Additional file 6: Figure S3. — Amplification gene fragments on maize B chromosome. In each group, the first lane is gDNA of B73, the second lane is gDNA of B73 + 1B, the third lane is gDNA of oats Starter line, and the last lane is gDNA of oat-maize-addition line- Starter + B. The eleven groups used primers designed based on eight A-genome gene sequences, except groups 1, 2, 4 and 5 that have no amplification with Starter + B, the PCR products of Starter + B in the other groups are the same size to the maize gDNA. The last group is amplified with primers designed from oats mRNA sequence (GenBank accession: M73232.1) encoding vacuolar H + −ATPase 16 kDa proteolipid subunit (vatp-P1) protein, amplification generates product only with oats gDNA as the template. (TIF 6469 kb) [file 12870_2016_775_MOESM6_ESM.tif]

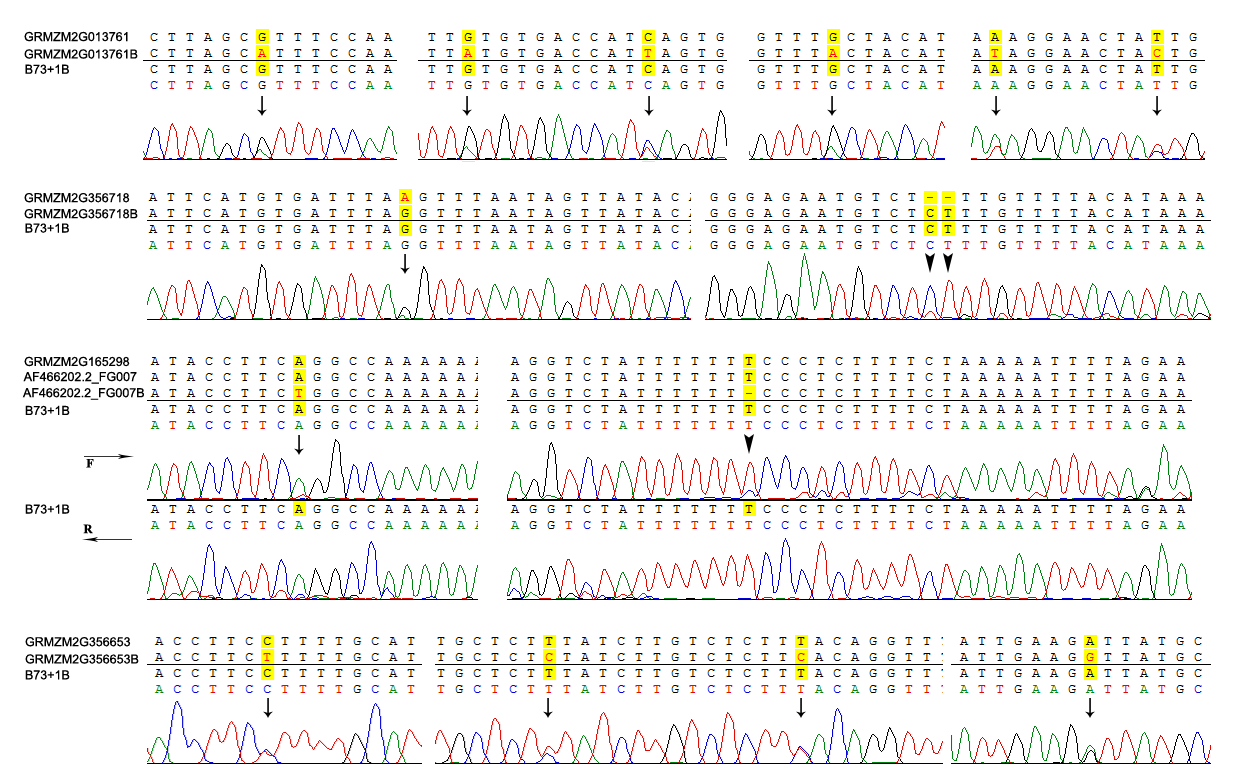

Supplement: Additional file 9: Figure S4. — Sequencing graph of PCR products of four A and B chromosome homologous gene fragments. The arrows indicate the double peaks of SNP sites, and the arrowheads indicate the insertion/deletion sites, the double peaks appear on the upstream/downstream of the InDel sites. (TIF 3434 kb) [file 12870_2016_775_MOESM9_ESM.tif]

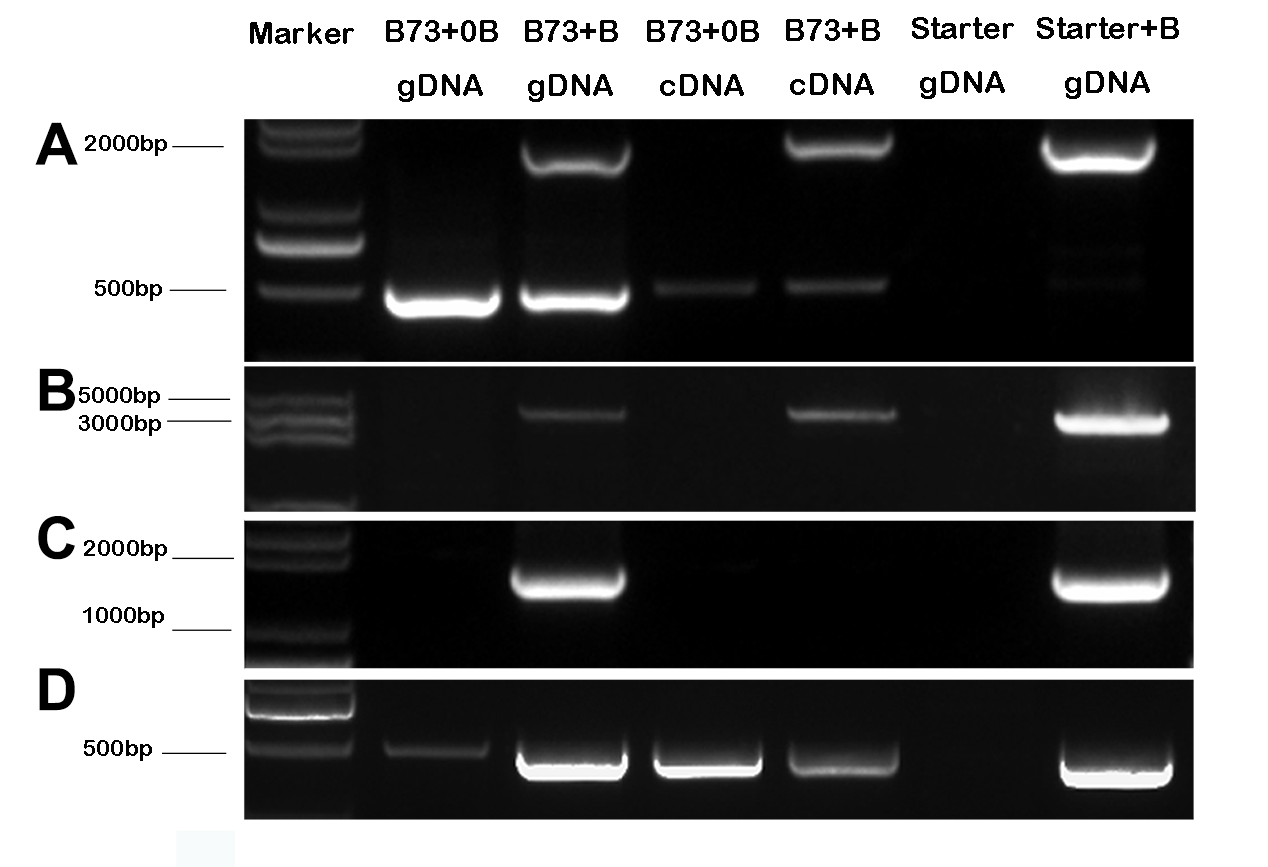

Supplement: Additional file 12: Figure S5. — PCR amplification of B-located sequences. Based on the de novo assembled sequences, we amplified 1.9 kb + 500 bp sequences of comp75688_c6_seq19 (A), 1.6 kb comp74447 (C) and 484 bp comp30393 (D). We amplified the full 3.2 kb comp75688 sequence (B) using primers designed with RACE results. (TIF 3251 kb) [file 12870_2016_775_MOESM12_ESM.tif]

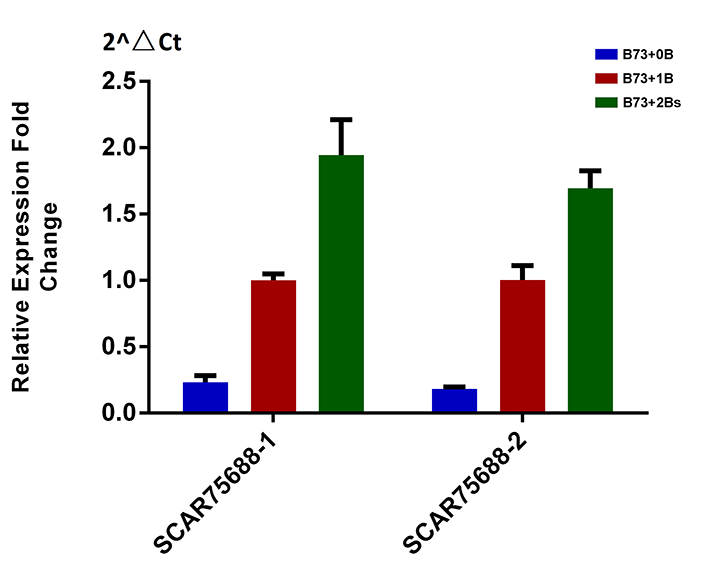

Supplement: Additional file 15: Figure S6. — The qPCR validation of comp75688 expression in plants with 0B, 1B and 2Bs. The expression of comp75688 was further detected with another batch of plants containing 0B, 1B and 2Bs, the expression level in 2Bs plants was about 2 fold of 1B plants. (TIF 1335 kb) [file 12870_2016_775_MOESM15_ESM.tif]
