# Supplementary material for: B chromosome contains active genes and impacts the transcription of A chromosomes in maize (Zea mays L.)
Source: BMC Plant Biol. 2016 Apr 16;16:88. doi: 10.1186/s12870-016-0775-7 (PMC4833949; doi:10.1186/s12870-016-0775-7)
Supplement: Additional file 10: — Multiple Sequences alignment of A- and B-genes. The exons A-genome homologue are shown in red character, and the variation sites are highlighted. (PDF 532 kb) [file 12870_2016_775_MOESM10_ESM.pdf]

Index

Multiple_Sequence_Alignment of GRMZM2G013761 and GRMZM2G013761B.................. 1

Predicted coding sequence and protein sequence of GRMZM2G013761B base on GRMZM2G013761 8

Multiple_Sequence_Alignment of GRMZM2G356718 and GRMZM2G356718B.................. 9

Predicted coding sequence and protein sequence of GRMZM2G356718B base on GRMZM2G356718 17

Multiple_Sequence_Alignment of AF466202.2_FG007 and AF466202.2_FG007B 17

Predicted coding sequence sequence of AF466202.2_FG007B base on AF466202.2_FG007 24

Multiple_Sequence_Alignment GRMZM2G356653 and Grmzm2g356653B 25

Predicted coding sequence and protein sequence of GRMZM2G356653B base on GRMZM2G356653 31

Multiple sequence alignment of four B-chromosome genes, the exons A-genome homologue are shown in red character, and the variation sites are highlighted.

Multiple_Sequence_Alignment of GRMZM2G013761 and GRMZM2G013761B

```
GRMZM2G013761  TCTTCCTCAAGCTCTAGCCGAACTCCCTCACGCCATCCTC     40
GRMZM2G013761B ----------------------------------------     40

GRMZM2G013761  TTTCCTGGTGTTCATCCCTGCGGCAACCACCACTTGGGCG     80
GRMZM2G013761B ----------------------------------------     80

GRMZM2G013761  CTCCTCTCCCTGCCCCTCCCATGGCGGCTCCCCCTGGATC    120
GRMZM2G013761B ----------------------------------------    120

GRMZM2G013761  CCTCCCTGCGCGGCCCCTCTCCCGTGGATGGTGCCGCCCC    160
GRMZM2G013761B --------t----a--------------------------    160

GRMZM2G013761  TTCCTGCAGCACGTCGTGCTCCCTCCCCTGGCGCCCTCGT    200
GRMZM2G013761B ----------------------------------------    200

GRMZM2G013761  CCCCAACGCCGGAAATGCCGCTGCTGCTCCACCAGCCGCT    240
GRMZM2G013761B ----------------------------------------    240

GRMZM2G013761  ACTGCTCCTGAACGCCAGTGCCGACCACCATCTCCCATGG    280
GRMZM2G013761B -----------------------------------.----    279

GRMZM2G013761  CGCAGGAGCTCCCCAATCTCCTCTTGCCAGGGCGCCGTCG    320
GRMZM2G013761B ----------------------------------------    319

GRMZM2G013761  CTGAGCTCGCCCTGCCCCAGCCATGGACGCCGTCGGGCCA    360
GRMZM2G013761B ----------------------------------------    359
```

```
GRMZM2G013761  CCCGTCTCCTATCTCCCCTCGCCCTCATCCTCTGGCTGCT   400
GRMZM2G013761B ----------------------------------------   399

GRMZM2G013761  GCCCCTTCCTATTCCTGGTGGCTGAGTCTTCTTCCATGGA   440
GRMZM2G013761B ----------------------------------------   439

GRMZM2G013761  TGCTGGCTGTGCTGCCCTCCCTGGTGCTCACCTCTGCCCC   480
GRMZM2G013761B ----------------------------------------   479

GRMZM2G013761  CTGCGTTGGATGGCCGGTTGCTCCTGCCCTAGGTTTGTCA   520
GRMZM2G013761B ----------------------------------------   519

GRMZM2G013761  AGCGCTAACTCTGCTCCATCTATCCCTGCTCGAATCTTGC   560
GRMZM2G013761B ---c--------a---------------------------   559

GRMZM2G013761  TGATTGGCCCTGCGCAGATTATCCTCATGCCCGGCCGCGG   600
GRMZM2G013761B ----------------------------------------   599

GRMZM2G013761  TACAACAGCAACAGCCATGAAACCCCATCGTGTCGCGCCC   640
GRMZM2G013761B ----------------------------------------   639

GRMZM2G013761  TCGACGTGTTCGACGGAATATCGCAGCACGTGCCGACCCG   680
GRMZM2G013761B --------------a-------------------a-----   679

GRMZM2G013761  ATCTGCAGCCCTGGTTGCGAGGACGTCGAACCCCGGTGAG   720
GRMZM2G013761B ----------------------------------------   719

GRMZM2G013761  CCCCTCGCTAGTAGCCTATTGTTTTCCGCATCCCTAGTGC   760
GRMZM2G013761B ----------------------------------------   759

GRMZM2G013761  TTGATAAAATGTTGAACCGTTGTGTGTCGTTAACCGCAGC   800
GRMZM2G013761B ----------------------------------------   799

GRMZM2G013761  TACCGTCGACGTTGTGCCTCGCGCTTCACCAATGCGATGA   840
GRMZM2G013761B ----------------------------------------   839

GRMZM2G013761  AATGTCTGAGCGAATAGACGGCATGCGACCCGAGTTTGAT   880
GRMZM2G013761B --------------------a-------------------   879

GRMZM2G013761  TCTGCCAGGTTGTTTGATGTGTATTTTACTCATTGTGATT   920
GRMZM2G013761B ----------------------------------------   919

GRMZM2G013761  TAGTTGATG...TGTTTGTATGTGCGAGGTGATGAGAAAA   957
GRMZM2G013761B ---------atg----------------------------   959

GRMZM2G013761  ATAAAGGAGATGCGTCGTTCGATAATAAAGATTAAGTTAA   997
GRMZM2G013761B ----------------------------------------   999

GRMZM2G013761  AATGTAGTTTATGGTGTGAAGAGTGT..CGATAAATATGT  1035
GRMZM2G013761B --------------------------gt------------  1039

GRMZM2G013761  ATGCCATTGTGTGATATGGCATAAGTTAGAATTAAGGCGA  1075
GRMZM2G013761B ----------------------------------------  1079

GRMZM2G013761  GTATGTATTGTATTATGAAACTAATTAGAGATATGCATGA  1115
GRMZM2G013761B ----------------------------------------  1119
```

```
GRMZM2G013761  TGCTTGTAAGTGGATTGTACGCAGAGTACTTTGGTAAGGG  1155
GRMZM2G013761B ----------------------------------------  1159

GRMZM2G013761  TATTTCTCAAGTATAAATGATTTGGTTATTGTATATGAAT  1195
GRMZM2G013761B ----------------------------------------  1199

GRMZM2G013761  TGAGTGATCAAGTTAATAATGTAAAAACAACGTTTAGTAC  1235
GRMZM2G013761B ----------------------------------------  1239

GRMZM2G013761  TTATAGCGCTTTGCCGACAAGATTTATTGGTGGTTGAGTG  1275
GRMZM2G013761B ----------------------------------------  1279

GRMZM2G013761  AATTTCTAATGTATGGTGACATGAATGCCTTGTTGTATTA  1315
GRMZM2G013761B ----------------------------------------  1319

GRMZM2G013761  TGTGGTTTTAGTTCTACTAAAGATAAAGTGTTGAGTGTTC  1355
GRMZM2G013761B ----------------------------------------  1359

GRMZM2G013761  TAAATGTTAAAAGCATGATAAGTGTAGAAGTTAAATAAGA  1395
GRMZM2G013761B ----------------------------------------  1399

GRMZM2G013761  TCTAAGTTAGAGTTCTTGCTGTTTGGACTGCATGTCCAAT  1435
GRMZM2G013761B ----------------------------------------  1439

GRMZM2G013761  TTTATGACGTGGCTAGTTAAGTAAAGTAAACCGTGTTTTC  1475
GRMZM2G013761B -----t----------------------------------  1479

GRMZM2G013761  TGTAGAAATGTAATATATAAAACTTGTAGATAATCTTTTT  1515
GRMZM2G013761B -----------t----------------------------  1519

GRMZM2G013761  ATCTTTCTTGTGTAAAAATTGCATGACCATAGGTTCAGTA  1555
GRMZM2G013761B -----g--------------t-------------------  1559

GRMZM2G013761  GTTTAGGAGTTATGATTTTTCCAAGTCCAGTTTCAGAATC  1595
GRMZM2G013761B ----------------------------------------  1599

GRMZM2G013761  TGTCCAGATTCTGTACTGATTTCGAAAATGACCTTGTTTG  1635
GRMZM2G013761B ----------------------------------------  1639

GRMZM2G013761  TCCAAGTTAAACTGAGAATTAGCTCTTAGTAATTATAGGA  1675
GRMZM2G013761B ----------------------------------------  1679

GRMZM2G013761  TAATTGTAGTACTTTTCTTAAGCTTTCCAACAAATTTTGG  1715
GRMZM2G013761B g---------------------------------------  1719

GRMZM2G013761  ATCACCCTTTTTGGTGATCTATAAATTAAGTTACAGCTGT  1755
GRMZM2G013761B ----------------------------------------  1759

GRMZM2G013761  TTGAGTTAGTATCTCTGAATCTATCCAAATCTGGACAGCA  1795
GRMZM2G013761B ----------------------g-----------------  1799

GRMZM2G013761  ATGTTTGTTTGTATTAACTGACTTCGATACACATTGAATC  1835
GRMZM2G013761B ----------------------------------------  1839

GRMZM2G013761  ACTTTGAGATGATTATAAATGAATTATAGACAATTCTATT  1875
GRMZM2G013761B ----------------------------------------  1879
```

```
GRMZM2G013761   AGCTTTCTAAAAAGTCTAGGATCACCTGATTTGGATTTAT   1915
GRMZM2G013761B  ----------------------------------------   1919

GRMZM2G013761   GAAACTCCAGTTATGGATTTTTGAAGTGAAGAGCCGAATT   1955
GRMZM2G013761B  ----------------------------------------   1959

GRMZM2G013761   CTGTCCAAATCTGGACAGAATTAAAGTTTAGCACTGTTTG   1995
GRMZM2G013761B  -----t----------------------------------   1999

GRMZM2G013761   ACCATTCAAACTGTTGAATCATGTTGTGATGATAATATAA   2035
GRMZM2G013761B  ----------------------------------------   2039

GRMZM2G013761   GAGTTATAGGACAAGTTTGGGAGCCACAAAACCGGAGAGG   2075
GRMZM2G013761B  ---------...............................   2048

GRMZM2G013761   ATTCCAGAGGCTAAATTCTCCTTCTTATTTAAAATTGAAT   2115
GRMZM2G013761B  ........................................   2048

GRMZM2G013761   AAAAAAGGGAATTTATCCTCTCCAATCCTCTCCGGGTTTA   2155
GRMZM2G013761B  ........................................   2048

GRMZM2G013761   TAGCTCCCAAACTAGCTCATAGAGGACTTTCTAAGCTTTC   2195
GRMZM2G013761B  ......................------------------   2066

GRMZM2G013761   CAGAAAGTCCTAGTTTACTATTTTTGGATGCATATTTTGT   2235
GRMZM2G013761B  ----------------------------------------   2106

GRMZM2G013761   GAGTTATGAATAAAACAATTAATCGTTATGCTGTTGTCTA   2275
GRMZM2G013761B  ---------------------------g-----c------   2146

GRMZM2G013761   CATGTTTTTGTGCATGTCCGAGAACGGTTGCTCGACTTCT   2315
GRMZM2G013761B  -------------------------a--------------   2186

GRMZM2G013761   TCATGAATGAGGTGATAGACATTAGTGGATCATGCAGGGT   2355
GRMZM2G013761B  ----------------------------------------   2226

GRMZM2G013761   TGAGGGTGCTTCATATAACTAAATCTAGCATGGCTTCCTA   2395
GRMZM2G013761B  ----------------------------------------   2266

GRMZM2G013761   GGTAAGATGTTACATTCATATAACCTTTTACATCATATGC   2435
GRMZM2G013761B  ----------------------------------------   2306

GRMZM2G013761   CACATTGTGAGGTGTTCATCTGTGTCTAACTAATAATTAT   2475
GRMZM2G013761B  ----------------------------------------   2346

GRMZM2G013761   TGTGTTCTCCTATTTGTTGTACAGGAAAAGATGGAATGGC   2515
GRMZM2G013761B  ------t---------------------------------   2386

GRMZM2G013761   CACACCAGTGTCGAGACTTGATTTCATATATATTTAGTTA   2555
GRMZM2G013761B  ----------------------------------------   2426

GRMZM2G013761   GGCACAACGATAGCGCAAACAGTTGATTACCAAATTTGTC   2595
GRMZM2G013761B  ---------------------------g------------   2466

GRMZM2G013761   AATTAGTATTTTAGTTTGGATATAGAGTATGCTTATGCAT   2635
GRMZM2G013761B  -----------------------------c----------   2506
```

```
GRMZM2G013761  GAGCTATTTCACAATGTTTATTTATGAGATATATAATGGT  2675
GRMZM2G013761B ----------------------------------------  2546

GRMZM2G013761  AATTTAGAATGATATCATCCTACTTTCATATATTTGATGG  2715
GRMZM2G013761B ----------------------------------------  2586

GRMZM2G013761  ACATCACATATGCATCTAGATTTTTCTCCAAGTTTTAGCA  2755
GRMZM2G013761B -----------------------------------c----  2626

GRMZM2G013761  TGTAGTACATAGGACTAGGTGTCGATCCGGATTGTGGCGC  2795
GRMZM2G013761B --------------------------------------a-  2666

GRMZM2G013761  CCGACGAGGTGGGGGCGTCGTTTGAGAGGGCCGATGAGGT  2835
GRMZM2G013761B ----------------a-----------------------  2706

GRMZM2G013761  GATCTTCGAGGAG...........................  2848
GRMZM2G013761B -------------gaggaagatgatggacagacaacgagg  2746

GRMZM2G013761  ........................................  2848
GRMZM2G013761B acacaccccgaagacccctcacctatcactagcggcgtgg  2786

GRMZM2G013761  ........................................  2848
GRMZM2G013761B agagtggcagggaatcctcatttttttgtactgacggtacg  2826

GRMZM2G013761  ........................................  2848
GRMZM2G013761B atatacaacaggtatgtactgtctttgattgtgctttcgt  2866

GRMZM2G013761  ........................................  2848
GRMZM2G013761B ttaaaaggtagttttgtcagttcacattaccctgcaactt  2906

GRMZM2G013761  ........................................  2848
GRMZM2G013761B attaattactatatctgttgaaaggagttgtctattatct  2946

GRMZM2G013761  ........................................  2848
GRMZM2G013761B gcaacttcacacagcagttcaatacagacaagacaaaaaa  2986

GRMZM2G013761  ........................................  2848
GRMZM2G013761B aaactaaacaaagcattggcaaaggtgacctaccacaaaa  3026

GRMZM2G013761  ........................................  2848
GRMZM2G013761B aatatattcttaacagacaataagattatttcaggctctg  3066

GRMZM2G013761  ........................................  2848
GRMZM2G013761B ttcatcctactaacatttatttagacatataccgctacg  3106

GRMZM2G013761  ........................................  2848
GRMZM2G013761B cttgtaattactactagtttccttgtactactaattttct  3146

GRMZM2G013761  ........................................  2848
GRMZM2G013761B tctcatctctgtttcgttactgcacaggggaacttggtac  3186

GRMZM2G013761  ........................................  2848
GRMZM2G013761B agaaccagccaagctgcaagttgaagaaatcccaagccaa  3226

GRMZM2G013761  ........................................  2848
GRMZM2G013761B gctgaacaggatcttatacagaaccagcagctcccggcca  3266
```

```
GRMZM2G013761   ........................................   2848
GRMZM2G013761B  tggagaaagagaagcaatccgttcctgaatgctcgctcca   3306

GRMZM2G013761   ........................................   2848
GRMZM2G013761B  aaaaaagtgttagttctggatgcctcaactcagcagattg   3346

GRMZM2G013761   ........................................   2848
GRMZM2G013761B  gatgaatggacctgccaggccaaactttgaggcagcattt   3386

GRMZM2G013761   ...........AGCTTACAGTGAAGGAGACATTCAGGTTT   2877
GRMZM2G013761B  gggctgaggag-----------------------------   3426

GRMZM2G013761   GAATGATAAGCATCTCCTTTTCTCCTGTACTGTTCTATTG   2917
GRMZM2G013761B  ----------------------------------------   3466

GRMZM2G013761   TAACAAGGAAGTTGCTATCCTACTCGTCATGGGGAAAAGA   2957
GRMZM2G013761B  ----------------------------------------   3506

GRMZM2G013761   GCGCACTTACAGCGTAAAAGTCTTTTCTTTAACAAGGAAG   2997
GRMZM2G013761B  ----------------------------------------   3546

GRMZM2G013761   TTGCGTTACCCTTTTTTTTATTAGGTCATTACTTTAGCTG   3037
GRMZM2G013761B  ----------------------------------------   3586

GRMZM2G013761   ATCCATGCCTTTCTATTCTGCACAGTATTCTGTTTTCTTT   3077
GRMZM2G013761B  ----------------------------------------   3626

GRMZM2G013761   CAAGTTGGCTGCTTTTGTATGCAAGACAAGATAGTGGTAC   3117
GRMZM2G013761B  ----------------------------------------   3666

GRMZM2G013761   ATGTGTTGTCGCGATTCTGTGTTGAAGGCATGAATGCGAA   3157
GRMZM2G013761B  ----------------------------------------   3706

GRMZM2G013761   AATCTGGTAGATGCTTATTAATTTGCTAGGCACTTAGCGT   3197
GRMZM2G013761B  --------------------------------------a-   3746

GRMZM2G013761   TTCCAATTTTGACAGAACCTTGGAGCCAACACGCCTCGAC   3237
GRMZM2G013761B  ----------------------------------------   3786

GRMZM2G013761   CAGCAGCAAATGTTTGTGTGACCATCAGTGACCTCAATAC   3277
GRMZM2G013761B  ---------------a---------t--------------   3826

GRMZM2G013761   TGTGTTGATTTGCTAGTTTGCTACATCCCTCTACAGAGAT   3317
GRMZM2G013761B  -------------------a--------------------   3866

GRMZM2G013761   CATTGCTTTCAGCACTTTAAAATCACATTTTACTTTGTGT   3357
GRMZM2G013761B  ----------------------------------------   3906

GRMZM2G013761   TATTTTCTTGAGAACGTGTTTCATTTATAGTACTTATGTA   3397
GRMZM2G013761B  ----------------------------------------   3946

GRMZM2G013761   GCCTGACAGGCTCCAGTTTACCTGTAGCATCTGAAATTTC   3437
GRMZM2G013761B  ----------------------------------------   3986

GRMZM2G013761   CTCTGCCCAAACCGACTAAAAGAAGCAGAAAACCCAGCAT   3477
GRMZM2G013761B  -------------------------t--------------   4026
```

```
GRMZM2G013761  GTGAATAAATACCTTGTCACAGATCTTCATACTCATCGCT  3517
GRMZM2G013761B -------------------t--------------------  4066

GRMZM2G013761  AGAATTTCTGATATGAAAATTGTATGGTAGTTCCTAGAAA  3557
GRMZM2G013761B ----------------------------------------  4106

GRMZM2G013761  TTGCTATATAATCTGCTGCTCTGACCTGAGAAATTAGTAT  3597
GRMZM2G013761B ----------------------------------------  4146

GRMZM2G013761  AGGGGTAATCTATGTATTAGCGAAAGAGCTACTATGCTCT  3637
GRMZM2G013761B ----------g-----------------------------  4186

GRMZM2G013761  CAGATATAATAAAAAGGAACTATTGTTGAGAAATTTAAGT  3677
GRMZM2G013761B -------------t--------c-----------------  4226

GRMZM2G013761  TTATGTATTTGACTACTATCAGCTATAGTGCAATGTGCAA  3717
GRMZM2G013761B ----------------------------------------  4266

GRMZM2G013761  GTAATAATATAAGCTATTCTGCAGTAATAATTTTTTCTTT  3757
GRMZM2G013761B ----------------------------------------  4306

GRMZM2G013761  CCATCTAACTTGAACTGCTGACAGATATGAAATGAGGTTG  3797
GRMZM2G013761B ----------------------------------------  4346

GRMZM2G013761  AGATATTACAAGGGCAGCTTTTTCCACGAGACCTCGCCTT  3837
GRMZM2G013761B --------t-------------------------------  4386

GRMZM2G013761  GCATGGAGATGTAGCGCAAGACTGTATATTTCTGGTTTGA  3877
GRMZM2G013761B ----------------------------------------  4426

GRMZM2G013761  TTCCTGGATCCCGTGCCTTGCATGGAGATGTAGCGCAAGC  3917
GRMZM2G013761B ----------------------------------------  4466

GRMZM2G013761  GCAGCGTGAAGTATGTGAATTCTTCTTCGATTTAATAACC  3957
GRMZM2G013761B ----------------------------------------  4506

GRMZM2G013761  TTGGCAATCATTTGATTTTCAGGTAATACTGGTGTTGCTG  3997
GRMZM2G013761B ----------------------------------------  4546

GRMZM2G013761  TCATGCTTTATGAGCCCAGATATAAGCACAGTGTCAGCAG  4037
GRMZM2G013761B ----------------------------------------  4586

GRMZM2G013761  ACTAGAAAGGGAGCCTGGGGTTAAATTTAAGCATATCTCT  4077
GRMZM2G013761B ----------------------------------------  4626

GRMZM2G013761  GCGCCACAACCTACTGATGTAGCACAATCTGCTGGCAGTG  4117
GRMZM2G013761B -----------c----------------------------  4666

GRMZM2G013761  AAGCTGCAGATGCCATTGCGAGTGTGTCAGACAGGTAAGA  4157
GRMZM2G013761B ----------------------------------------  4706

GRMZM2G013761  TATTTCCATCATCATTTTGGGACAATCATGTTTCTTTTGT  4197
GRMZM2G013761B ----------------------------------------  4746

GRMZM2G013761  TTATTGATTTGTCATTAAGAATGTGGATGTGTGTCGTGCA  4237
GRMZM2G013761B ----------------------------------------  4786
```

```
GRMZM2G013761  GTGTTATTCCTATCTTCAGGCAGCAAGCAGAGCAGTTGCT   4277
GRMZM2G013761B ----------------------------------------   4826

GRMZM2G013761  AAGCTCTTCCAGTCTGTCTGCAGCTGACTTGCTTGTCAAA   4317
GRMZM2G013761B -----------------------------------c----   4866

GRMZM2G013761  GCACTTGCGAAGGCAGTTGTAAGTTGCTTTCACGTTCAGC   4357
GRMZM2G013761B ----------------------t-----------------   4906

GRMZM2G013761  TTTTCCAGGTTTTTGCTCTGTTCTAGATATTGGCAGTTGG   4397
GRMZM2G013761B ----------------------------------------   4946

GRMZM2G013761  AACTAACAGAAATATGTGTCTTAGGGTTACACGGACATAA   4437
GRMZM2G013761B ----------------------------------------   4986

GRMZM2G013761  AGAAAAGATCATTGTTATCTTTCTTGGAGGATTACAGTAC   4477
GRMZM2G013761B ---------------------c------------------   5026

GRMZM2G013761  ACTACATCTTCAAACTGGCAGACCGATGTGGTCACCTGGG   4517
GRMZM2G013761B ----------------------------------------   5066

GRMZM2G013761  TAAGTGACTGATAATTTGTGCTTCTGGTTCATGAGTGTTC   4557
GRMZM2G013761B ----------g-----------------------------   5106

GRMZM2G013761  TGATTAAATGGAAATCCTTCTCTGCCTGTAGGTTTGCTAT   4597
GRMZM2G013761B ----------------------------------------   5146

GRMZM2G013761  AACATTCCGGTGATCGTGTTTCTTCATCGTCGGGGTCATA   4637
GRMZM2G013761B -------t-----------------------a--------   5186

GRMZM2G013761  TTTGTTCCCATTGGTGTTTTCTTCAGTTTGACGTCGGCAA   4677
GRMZM2G013761B ------------------a---------------------   5226

GRMZM2G013761  CGTGGTCATGGTGACCGGCGGGAGGAACACCGGGTGTGTA   4717
GRMZM2G013761B ----------------------------------c-----   5266

GRMZM2G013761  GGAGTGATCAAGAACTGGGAGAAGCACAAGGGCATCTTCG   4757
GRMZM2G013761B ----------------------------------------   5306

GRMZM2G013761  AGGTCATCGACGTGCTACTTGGAGTTTTTGCATGCTATGT   4797
GRMZM2G013761B ----------------g-----------------------   5346

GRMZM2G013761  CTAGTTTTCTCCTATTTGTTGTACAGGAAAACATAGAATG   4837
GRMZM2G013761B -----------------------------------a----   5386

GRMZM2G013761  GAATTCAAATTTGGTGGTCCTAAAAGTGTGGAGA        4871
GRMZM2G013761B ----------------------------------        5420
```

Predicted coding sequence and protein sequence of GRMZM2G013761B base on GRMZM2G013761

>GRMZM2G013761B_coding_sequence
ATGGCGGCTCCCCCTGGATCCCTCCCTGTGCGGACCCTCTCCCGTGGATGGTGCCGCCCCTTCCTGCAGCACGTC

GTGCTCCCTCCCCTGGCGCCCTCGTCCCCAACGCCGGAAATGCCGCTGCTGCTCCACCAGCCGCTACTGCTCCTG
AACGCCAGTGCCGACCACCATCTCCATGGCGCAGGAGCTCCCCAATCTCCTCTTGCCAGGGCGCCGTCGCTGAG
CTCGCCCTGCCCCAGCCATGGACGCCGTCGGGCCACCCGTCTCCTATCTCCCCTCGCCCTCATCCTCTGGCTGCTG
CCCCTTCCTATTCCTGGTGGCTGAGTCTTCTTCCATGGATGCTGGCTGTGCTGCCCTCCCTGGTGCTCACCTCTGC
CCCCTGCGTTGGATGGCCGGTTGCTCCTGCCCTAGGTTTGTCAAGCCCTAACTCTACTCCATCTATCCCTGCTCGA
ATCTTGCTGATTGGCCCTGCGCAGATTATCCTCATGCCCGGCCGCGGTACAACAGCAACAGCCATGAAACCCCAT
CGTGTCGCGCCCTCGACGTGTTCGACAGAATATCGCAGCACGTGCCAACCCGATCTGCAGCCCTGGTTGCGAGG
ACGTCGAACCCCGCTACCGTCGACGTTGTGCCTCGCGCTTCACCAATGCGATGAAATGTCTGAGCGAATAGACGA
CATGCGACCCGAGTTTGATTCTGCCAGGTTGTTTGATGTGTATTTTACTCATTGTGATTTAGTTGATGATGTGTTTG
TATGTAATACTGGTGTTGCTGTCATGCTTTATGAGCCCAGATATAAGCACAGTGTCAGCAGACTAGAAAGGGAGC
CTGGGGTTAAATTTAAGCATATCTCTGCGCCACAACCCACTGATGTAGCACAATCTGCTGGCAGTGAAGCTGCAG
ATGCCATTGCGAGTGTGTCAGACAGTGTTATTCCTATCTTCAGGCAGCAAGCAGAGCAGTTGCTAAGCTCTTCCA
GTCTGTCTGCAGCTGACTTGCTTGCCAAAGCACTTGCGAAGGCAGTTTTGGAACTAACAGAAATATGTGTCTTAG
GGTTACACGGACATAAAGAAAAGATCATTGTTATCTTCCTTGGAGGATTACAGTACACTACATCTTCAAACTGGCA
GACCGATGTGGTCACCTGGGTTTGCTATAACATTCTGGTGATCGTGTTTCTTCATCGTCAGGGTCATATTTGTTCCC
ATTGGTGTTATCTTCAGTTTGACGTCGGCAACGTGGTCATGGTGACCGGCGGGAGGAACACCGGGCGTGTAGG
AGTGATCAAGAACTGGGAGAAGCACAAGGGCATCTTCGAGGAAAACATAAAATGGAATTCAAATTTGGTGGTC
CTAAAAGTGTGGAGA

>GRMZM2G013761B_Predicted_protein

```
MAAPPGSLPVRTLSRGWCRPFLQHVVLPPLAPSSPTPEMPLLLHQPLLLLNASADHHLHGAGAPQSPLARAPSLSSPCP
SHGRRRATRLLSPLALILWLLPLPIPGG*VFFHGCWLCCPPWCSPLPPALDGRLLLP*VCQALTLLHLSLLESC*LALR
RLSSCPAAVQQQQP*NPIVSRPRRVRQNIAARANPICSPGCEDVEPRYRRRCASRFTNAMKCLSE*TTCDPSLILPGCL
MCILLIVI*LMMCLYVILVLLSCFMSPDISTVSAD*KGSLGLNLSISLRHNPLM*HNLLAVKLQMPLRVCQTVLFLSSG
SKQSSC*ALPVCLQLTCLPKHLRRQFWN*QKYVS*GYTDIKKRSLLSSLEDYSTLHLQTGRPMWSPGFAITFW*SCFFI
VRVIFVPIGVIFSLTSATWSW*PAGGTPGV*E*SRTGRSTRASSRKT*NGIQIWWS*KCGX
```

Multiple_Sequence_Alignment of GRMZM2G356718 and GRMZM2G356718B

```
GRMZM2G356718   ATGAACTTCAACCCTCGCACTACTTCGTCAAGGAGGTGGT    40
GRMZM2G356718B  ----------------------------------------    40

GRMZM2G356718   CAAAGGCATCAACGAGAGCGACCTCCAAGGCTCCACTAGA    80
GRMZM2G356718B  ------------------------------a---------    80

GRMZM2G356718   CGTGGATCAAAGTCTTCGCCACGCACAACGCCCACGAGCG    120
GRMZM2G356718B  ----------------------------------------    120

GRMZM2G356718   CATCACCAAGCTCGAGAACATGTGCTGGCGGATCTGGCAC    160
GRMZM2G356718B  ----------------------------------------    160

GRMZM2G356718   CTCACTCACAAGAAGCAGGTCAGGATTTCGCACGTCTCTT    200
GRMZM2G356718B  -------g--------------------------------    200

GRMZM2G356718   TCGTGGTTATGATCTCTCGCTGCTTGTTCTATTTTGTTAA    240
GRMZM2G356718B  ----------------------------------------    240

GRMZM2G356718   TAATCTTGCTCGCCTCTCGATTCATGTTTTGTATCAGTTT    280
GRMZM2G356718B  ----------------------------------------    280

GRMZM2G356718   GATCTCTATTTTAATTTGATCTCTGTTTTAGTTTGATCTT    320
GRMZM2G356718B  -------------------------------------a--    320
```

```
GRMZM2G356718  TGTTTAGTTTGATTTTTAAGTGTTCTCTGTTTACTGAATG   360
GRMZM2G356718B ----------------------------.-----------   359

GRMZM2G356718  GAGATGCATGATTAATGAAGCGCACACTGCACAACGAGTA   400
GRMZM2G356718B ----------------------------------------   399

GRMZM2G356718  CAATCTA...TACTACACCATAAGGAACTATCGAGGGCGT   437
GRMZM2G356718B -------tac------------------------------   439

GRMZM2G356718  CAATCTCCATCCGGGTACATCTCTACAACTTCACAATCAT   477
GRMZM2G356718B ------------------------g-------g-------   479

GRMZM2G356718  CAATTTGGTACTGCTATTTTTTTGTTATGGTGTTAGGGTT   517
GRMZM2G356718B ---------------g------------------------   519

GRMZM2G356718  TCATCATTGACAGTTTGTGTAATGCTTTATCAGTGTACAC   557
GRMZM2G356718B ----------------------------------------   559

GRMZM2G356718  TAAATCTGCTACTATTTCGAGAATAATTGTAAAAGTTGAA   597
GRMZM2G356718B ----------------------------------------   599

GRMZM2G356718  GCAAGTTCTATGTTCCTTCCTATTTGACTAAAACCCTAAC   637
GRMZM2G356718B ----------------------------------------   639

GRMZM2G356718  ACCCTTGGCCTTTTTATCAGTGCCTTTAGAACCTTCCAGT   677
GRMZM2G356718B ----------------------------------------   679

GRMZM2G356718  GGTATTTGTTTGAGGGGATTTCTGCTTGTTTATTCAATAA   717
GRMZM2G356718B ----------------------------------------   719

GRMZM2G356718  ATGGTTTAGGGTCTCATACATTATAAAACCCTTTATATGG   757
GRMZM2G356718B ----------------------------------------   759

GRMZM2G356718  TGAAATGTGATTTCCTGTTTCGTGAAACGAGCAGTGCATT   797
GRMZM2G356718B ---------------------------t------------   799

GRMZM2G356718  TTTGTAGTAAGCGAATACATAATGTAGTTCTGCTCATGGG   837
GRMZM2G356718B ----------------------------------------   839

GRMZM2G356718  AGATGACCAAGTAGACATCGCTGCATTGGCAACCTGCAGG   877
GRMZM2G356718B ---c------------------------------------   879

GRMZM2G356718  CTGTAGATGAAATGCCATGGCAGTAGCCCATTGATGGACT   917
GRMZM2G356718B -------------------------------------c--   919

GRMZM2G356718  GACTGTGAACAATAATCCAAAGTTCGCACAGCATCCGTTC   957
GRMZM2G356718B ------------------------------a---------   959

GRMZM2G356718  TCTCCCCTTACTGCTGTGCGAAACTGACCACCGAACCCTA   997
GRMZM2G356718B ----------------------------------------   999

GRMZM2G356718  AGTTTGTCTATTTGTATTCGGATCTGATCTAATTAGAAAT  1037
GRMZM2G356718B ---------------------------------------- 1039

GRMZM2G356718  TTATATGTATACCTAATAAATTCGAATTTTCCTTTTCTTT  1077
GRMZM2G356718B ---------------------------------------- 1079
```

```
GRMZM2G356718  TTGCAAAAAGTTATTGGGTGTACATGTGTACACCCATGTC  1117
GRMZM2G356718B ----------------------------------------  1119

GRMZM2G356718  CTACACTGGGTCCG.CCCCCGAGCCTTCTGATCTTAACAG  1156
GRMZM2G356718B --------------c-------------------------  1159

GRMZM2G356718  GAAACCTGGTTCGTTTTTGCAAACTATTTTTTGTATTTTT  1196
GRMZM2G356718B ----------------------------------------  1199

GRMZM2G356718  TCCAGACTCCCTGTTTATGACAGTTCTGGGTTTTGCCTGA  1236
GRMZM2G356718B ----------------------------------------  1239

GRMZM2G356718  TAGCATTTGCTCTGTATCTGATTTCATAGCAAAAAATCAG  1276
GRMZM2G356718B ----------------------------------------  1279

GRMZM2G356718  ACTCGACAATGGGCTGCGTGGACACACCAAGAGGAGGAGA  1316
GRMZM2G356718B ------------------------------...-------  1316

GRMZM2G356718  TTTTTTTCAATGCGCTGCGGCAAGTAGGAAAAGT..AAGA  1354
GRMZM2G356718B ----------------------------------aa--c-  1356

GRMZM2G356718  GGCAGTAAATTATGGATGCAATGCTCTTCTTCTATTTGTA  1394
GRMZM2G356718B ----------------------------------------  1396

GRMZM2G356718  ATCTAATTAGCAAATAATAATCGATTGATGGATAATCCCA  1434
GRMZM2G356718B ----------------------------------------  1436

GRMZM2G356718  TATTGTTTTCCCAGAACTTTGACAAGATTACACACCGTGT  1474
GRMZM2G356718B ----------------------------------------  1476

GRMZM2G356718  TCAGAGTAAAAACAAGGATCAGGTATTTATTTCTCAACCA  1514
GRMZM2G356718B ----------------------------------------  1516

GRMZM2G356718  AGATGTTTGGATGAAACAAATACACATTAGGCTGTGAGCC  1554
GRMZM2G356718B ----------------------------------------  1556

GRMZM2G356718  TGTGAATTTTAGTTGTGTATGAGTTATCCAAAGGAGTTCA  1594
GRMZM2G356718B ----------------------------------------  1596

GRMZM2G356718  TGTTTCTATTGTGATTAGAGTTTATATTTTATATTACTAT  1634
GRMZM2G356718B -t--------------------------------------  1636

GRMZM2G356718  TAAGATGTTTAATTTTTTTCTTGCAACGTTAGCCTACCTTA  1674
GRMZM2G356718B ----------------------------------------  1676

GRMZM2G356718  TTAAAGTTTAATTCTATTGTATATCAATCTAATATAAAAA  1714
GRMZM2G356718B ---------------g------------t-----------  1716

GRMZM2G356718  GATTTAATGCTTTATGTGCCATACAGGTCAGCCATTACTA  1754
GRMZM2G356718B ----------------------------------------  1756

GRMZM2G356718  TTATCGTCTTGTTCGGCGCATGAAGAAGCTACTGGGTCCT  1794
GRMZM2G356718B ---------------a------------------------  1796

GRMZM2G356718  AGGTTCTCACTTGATGCAAAAAACTCCAAGGACACCATTG  1834
GRMZM2G356718B ----------------------------------------  1836
```

```
GRMZM2G356718  CTGCTATGCTTCGCTGGTAATTGAATATATTTTATCATCT   1874
GRMZM2G356718B ----------------------------------------   1876

GRMZM2G356718  GTAAATTGCCTGAGAAAATATAGTTCATTGTTGATTTTAT   1914
GRMZM2G356718B ----------------------------------------   1916

GRMZM2G356718  TCATAAACCATAGGACCAGTAAGACATTCTATGTAGAAAT   1954
GRMZM2G356718B ----------------------------------t-----   1956

GRMZM2G356718  CAATCTGCTAGCTCTTTTTAGGATCATTAGTTTTATCTTT   1994
GRMZM2G356718B ----------------------------------------   1996

GRMZM2G356718  CATAGAGTGCCGCTTGCTGCTACTTATTACCATGAAGAAG   2034
GRMZM2G356718B ----------------------------------------   2036

GRMZM2G356718  TTCCCTTAGTTTTTTAACTGTCAGGAATTCTAAAATCTTA   2074
GRMZM2G356718B ----------------g-----------------------   2076

GRMZM2G356718  CAGGAAGTTCACAGCCATTAAAAGCCCGGAAACACAAATG   2114
GRMZM2G356718B ----------------------------------------   2116

GRMZM2G356718  CACACCCTTGCCAAAAACATCATCGGGACTTCTTTGGTCT   2154
GRMZM2G356718B ----------------------------------------   2156

GRMZM2G356718  ACACCTTACTGCTGTGCGAAATTGACCACCGAACCCTAAG   2194
GRMZM2G356718B ---------------------c------------------   2196

GRMZM2G356718  TTTGTCTATTTGTATTCGGATCTGATCTAATTAGAAATTT   2234
GRMZM2G356718B ----------------------------------------   2236

GRMZM2G356718  ATATGTATACCTAATAAATTCGAATTTTCCTTTTCTTTTT   2274
GRMZM2G356718B ----------------------------------------   2276

GRMZM2G356718  GCAAAAAGTTATTGGGTGTACATGTGTACACCCATGTCCT   2314
GRMZM2G356718B ----------------------------------------   2316

GRMZM2G356718  ACATTGGGTCCGCCCCCGAGCCTTCTGATCTTAACAGGAA   2354
GRMZM2G356718B ---c------------t-----------------------   2356

GRMZM2G356718  ACCTGGTTCGTTTTTGCAAACTATTTTTTGTATTTTTTCC   2394
GRMZM2G356718B -------------------------------------c-a   2396

GRMZM2G356718  AGA....CTCCCTGTTTATGACAGTTCTGGGTTTTGCCTG   2430
GRMZM2G356718B gactacc---------------------------------   2436

GRMZM2G356718  ATAGCATTTGCTCTGTATCTGATTTCATAGCAAAAAATCA   2470
GRMZM2G356718B ----------------------------------------   2476

GRMZM2G356718  GACTCGACAATGGGCTGCGTGGACACACCAAGAGGAGGAG   2510
GRMZM2G356718B ----------------------------------------   2516

GRMZM2G356718  ATTTTTTTCAATGCGCTGCGGCAAGTAGGAAAAGTAAGAG   2550
GRMZM2G356718B ----------------------------------------   2556

GRMZM2G356718  GCAGTAAATTATGGATGCAATGCTCTTCTTCTATTTGTAA   2590
GRMZM2G356718B ----------------------------------------   2596
```

```
GRMZM2G356718  TCTAATTAGCAAATAATAATCGATTGATGGATAATCCCAT   2630
GRMZM2G356718B ----------------------------------------   2636

GRMZM2G356718  ATTGTTTTTTCCAGAACTTTGACAAGATTACACACCGTGT   2670
GRMZM2G356718B ----.----c--------------------------a---   2675

GRMZM2G356718  TCAGAGTAAAAACAAGGATCAGGTATTTATTTCTCAACCA   2710
GRMZM2G356718B ----------------........................   2691

GRMZM2G356718  AGATGTTTGGATGAAACAAATACACATTAGGCTGTGAGCC   2750
GRMZM2G356718B ...................................-----   2696

GRMZM2G356718  TGTGAATTTTAGTTGTGTATGAGTTATCCAAAGGAGTTCA   2790
GRMZM2G356718B ----------------------------------------   2736

GRMZM2G356718  TGTTTCTATTGTGATTAGAGTTTATATTTTATATTACTAT   2830
GRMZM2G356718B ----------------------------------------   2776

GRMZM2G356718  TAAGATGTTTAATTTTTTCTTGCAACGTTAGCCTACCTTA   2870
GRMZM2G356718B ------------------------------t---------   2816

GRMZM2G356718  TTAAAGTTTAATTCTGTTGTATATCAATCTAATATAAAAA   2910
GRMZM2G356718B ----------------------------------------   2856

GRMZM2G356718  GATTTAATGCTTTATGTGCCATACAGGTCAGCCATTACTA   2950
GRMZM2G356718B ----------------------------------------   2896

GRMZM2G356718  TTATCGTCTTGTTCGACGCATGAAGAAGCTACTGGGTCCT   2990
GRMZM2G356718B ---------------g------------------------   2936

GRMZM2G356718  AGGTTCTCACTTGATGCAAAAAACTCCAAGGACACCATTG   3030
GRMZM2G356718B ----------------------------------------   2976

GRMZM2G356718  CTGCTATGCTTCGCTGGTAATTGAATATATTTTATCATCT   3070
GRMZM2G356718B ----------------------------------------   3016

GRMZM2G356718  GTAAATTGCCTGAGAAAATATAGTTCATTGTTGATTTTAT   3110
GRMZM2G356718B ----------------------------------------   3056

GRMZM2G356718  TCATAAACCATAGGACCAGTAAGACATTCTATGTAGAAAT   3150
GRMZM2G356718B ----------------------------------------   3096

GRMZM2G356718  CAATCTGCTAGCTCTTTTTAGGATCATTAGTTTTATCTTT   3190
GRMZM2G356718B ----------------------------------------   3136

GRMZM2G356718  CATAGAGTGCCGCTTGCTGCTACTTATTACCATGAAGAAG   3230
GRMZM2G356718B ----------------------------------------   3176

GRMZM2G356718  TTCCCTTAGGTTTTTAGCTGTCAGGAATTCTAAAATCTTA   3270
GRMZM2G356718B ---------t------------------------------   3216

GRMZM2G356718  CAGGAAGTTCACAGCCATTAAAAGCCCGAAAACACAAATG   3310
GRMZM2G356718B ----------------------------g-----------   3256

GRMZM2G356718  CACACCCTTGCCAAAAACATCATCGGGACTTCTTT.GTCT   3349
GRMZM2G356718B -------------------------a---------g----   3296
```

```
GRMZM2G356718  ACACAAAATATTTTATTATGTCCAAATTCAACAGTTGTGA  3389
GRMZM2G356718B ----------------------------------------  3336

GRMZM2G356718  TATGCAAGTCCTGATCCCATGCAAAATGTTTGCAGGATAA  3429
GRMZM2G356718B ----------------------------------------  3376

GRMZM2G356718  ACGATGCCCCCGCCACCTAAGGATTTGGAGATTGCTATAG  3469
GRMZM2G356718B ----------------------------------------  3416

GRMZM2G356718  TGCAAGTAGAGTGTAGTGATCAATGGGTCCTGATAATGTT  3509
GRMZM2G356718B ----------------------------------------  3456

GRMZM2G356718  CAAAGGCTACAAGCATTAGCCGGGACATAGTCCAAGCCTG  3549
GRMZM2G356718B ----------------------------------------  3496

GRMZM2G356718  ATACAGGAAAAGTGTCACGTGTCACTTAACGTTATGCCCA  3589
GRMZM2G356718B -----t----------------------------------  3536

GRMZM2G356718  ACTTTGTTTTGTTTGTGCTATGAGTTGTATAACTATTTAA  3629
GRMZM2G356718B ----------------------------------------  3576

GRMZM2G356718  GCCCATCTTCAGTTTTTCCCAACTTCAGTTTCACTAGCAT  3669
GRMZM2G356718B ----------------g-----------------------  3616

GRMZM2G356718  TGTCTAGAACACGGTGTTCGATATACATGTTTTATACTAA  3709
GRMZM2G356718B --------------------g-------------------  3656

GRMZM2G356718  ATTTTAACTTCAGTGGCAACGCACGGGCACATACCTATCA  3749
GRMZM2G356718B t---------------------------------------  3696

GRMZM2G356718  ATTGATAACACATATGGATGTCAATTATACTTAAGCAAAC  3789
GRMZM2G356718B ----------------------------------------  3736

GRMZM2G356718  AAAAGGTATGCATCCCCATAAAATACATTTACTAAAAATT  3829
GRMZM2G356718B -------------------------t--------------  3776

GRMZM2G356718  TGTAATTTACAATTAATATTTACTCTTATATTCTTTGTTG  3869
GRMZM2G356718B ----------------------------------------  3816

GRMZM2G356718  CGACAGCAGCGAGCATACATTGAACTAAATTGATACTTGC  3909
GRMZM2G356718B ----------------------------------------  3856

GRMZM2G356718  TTCTCTCTTGTATTCATGTGATTTAAGTTTAATAGTTATA  3949
GRMZM2G356718B -------------------------g--------------  3896

GRMZM2G356718  CATTTGCGAGAGATCTACATGCCATGTGTTGTTTATATCA  3989
GRMZM2G356718B ----------------------------------------  3936

GRMZM2G356718  TGACAGTAAAATAATATTTTGTAAATGAAAATAATTCAAG  4029
GRMZM2G356718B ----------------------------------------  3976

GRMZM2G356718  CCTTCATTTCTGGGTGGCCATGTACACCTTGTTTATGGTG  4069
GRMZM2G356718B ----------------------------------------  4016

GRMZM2G356718  GGAGAATG..TCTTTGTTTTACATAAATGATGGGTGCTGG  4107
GRMZM2G356718B --------tc------------------------------  4056
```

```
GRMZM2G356718  TGGGGATGCCTATCTTATTGTTGCGGGTCGGTGGCGTGAA  4147
GRMZM2G356718B ----------------g-----------------------  4096

GRMZM2G356718  GCCATGCTTGCGAACAGCTTGAAGGACCAGTGGAAAAGAT  4187
GRMZM2G356718B -------a-------------------g------------  4136

GRMZM2G356718  CATTTTTATTATCTTAATGAAACTGTTTTGCGTGCACGAA  4227
GRMZM2G356718B ----------------------------------------  4176

GRMZM2G356718  AACAGTACCATGTATTTGGAAAAAAACCTCCCTATTTTAG  4267
GRMZM2G356718B ---------------------------------g------  4216

GRMZM2G356718  CCATTTCATCCTCCTCTTTTAGACTGAAAAATTCTAGGAT  4307
GRMZM2G356718B ----------------------------------------  4256

GRMZM2G356718  TATTATTGTCTGCCATCTTGATGTTTTATGTGATCTTTAG  4347
GRMZM2G356718B -------------------------------------c--  4296

GRMZM2G356718  GAAAAATAGTGACTAATGGGACTAGAATGTGCTGGTTCAA  4387
GRMZM2G356718B -------------------------------t--------  4336

GRMZM2G356718  AGTTTTGGCCTTGGTATCTTACTGGTTCAATGTGGCACTG  4427
GRMZM2G356718B ----------------------------------------  4376

GRMZM2G356718  CTAGCTATGTATGTGAAAGTCTTTAAAGATGGAAGAAGAA  4467
GRMZM2G356718B ----------------------------------------  4416

GRMZM2G356718  GTTGGCATGGATGGTTAGGGGAGACACTAAAGTTAAATGG  4507
GRMZM2G356718B ----------------------------------------  4456

GRMZM2G356718  TGCTAAAGTTTATCTAAAGCTAGCAATATCATCTACAGTT  4547
GRMZM2G356718B ----------------------------------------  4496

GRMZM2G356718  ATGACCTGGTAAGCTTGATGTGGGGCACCATGTGAAAAAC  4587
GRMZM2G356718B ----------------------------------------  4536

GRMZM2G356718  ATGTATTTCAATAAGTCTTGAATTCGAACTTTATGGTTTG  4627
GRMZM2G356718B ----------------------------------------  4576

GRMZM2G356718  GCAAAGTAGTAATGTCTACCTTTTCCTACTTTCTGCATGG  4667
GRMZM2G356718B --------------------------------a-------  4616

GRMZM2G356718  AACTGGTAATTTGATTTTCAAGATAACTCAGCTTGGAGTA  4707
GRMZM2G356718B ----------------------------------------  4656

GRMZM2G356718  TTGGGCATTTGAGATGGTGGTTCCTAGCGGGATTTCTTTC  4747
GRMZM2G356718B ----------------------------------------  4696

GRMZM2G356718  AGATCCAATACTAGAAAATTCAGTTTTGTCAGTCAGGTAA  4787
GRMZM2G356718B ----------------------------------------  4736

GRMZM2G356718  GTAAGTGAAATTTGAATTGCCTTCTCCCTTTCAGTAATGC  4827
GRMZM2G356718B --------------------------t-------------  4776

GRMZM2G356718  GAAATTCTATTAAACTCATCGATCTTTTAGATCTGATGTT  4867
GRMZM2G356718B -------------------t--------------------  4816
```

```
GRMZM2G356718  GTTCTGTAACATGACTGATCACTGTGTGGTTGCATTAGTT   4907
GRMZM2G356718B ----------------------------------------   4856

GRMZM2G356718  TTCTTTCTCATGTCTTGAGATAGTTGTGTCTTGTGCTTCA   4947
GRMZM2G356718B ----------------------------------------   4896

GRMZM2G356718  ATCATGCATCTCTACTACCTATTAAGAGGCAAGAGAGGGC   4987
GRMZM2G356718B ----------------------------------------   4936

GRMZM2G356718  GTCCACAAGCGACACCATACAATAATCAATCATATTAGCA   5027
GRMZM2G356718B ----------------------------------------   4976

GRMZM2G356718  GACATGATTAATTATATTAAACTTCAATTCTTGTCTCATT   5067
GRMZM2G356718B ----------------------------------------   5016

GRMZM2G356718  GGTGCTCTCGTTTAAGTGTAGTAGTCCTTGTTAAATATAT   5107
GRMZM2G356718B ----------------------------------------   5056

GRMZM2G356718  TGCCATATTAGTAGACATGACTGCATCCTAATCTTCTTTA   5147
GRMZM2G356718B -------------------------c-----------c--   5096

GRMZM2G356718  GCAAATTCATTCGCAGTAAGCTTGTCGTCTTATGCATGTT   5187
GRMZM2G356718B ---------g------------------------------   5136

GRMZM2G356718  TATATTTGAGCAAATCATAATTTACTTGGAGCAACATATT   5227
GRMZM2G356718B ----------------------------------------   5176

GRMZM2G356718  TACCAGGACTTAAGAAGTGAGCACTACGACTTTGCAAAAG   5267
GRMZM2G356718B ----------------------------------------   5216

GRMZM2G356718  TGGTGATAATGATATATCGTAGATTGTCAGTGATGCAATG   5307
GRMZM2G356718B -----------------------------------a----   5256

GRMZM2G356718  AGGTTGTTCCTCGAAGATGCTAATTCCCCTCAAGCATCAC   5347
GRMZM2G356718B ----------------------------------------   5296

GRMZM2G356718  ATGAAAATGAAATATTTGCTAAATATGTTGAGGATTCAGT   5387
GRMZM2G356718B ----------------------------------------   5336

GRMZM2G356718  TCGCAAAGCTTTCTTGCACGGTAACCTTGCTCTTGCTGAT   5427
GRMZM2G356718B -------------------a--------------------   5376

GRMZM2G356718  GTTTCAGTCATCAACCATTCTTCTAGTACAACAATACTGT   5467
GRMZM2G356718B ------------------------g--------g------   5416

GRMZM2G356718  CATTCTTCTGGTACCACAACACTGATAGTATTGATCTATG   5507
GRMZM2G356718B ----------------------------------------   5456

GRMZM2G356718  GGAGGTAAGGTGATAATTTTAATGATTGTTTTAGTGTTGT   5547
GRMZM2G356718B ----------------------------------------   5496

GRMZM2G356718  GATTTAAACCATTCTGATATTTCGTTCATATGTTGTAGGC   5587
GRMZM2G356718B ----------------------------------------   5536

GRMZM2G356718  AATTGTTAGTAGCAAATGCTGAGGATTATCGTACAGTCTT   5627
GRMZM2G356718B ---------------------------------t----c-   5576
```

```
GRMZM2G356718  GTGTAGGAAAGGAATAGCAGTGGATATGTCAAAGGATCAT   5667
GRMZM2G356718B ----------------------------------------   5616

GRMZM2G356718  AGGCCGACATATGATGCAGAGCATCAAAGGGTCATTGAGT   5707
GRMZM2G356718B ----------------------------------------   5656

GRMZM2G356718  GTGGTGGATACATCGAAGATGGCTACCTCAATGGCGTACT   5747
GRMZM2G356718B ----------------------------------------   5696

GRMZM2G356718  GTCTATGACCCGGGCTTTAGGGGACTAG               5775
GRMZM2G356718B ----------------------------               5724
```

Predicted coding sequence and protein sequence of GRMZM2G356718B base on GRMZM2G356718

>GRMZM2G356718B_Coding_sequence
ATGAACTTCAACCCTCGCACTACTTCGTCAAGGAGGTGGTCAAAGGCATCAACGAGAGCGACCTCCAAGGATCC
ACTAGACGTGGATCAAAGTCTTCGCCACGCACAACGCCCACGAGCGCATCACCAAGCTCGAGAACATGTGCTGG
CGGATCTGGCACCTCACTCGCAAGAAGCAGCAAAAAATCAGACTCGACAATGGGCTGCGTGGACACACCAAGA
GGAGATTTTTTTCAATGCGCTGCGGCAAGTAGGAAAAAACTTTGACAAGATTACACACCGTGTTCAGAGTAAAA
ACAAGGATCAGGTCAGCCATTACTATTATCGTCTTGTTCGACGCATGAAGAAGCTACTGGGTCCTAGGTTCTCACT
TGATGCAAAAAACTCCAAGGACACCATTGCTGCTATGCTTCGCTGTTATTGGGTGTACATGTGTACACCCATGTCC
TACACTGGGTCCGCCCCTGAGCCTTCTGATCTTAACAGGAAACCTGCAAAAAATCAGACTCGACAATGGGCTGC
GTGGACACACCAAGAGGAGGAGATTTTTTTCAATGCGCTGCGGCAAGTAGGAAAAAACTTTGACAAGATTACA
CACCATGTTCAGAGTAAAAACAAGGAGCCTGTCAGCCATTACTATTATCGTCTTGTTCGGCGCATGAAGAAGCTA
CTGGGTCCTAGGTTCTCACTTGATGCAAAAAACTCCAAGGACACCATTGCTGCTATGCTTCGCTGCTTGGAGTATT
GGGCATTTGAGATGGTGGTTCCTAGCGGGATTTCTTTCAGATCCAATACTAGAAAATTCAGTTTTGTCAGTCAGAT
TGTCAGTGATGAAATGAGGTTGTTCCTCGAAGATGCTAATTCCCCTCAAGCATCACATGAAAATGAAATATTTGCT
AAATATGTTGAGGATTCAGTTCGCAAAGCTTTCTTGCACAGTAACCTTGCTCTTGCTGATGTTTCAGTCATCAACC
ATTCTTCTGGTACAACAGTACTGTCATTCTTCTGGTACCACAACACTGATAGTATTGATCTATGGGAGGTAAGGCA
ATTGTTAGTAGCAAATGCTGAGGATTATCGTATAGTCCTGTGTAGGAAAGGAATAGCAGTGGATATGTCAAAGGA
TCATAGGCCGACATATGATGCAGAGCATCAAAGGGTCATTGAGTGTGGTGGATACATCGAAGATGGCTACCTCAA
TGGCGTACTGTCTATGACCCGGGCTTTAGGGGACTAG
>GRMZM2G356718B_Protein_sequence
MNFNPRTTSSRRWSKASTRATSKDPLDVDQSLRHAQRPRAHHQAREHVLADLAPHSQEAAKNQTRQWAAWTH
QEEIFFNALRQVGKNFDKITHRVQSKNKDQVSHYYYRLVRRMKKLLGPRFSLDAKNSKDTIAAMLRCYWVYMCTP
MSYTGSAPEPSDLNRKPAKNQTRQWAAWTHQEEEIFFNALRQVGKNFDKITHHVQSKNKEPVSHYYYRLVRRMKK
LLGPRFSLDAKNSKDTIAAMLRCLEYWAFEMVVPSGISFRSNTRKFSFVSQIVSDEMRLFLEDANSPQASHENEIFAK
YVEDSVRKAFLHSNLALADVSVINHSSGTTVLSFFWYHNTDSIDLWEVRQLLVANAEDYRIVLCRKGIAVDMSKDHR
PTYDAEHQRVIECGGYIEDGYLNGVLSMTRALGD

Multiple_Sequence_Alignment of AF466202.2_FG007 and AF466202.2_FG007B

```
AF466202.2_FG007   ....AGCATATGACTGGGTGAGGGCCGACTCCTTCGACTT    36
AF466202.2_FG007B atgc----g-------------------------------    40

AF466202.2_FG007  CGGGACGACTGTCTCGCCCCCCAGGTATCCATCCAGCTCC    76
AF466202.2_FG007B ----------------------------------------    80
```

```
AF466202.2_FG007  TCCTCCTCCTCGCCGCCGCCGCCTCGATCCTGTCCTGGAT   116
AF466202.2_FG007B ----------------------------------------   120

AF466202.2_FG007  TGGAGTCTCACCCTACCCCTCTAACAAAGTAACAATCTCA   156
AF466202.2_FG007B ----------------------------------------   160

AF466202.2_FG007  TGATTTTGCGATTTCGTGCAACGGGCACACTGCTGTTATC   196
AF466202.2_FG007B ----------------------------------------   200

AF466202.2_FG007  ATTAGCAAGTCCAAGGTGGTCCGTCTTCGGCGGATTCACC   236
AF466202.2_FG007B ----------------------------------------   240

AF466202.2_FG007  GACAAGCGCTTCCTCGCTGATGTTTCCGTCTACGACGTAG   276
AF466202.2_FG007B ----------------------------------------   280

AF466202.2_FG007  GTAATATAATTCCTCTCCAATTCTCCGACTGTTCGCTTGT   316
AF466202.2_FG007B ----------------------------------------   320

AF466202.2_FG007  CGTGCCAATTTGCCTGCCCACGTCTTCGAGCAATTTGTGC   356
AF466202.2_FG007B ----------------------------------------   360

AF466202.2_FG007  ACACGCTGCAACTCATCTTAAGGGTTATTAGATTGAGGGA   396
AF466202.2_FG007B ----------------------------------------   400

AF466202.2_FG007  CGTAGATTGCATTTACATGCTTGAGATTGTTATGGATGGG   436
AF466202.2_FG007B ----------------------------------------   440

AF466202.2_FG007  CTGATGGAGCATATGCATTGGAAGGAAGAAAGAGCTTGGC   476
AF466202.2_FG007B ----------------------------------------   480

AF466202.2_FG007  CTTACAAACTTTTGGATGCAACTTATGTTGCAGAGAATAA   516
AF466202.2_FG007B ----------------------------------------   520

AF466202.2_FG007  GCTATGGTACACACCTGAGTGCACTGGTAATGGGTCGAAT   556
AF466202.2_FG007B ----------------------------------------   560

AF466202.2_FG007  GGGCAGGCCGGCCCAAGCCCAAGGGCTTTTCACATAGCTG   596
AF466202.2_FG007B ----------------------------------------   600

AF466202.2_FG007  TTGTCATTGACTGCAACATGTTCATCTTTGGTGGCCGATC   636
AF466202.2_FG007B ----------------------------------------   640

AF466202.2_FG007  TGGGGCAAGCGGTATGCAGTCCACCAGATCTGTTGGTCAA   676
AF466202.2_FG007B ----------------------------------------   680

AF466202.2_FG007  TCGAGCGTCAGGATTGTTAGCTTCTTAGTTTGGCGAACTA   716
AF466202.2_FG007B ----------------------------------------   720

AF466202.2_FG007  GGGGATGGGTTGGTCTGGGTGGATCTTGGGTTGCGGGTCT   756
AF466202.2_FG007B ----------------------------------------   760

AF466202.2_FG007  ACGGCCGAGCCGCATGATCTTTGATGCAATTTCGTCTGAG   796
AF466202.2_FG007B ----------------------------------------   800

AF466202.2_FG007  GATGCTGCTTGCATGAGTACACAGATTGTGCTTTTGTTTC   836
AF466202.2_FG007B ----------------------------------------   840
```

```
AF466202.2_FG007  TGAATCCACCTCACACAGTCCAATTTCTTGCAGTGGAAAA   876
AF466202.2_FG007B ----------------------------------------   880

AF466202.2_FG007  GAAGTCCCATCCATCCATTTGCATTTCATATTTGGTCGTA   916
AF466202.2_FG007B ----------------------------------------   920

AF466202.2_FG007  TGCTAAGCTATTAGCTATATCTTTGTTTGTCTATTATCTT   956
AF466202.2_FG007B ----------------------------------------   960

AF466202.2_FG007  TACCAGATTAGAGCAATTAGTATATCATTACCTTCTCTAT   996
AF466202.2_FG007B ----------------------------------------  1000

AF466202.2_FG007  TCACCCTTTTTTTGTATTCAGTTATGTATCAAATGCAGTT  1036
AF466202.2_FG007B ----------------------------------------  1040

AF466202.2_FG007  TTGATATTTGATAGGAGAATGGTAGACCATTGCGAGGGCA  1076
AF466202.2_FG007B ----------------------------------------  1080

AF466202.2_FG007  GTGGAGGAGTTGGGCGACGTGGTCAGACGAAGGCAGGGCA  1116
AF466202.2_FG007B ----------------------------------------  1120

AF466202.2_FG007  AAGCCACGTCCAGTAGCCACGAGGCTGCATCGCGAGGGCG  1156
AF466202.2_FG007B ----------------------------------------  1160

AF466202.2_FG007  GTGGAGGAGCTGGGCGACGAGGTCAGACTAAGGCGAGGTG  1196
AF466202.2_FG007B ----------------------------------------  1200

AF466202.2_FG007  AAGAATCACTGTTGGGCTGCATCAGTCAGCACCCTCTAAG  1236
AF466202.2_FG007B ----------------------------------------  1240

AF466202.2_FG007  GGAGGAGGATGCATATGATGCGTCAGCTTAAGAGCATCGC  1276
AF466202.2_FG007B ----------------------------------------  1280

AF466202.2_FG007  CTTCGGCTGCTCGTCAGTCTCCAATCCGGTGAGAGCTCCA  1316
AF466202.2_FG007B ----------------------------------------  1320

AF466202.2_FG007  ATTATCAGTCCATTCCACTGCTATTGGTGGTTTGGGTACC  1356
AF466202.2_FG007B ----------------------------------------  1360

AF466202.2_FG007  TGCATTGGGTGTTTTTTAGATAGCAAACATTTTTTTTAGA  1396
AF466202.2_FG007B ----------------------------------------  1400

AF466202.2_FG007  TTGTCATAGGACTTGGACTTGTGAAAGCGCTAAGAAAAGA  1436
AF466202.2_FG007B ----------------------------------------  1440

AF466202.2_FG007  AGTAGATTCGACTGAAACTTTTCTAATTTATTTAGTGCTC  1476
AF466202.2_FG007B ----------------------------------------  1480

AF466202.2_FG007  AATAATATTCCATACTGACGTTACATATTGTCTTTATTAA  1516
AF466202.2_FG007B ----------------------------------------  1520

AF466202.2_FG007  ATAAGAGGCGGTTCATTATCTTGTCTTTATTAAATAAGAG  1556
AF466202.2_FG007B ----------------------------------------  1560

AF466202.2_FG007  GCGGTTCAATAAAATTTTCAGTTACATTCTACTGTCTTGC  1596
AF466202.2_FG007B ----------------------------------------  1600
```

```
AF466202.2_FG007  TAATATCTATTGTCTTTATTAATTACAAGATTGGCGGACA  1636
AF466202.2_FG007B ----------------------------------------  1640

AF466202.2_FG007  AAAAGGATTAACCAACCTTACTGGTATAATATGTTATTGA  1676
AF466202.2_FG007B ----------------------------------------  1680

AF466202.2_FG007  AAATTCCTATTTATGAAAGTAAGGTTGTTTAATATTATTA  1716
AF466202.2_FG007B ----------------------------------------  1720

AF466202.2_FG007  TTTCAGTAAATTCTATTTCAGTTCGAGAACCTTTTTGCAC  1756
AF466202.2_FG007B ----------------------------------------  1760

AF466202.2_FG007  ACCAACTGTTTTATTTATGTTTATATGTTATATAATAATA  1796
AF466202.2_FG007B ----------------------------------------  1800

AF466202.2_FG007  GAGTAGTAGAGATTTTGGTACTACCTAATTAGATGTTGAT  1836
AF466202.2_FG007B ----------------------------------------  1840

AF466202.2_FG007  GTTTACTCTATTTCAGTTCTAGAAATATTTTGCTGATCAG  1876
AF466202.2_FG007B ----------------------------------------  1880

AF466202.2_FG007  TAAAAAATCAAACATGATACTTTTCCATAAAAGGTTGTTT  1916
AF466202.2_FG007B ----------------------------------------  1920

AF466202.2_FG007  TCCTGTTCTATTTGTTGTACGTAATACATATATTTTGCCC  1956
AF466202.2_FG007B ----------------------------------------  1960

AF466202.2_FG007  AAGGTTAGCCCCCCTGGTCTCGCGCGCCTGCGGCGGAGAT  1996
AF466202.2_FG007B ----------------------------------------  2000

AF466202.2_FG007  CGGTCCAAGTTTAGTCTCTAGCGTTTGGCTGATTAGTCGG  2036
AF466202.2_FG007B ----------------------------------------  2040

AF466202.2_FG007  TTGTGGTGCTCTTCTGCTTGTTTGCTTCTAGGACAAATTG  2076
AF466202.2_FG007B ----------------------------------------  2080

AF466202.2_FG007  CTGGACATGACCAGCGTTTCGCGCCTGTTCCCGATCACCG  2116
AF466202.2_FG007B ----------------------------------------  2120

AF466202.2_FG007  ACCGGCTGGGGTTGCTCGCCACGGGGATCGCAGGTGACCT  2156
AF466202.2_FG007B ----------------------------------------  2160

AF466202.2_FG007  CAGCAGTGCTTGCTTCTCATGTCATTTCTGCCAGAATGCT  2196
AF466202.2_FG007B ----------------------------------------  2200

AF466202.2_FG007  TTGCCGAATGTACCTAAATATATGAAGTCCTGGATTAGTG  2236
AF466202.2_FG007B ----------------------------------------  2240

AF466202.2_FG007  TCCTATCTTTGTTTTCTACATCATCCTCAACCTTTTCTTC  2276
AF466202.2_FG007B ----------------------------------------  2280

AF466202.2_FG007  CCTAATAATGTCATTTATTTTCTAAAATACATGATCTAGA  2316
AF466202.2_FG007B ----------------------------------------  2320

AF466202.2_FG007  GTTTAATGGTTGGAGCTCAATTTGTTTTTAGTGCCACAAA  2356
AF466202.2_FG007B ----------------------------------------  2360
```

```
AF466202.2_FG007  CACTTTAAATGATAGGGTATTTTAATTTCGGGGCACTATT  2396
AF466202.2_FG007B ----------------------------------------  2400

AF466202.2_FG007  TTGAGGCCCCTCGTTGGAGATGCTCTTACGGTTTTTGGGG  2436
AF466202.2_FG007B ----------------------------------------  2440

AF466202.2_FG007  TGATGATGCCGGGGAGTTTTGGGGTAATGGTGTAATACCC  2476
AF466202.2_FG007B ----------------------------------------  2480

AF466202.2_FG007  ATGGCATAGCATGAGGGCGTTTGAAAATTTAAACTACTAG  2516
AF466202.2_FG007B ----------------------------------------  2520

AF466202.2_FG007  GTGTATCCTCAAAATTCTTCTATGAACCTTTTTAAATTGA  2556
AF466202.2_FG007B ----------------------------------------  2560

AF466202.2_FG007  GTTCTTTAGACTTGCATTTTGGTTGGTTGACACACCAACA  2596
AF466202.2_FG007B ----------------------------------------  2600

AF466202.2_FG007  TGCTGTAATCATATTCAATTTGATAATCTGAAACCTTTGG  2636
AF466202.2_FG007B ----------------------------------------  2640

AF466202.2_FG007  TCATACACAGCAGAGGGTCAATTCGACTGTCATCATTAAC  2676
AF466202.2_FG007B ----------------------------------------  2680

AF466202.2_FG007  GCACACATATAATGCATTAATGCATCACATTGTAGAAGTT  2716
AF466202.2_FG007B ----------------------------------------  2720

AF466202.2_FG007  GGTCTAAATTCTAATGATCTGTTTGGTTTTACTGATTGAT  2756
AF466202.2_FG007B ----------------------------------------  2760

AF466202.2_FG007  GTGAGCTATTTCTGCCTTATGTTCATGAGCATGGAATGCA  2796
AF466202.2_FG007B ----------------------------------------  2800

AF466202.2_FG007  TTATTGCTAGCTAAGCTTGACACCACTTCAAGCAAGGTGG  2836
AF466202.2_FG007B ----------------------------------------  2840

AF466202.2_FG007  CTCTGCCAGTTTATTACTTCTGTTTCTAATCACCATTGCA  2876
AF466202.2_FG007B ----------------------------------------  2880

AF466202.2_FG007  TCTGACCATTGGCTTGGTACCTTGTTAAACATTACACAAT  2916
AF466202.2_FG007B ----------------------------------------  2920

AF466202.2_FG007  TGCCATTAAATTTAACACCTGAAATGGTTTAGAATTTTCT  2956
AF466202.2_FG007B ----------------------------------------  2960

AF466202.2_FG007  GTTCAAATTCGAACCAACTCGAATCACCATTACCAGTACT  2996
AF466202.2_FG007B ----------------------------------------  3000

AF466202.2_FG007  GCCACGTGGAGCACCAAAGATGGTAACATCAAAACATGAC  3036
AF466202.2_FG007B ----------------------------------------  3040

AF466202.2_FG007  ATAATAGTTATTTTTATTTGCATGCTTGTTTTAGAATGCC  3076
AF466202.2_FG007B ----------------------------------------  3080

AF466202.2_FG007  GATAATCATATTTTTCTTCTTCAATCATCATTACAGGTGA  3116
AF466202.2_FG007B ----------------------------------------  3120
```

```
AF466202.2_FG007  GGGGAGATCATTGGCACATGAGGCAAGGAATCAAACTGCA   3156
AF466202.2_FG007B ----------------------------------------   3160

AF466202.2_FG007  GAATTTCGTTTCAATTGGGGATATGAAATGCCTCCTGATG   3196
AF466202.2_FG007B ----------------------------------------   3200

AF466202.2_FG007  TTTTGGCACAATGGTAAAAAATGAGCTCAAGGAAATATAT   3236
AF466202.2_FG007B ----------------------------------------   3240

AF466202.2_FG007  ATTATATCAGGACTTCAGACTGGCAGTGCCTATAATTGCT   3276
AF466202.2_FG007B ----------------------------------------   3280

AF466202.2_FG007  TGATGACATGAAAGCCGCAGATTCAGTTTCGGTATGTTAC   3316
AF466202.2_FG007B ----------------------------------------   3320

AF466202.2_FG007  AATTTTTAGAGCACTATGCTTAGCTACACTACTCAGTACT   3356
AF466202.2_FG007B ----------------------------------------   3360

AF466202.2_FG007  TACATATGAATGGAATACAGACACATGTATGGTGATGCTT   3396
AF466202.2_FG007B ----------------------------------------   3400

AF466202.2_FG007  CTAATTCTCTTTGAAACTGAAACTTCAGATGTGCCTATCT   3436
AF466202.2_FG007B ----------------------------------------   3440

AF466202.2_FG007  AAAGGTTTAGGCGTCCCTATTGGATTCAAAGGCTTTTGTT   3476
AF466202.2_FG007B ----------------------------------------   3480

AF466202.2_FG007  GACAAGGTCTATCCATGTGTGTTTCTAAGTCTAAATTGTA   3516
AF466202.2_FG007B ----------------------------------------   3520

AF466202.2_FG007  GTGTTGTATTATTGCAAAATGAAAAGAACTGTATATTATA   3556
AF466202.2_FG007B ----------------------------------------   3560

AF466202.2_FG007  ATGGAAAATGAACAATACCTTCAGGCCAAAAAATCTTCGA   3596
AF466202.2_FG007B ----------------------t-----------------   3600

AF466202.2_FG007  AAGATCCTAGGTGGTGGAATGAGGCAGGTTGGAGTTCTGT   3636
AF466202.2_FG007B ----------------------------------------   3640

AF466202.2_FG007  GTGCTGGTGCTCATACACTATGGGAAAGCTTGCTGGTGAG   3676
AF466202.2_FG007B ----------------------------------------   3680

AF466202.2_FG007  GATAGAAAGGCTAAAGTTGTGGCAGGTCTATTTTTTTCCC   3716
AF466202.2_FG007B ------------------------------.---------   3719

AF466202.2_FG007  TCTTTTCTAAAAATTTTAGAATTGGTTGTATGCTATTTAA   3756
AF466202.2_FG007B ----------------------------------------   3759

AF466202.2_FG007  GGCAAAATGTTTTTCTATTGTCTAAAAAATGATTTATTTT   3796
AF466202.2_FG007B ----------------------------------------   3799

AF466202.2_FG007  TTCCCAGAATATTTATTTCCTAGCTTTTTATCATACTTAA   3836
AF466202.2_FG007B ----------------------------------------   3839

AF466202.2_FG007  AACAAATCTTTGCTTGTCATTTATTTACCGACAGCAATGT   3876
AF466202.2_FG007B ----------------------------------------   3879
```

```
AF466202.2_FG007  GCCATTGTACTGCAATTTGTGCCATTGTACTACAATTTGG  3916
AF466202.2_FG007B ----------------------------------------  3919

AF466202.2_FG007  TAGCTCAATCAATATTTCAGCAATGTGCCATTGTACTGCA  3956
AF466202.2_FG007B ----------------------------------------  3959

AF466202.2_FG007  ATTTGTTGCCATGACATTTTTAGGTCCCAACCAAAGACTG  3996
AF466202.2_FG007B ----------------------------------------  3999

AF466202.2_FG007  TATCCCTCTTTTGCTGGTTGTTTCATGTGATATCTTTGCT  4036
AF466202.2_FG007B ----------------------------------------  4039

AF466202.2_FG007  TGTCGTTGGATGTTGCCTTTTACATAGCCAAATGTTCCAA  4076
AF466202.2_FG007B ----------------------------------------  4079

AF466202.2_FG007  CTAATTTTCCAACATCTTGTCTACCTTTACCTGTTAGTTG  4116
AF466202.2_FG007B ----------------------------------------  4119

AF466202.2_FG007  TAGCTTGAAACCATGCTGTCTTTGATGTCAATGAGAACTT  4156
AF466202.2_FG007B ----------------------------------------  4159

AF466202.2_FG007  TGTTTGCAAATAGATGTCCCACAAATTCTAACATATACTT  4196
AF466202.2_FG007B ----------------------------------------  4199

AF466202.2_FG007  TGGTGCTATCAAAGGGAGCCACAATAGTTGATATGGCTGT  4236
AF466202.2_FG007B ----------------------------------------  4239

AF466202.2_FG007  AATCGTCACACCAGCCAATGATTATGAGCTACCAGCTTCT  4276
AF466202.2_FG007B ----------------------------------------  4279

AF466202.2_FG007  GTTTTAGCTGCTCTTGAGGTATGATCAGAACCATACTATT  4316
AF466202.2_FG007B ----------------------------------------  4319

AF466202.2_FG007  TACATGTTACAACATGGTATTGCTATAGTTGTTGTACAGC  4356
AF466202.2_FG007B ----------------------------------------  4359

AF466202.2_FG007  GTTGTAACATTATTTTCATTTCTAGCCTAAAGACACAAAA  4396
AF466202.2_FG007B ----------------------------------------  4399

AF466202.2_FG007  CCTTATGCCCTTCAAAAGGCCGAGGACATTGTTGGGACCA  4436
AF466202.2_FG007B ----------------------------------------  4439

AF466202.2_FG007  TGCTGGCAAAGGGATTTATCCTTGGTAGAGATGCACTGGA  4476
AF466202.2_FG007B ----------------------------------------  4479

AF466202.2_FG007  CAAAGCAAAAGCTTTGGATGAAAAGCACCAGCTCACGTCA  4516
AF466202.2_FG007B ----------------------------------------  4519

AF466202.2_FG007  ACTGCCACAGCTAGAGTGTCCTCCTTTGACAAGAGAATAG  4556
AF466202.2_FG007B ----------------------------------------  4559

AF466202.2_FG007  GCCTAAGTGAGAAGATCAGTGTTGGCACTTCAGTTGTAAA  4596
AF466202.2_FG007B ----------------------------------------  4599

AF466202.2_FG007  TGATAAAGTGAAGGAAATGGATCAGAAATACCAAGTCTCT  4636
AF466202.2_FG007B ----------------------------------------  4639
```

```
AF466202.2_FG007  GAGAAGACAAAGTCAGCGCTGGCAGCTGCTGAACAGAGTG   4676
AF466202.2_FG007B ----------------------------------------   4679

AF466202.2_FG007  TCTTGACCGTTGGATCTGCTATCATGAAGAACATGTATGT   4716
AF466202.2_FG007B ----------------------------------------   4719

AF466202.2_FG007  TCTCACCGGAGCAGCATGGGTAACCGGTGCTTTCAGCAAG   4756
AF466202.2_FG007B ----------------------------------------   4759

AF466202.2_FG007  GTCACCAGTGCTGCCAACGATGTTGGTGCAAAGGCTAAGG   4796
AF466202.2_FG007B ----------------------------------------   4799

AF466202.2_FG007  AGAAAATAGCAGTCGAGCAGGAGGACAAGAATGCCGAGGG   4836
AF466202.2_FG007B ----------------------------------------   4839

AF466202.2_FG007  TGGGCCTGGACAAGCAAACATCTCAGAGATCCCTGCAGCA   4876
AF466202.2_FG007B ----------------------------------------   4879

AF466202.2_FG007  CACAGGGAGTTGGACTGCGAATTCGCAAACATACATGTCT   4916
AF466202.2_FG007B ----------------------------------------   4919

AF466202.2_FG007  CTGAAACCTCTGAAGATGTTCCCATGTCTTCCGTGACTGT   4956
AF466202.2_FG007B ----------------------------------------   4959

AF466202.2_FG007  CCCGCTGTTACAGATGAGGAGCCCAGCATGGCCACTCCAC   4996
AF466202.2_FG007B ----------------------------------------   4999

AF466202.2_FG007  CAGCTGAAGTTTCTAAAAAACCAGAAGCTGCACAGGGATT   5036
AF466202.2_FG007B ------------------------------------....   5035

AF466202.2_FG007  AA                                     5038
AF466202.2_FG007B ..                                     5035
```

Predicted coding sequence sequence of AF466202.2_FG007B base on

AF466202.2_FG007

>AF466202.2_FG007B_coding_sequence
CAAGTCCAAGGTGGTCCGTCTTCGGCGGATTCACCGACAAGCGCTTCCTCGCTGATGTTTCCGTCTACGACGTAG
AGAATAAGCTATGGTACACACCTGAGTGCACTGGTAATGGGTCGAATGGGCAGGCCGGCCCAAGCCCAAGGGC
TTTTCACATAGCTGTTGTCATTGACTGCAACATGTTCATCTTTGGTGGCCGATCTGGGGCAAGCGGTGAGGGGAG
ATCATTGGCACATGAGGCAAGGAATCAAACTGCAGAATTTCGTTTCAATTGGGGATATGAAATGCCTCCTGATGT
TTTGGCACAATGGCCAAAAAATCTTCGAAAGATCCTAGGTGGTGGAATGAGGCAGGTTGGAGTTCTGTGTGCTG
GTGCTCATACACTATGGGAAAGCTTGCTGGGAGCCACAATAGTTGATATGGCTGTAATCGTCACACCAGCCAATG
ATTATGAGCTACCAGCTTCTGTTTTAGCTGCTCTTGAGCCTAAAGACACAAAACCTTATGCCCTTCAAAAGGCCGA
GGACATTGTTGGGACCATGCTGGCAAAGGGATTTATCCTTGGTAGAGATGCACTGGACAAAGCAAAAGCTTTGG
ATGAAAAGCACCAGCTCACGTCAACTGCCACAGCTAGAGTGTCCTCCTTTGACAAGAGAATAGGCCTAAGTGAG
AAGATCAGTGTTGGCACTTCAGTTGTAAATGATAAAGTGAAGGAAATGGATCAGAAATACCAAGTCTCTGAGAA
GACAAAGTCAGCGCTGGCAGCTGCTGAACAGAGTGTCTTGACCGTTGGATCTGCTATCATGAAGAACATGTATGT
TCTCACCGGAGCAGCATGGGTAACCGGTGCTTTCAGCAAGGTCACCAGTGCTGCCAACGATGTTGGTGCAAAG
GCTAAGGAGAAAATAGCAGTCGAGCAGGAGGACAAGAATGCCGAGGGTGGGCCTGGACAAGCAAACATCTCA
GAGATCCCTGCAGCACACAGGGAGTTGGACTGCGAATTCGCAAACATACATGTCTCTGAAACCTCTGAAGATGT

TCCCATGTCTTCCGTGACTGTCCCGCTGTTACAGATGAGGAGCCCAGCATGGCCACTCCACCAGCTGAAGTTTCT
AAAAAACCAGAAGCTGCACAGGGATTAA

Multiple_Sequence_Alignment GRMZM2G356653 and Grmzm2g356653B

```
GRMZM2G356653  GTTTTCCCTGGTAAAAGTGCATTTCCTGTGGTTTGAGTTG   40
Grmzm2g356653B ----------------------------------------   40

GRMZM2G356653  AACTTATTCCTGATAATTGCTTCTTTTCTTATTAATGATT   80
Grmzm2g356653B ----------------------------------------   80

GRMZM2G356653  TTTTTCTTGTCAGGAACATCTCTGGTTATCTGGAGTTGGC   120
Grmzm2g356653B ----------------------------------------   120

GRMZM2G356653  GGTGGCCTTTTCATTCATGGTTGCCTGCACTCATCCGGGT   160
Grmzm2g356653B ----------------------------------------   160

GRMZM2G356653  TGTAGCAATATCGGTCTCCAGGTGTGTGGGGGAGGAAATG   200
Grmzm2g356653B ------------------------------------g---   200

GRMZM2G356653  GATGTTACTATTAGGGAAGTGGTGTTTAGCAAGCATGTTA   240
Grmzm2g356653B ----------------------------------------   240

GRMZM2G356653  ATTTCAGGTATATTTTCAACAGTGACATGTTATACAATGT   280
Grmzm2g356653B ----------------------------------------   280

GRMZM2G356653  ATAAGTTCTAGCAACTACCACTGCAAATGAAGATTTTTTT   320
Grmzm2g356653B ----------------------------------------   320

GRMZM2G356653  AATTCCAGTTTGGTATGCACCATTATAGATGTGCTCACTC   360
Grmzm2g356653B ---------------------------a------------   360

GRMZM2G356653  TAGGGGTAGTTGCAATTCTCAAATAGTTTGCTTAAAATGT   400
Grmzm2g356653B ----------------------------------------   400

GRMZM2G356653  GTGGCCAGGGCTAGGTCACTCTCAAATAGTATTTTACATT   440
Grmzm2g356653B ----------------------------------------   440

GRMZM2G356653  AACTCTAGCTGCAAGAATGGTACTTTATACATCCATTAGT   480
Grmzm2g356653B ----------------------------------------   480

GRMZM2G356653  TCGCCTAAAATGTGTGGGAATTATGTAGAGATGTGCCAAT   520
Grmzm2g356653B ----------------------------------------   520

GRMZM2G356653  ATATAAAATAAACTTTTAAGCTCAAACAACCCCTTTGATT   560
Grmzm2g356653B ----------------------------------------   560

GRMZM2G356653  GATGTGCCACTGCTGATGCAGTAGCGTTATTGTGCCAAAT   600
Grmzm2g356653B ----------------------------------------   600

GRMZM2G356653  GGATACATGAGGATTAGGATGCTAATCCAAAGTAGGTGCA   640
Grmzm2g356653B ----------------------------------------   640

GRMZM2G356653  CCCCATGATTAAGCTCCTATGTCCAGATATGTATATTACC   680
Grmzm2g356653B ----------------------------------------   680
```

```
GRMZM2G356653  TATCAACAAATCTTGGCATCCAGGTTTAAACTCCTCCCGC   720
Grmzm2g356653B ----------------------------------------   720

GRMZM2G356653  TGCTCGCCGATGTAAAGGTACGCACCTGCTGTCGAACTAT   760
Grmzm2g356653B ----------------------------------------   760

GRMZM2G356653  GGCTCTAACTGATATAGCTTTTAGGCCCAATAACATCTAC   800
Grmzm2g356653B ----------------------------------------   800

GRMZM2G356653  CACTGCTTATCATGCCATTAAATTGTTTGATGAAATACCA   840
Grmzm2g356653B ----------------------------------------   840

GRMZM2G356653  ATATAATATAGTAATGTGGCATATATCAGTTCAGTTTGAG   880
Grmzm2g356653B ----------------------------------------   880

GRMZM2G356653  TTGTAGTTGTAACTTGAGAACATATGTTTTGTGTCTAATA   920
Grmzm2g356653B -------------------------a--------------   920

GRMZM2G356653  AGTTATGAATTTTTGTTGTTGTTCTTCCCTTCTGCAAAAC   960
Grmzm2g356653B ----------------------------------------   960

GRMZM2G356653  TTCAGATCTCCTACCTCTGCGATTCTAAAAAGAAGATTCA  1000
Grmzm2g356653B ----------------------------------------  1000

GRMZM2G356653  GGTCCCATTTGCCATAGTGGATCCGTTTCCACTGTAGCTA  1040
Grmzm2g356653B ----------------------------------------  1040

GRMZM2G356653  TTGTTTTTGTTTGTTCTCTGGCAAAAAAAACTTAGCAAAT  1080
Grmzm2g356653B ---c------------------------------------  1080

GRMZM2G356653  GCTTATAGGAATAATGAGTTATCTTGGAAGATGATTACTT  1120
Grmzm2g356653B ----------------------------------------  1120

GRMZM2G356653  AGATAGGTGCATGTTTATTTCAGAGCTTATTTCAACCATT  1160
Grmzm2g356653B ----------------------------------------  1160

GRMZM2G356653  TAACTATGTGTCCAACCATTTTGGGCTGGTAACTTCTTTA  1200
Grmzm2g356653B ----------------------------------------  1200

GRMZM2G356653  TTGAACCAGTATTTTGTCATTAAATTTACTCTACTTCAAA  1240
Grmzm2g356653B ----------------------------------------  1240

GRMZM2G356653  AAACCTGACCTGGAAAAAACTACAGAGACTGAACAAGGGG  1280
Grmzm2g356653B ----------------------------------------  1280

GRMZM2G356653  TGGCGCAGGCTATTCTTCGTGGAAATATCGATTTTAAGCG  1320
Grmzm2g356653B ----------------------------------------  1320

GRMZM2G356653  CGAACCCGCCGCCGCCAGCCCGCCCACCACGCTCTTCGCT  1360
Grmzm2g356653B ----------------------------------------  1360

GRMZM2G356653  AGCTCGCCCACCGCGCCGCCGCCAGCCAGCCCATCGCGCT  1400
Grmzm2g356653B ----------------------------------------  1400

GRMZM2G356653  ACAGACGCGCTGCCGGTACCCTCTCCCTCTCCGCCCGGAA  1440
Grmzm2g356653B -------------------------------t--------  1440
```

```
GRMZM2G356653  GTTCTTGGGTAACCATCTCACTACAGGTAACCATCTCCCT  1480
Grmzm2g356653B ----------------------------------------  1480

GRMZM2G356653  CTCTGAGATAAATGTTTAAACTTTTATTTAGGGTCCATAC  1520
Grmzm2g356653B ----------------------------------------  1520

GRMZM2G356653  TTTTAAGTGTTCGATCCCTTCAAATGCTACACTTCCAAGC  1560
Grmzm2g356653B ----------------------------------------  1560

GRMZM2G356653  AATGCATAATGTATTAATGTGGGGTTGTGGGCGTTGGAAT  1600
Grmzm2g356653B ----------------------------------------  1600

GRMZM2G356653  GTGCATGTTGACTAGCTTTGATTAGCTTGTCGATATACAT  1640
Grmzm2g356653B ----------------------------------------  1640

GRMZM2G356653  ACTTAGCAATCTATTTTTTCTTCTCCTTTCAAATGCTATG  1680
Grmzm2g356653B ----------------------------------------  1680

GRMZM2G356653  CTATTGCTACTGCTGTTGGAGTGTACCATTTAACTTGATA  1720
Grmzm2g356653B ----------------------------------------  1720

GRMZM2G356653  TATGATTTAATAGATGCTTTGTGTGCATAAATCGTCTACA  1760
Grmzm2g356653B ----------------------------------------  1760

GRMZM2G356653  GTTACCATTTGGACTTTTTTAGCCATGTTTGAGAGATGTG  1800
Grmzm2g356653B ----------------------------------------  1800

GRMZM2G356653  ACGGAGTTTGGATTTCACTTAAAAATGCAGGTCCAGTTGG  1840
Grmzm2g356653B ----------------------------------------  1840

GRMZM2G356653  AATCGAATACTCTGTTTGCTGGCCTATGCTAATGAGTAAT  1880
Grmzm2g356653B ----------------------------------------  1880

GRMZM2G356653  CTATTTCAGATTTGGATATGTGCAAGACACAATGGTTGCT  1920
Grmzm2g356653B ----------------------------------------  1920

GRMZM2G356653  CAACGAACCAGGTGCCTAGCTCTCTTGGAATTTTGGTGTA  1960
Grmzm2g356653B ----------------------------------------  1960

GRMZM2G356653  ATTTGAAGTCTACTGCTACTGTATTAGTCTCAGTTCTCTA  2000
Grmzm2g356653B ----------------------------------------  2000

GRMZM2G356653  TTGTTTGATTCCGCTTTTCTTCTCACGCGTGCAGCACCAG  2040
Grmzm2g356653B ----------------------------------------  2040

GRMZM2G356653  GTATAAGTGTGTGTCTCCGTCGGAGGAGAACATGCGGTACTC  2080
Grmzm2g356653B ----------------------------------------  2080

GRMZM2G356653  AACGTCATAATCCTTGGCCATGCGCATTCACTTTGCAGTC  2120
Grmzm2g356653B ----------------------------------------  2120

GRMZM2G356653  AATTCCTCATTTATGATTGGCTTCACATGACATTACTTTT  2160
Grmzm2g356653B ----------------------------------------  2160

GRMZM2G356653  TGTATGGGTAGTCTTTGTGCTTCTGATGTTGTTTCTAGAA  2200
Grmzm2g356653B ----------------------------------------  2200
```

```
GRMZM2G356653  TTATTAATAGATATATTTATAGAAGGAAAAAGCAAAGGTC   2240
Grmzm2g356653B ----------------------------------------   2240

GRMZM2G356653  AGGGCACTGGTACTATTTCAGAAGGTATATTTGGTTTGTG   2280
Grmzm2g356653B ----------------------------------------   2280

GRMZM2G356653  CAGCTTCATTTGCAATGCATATATTTATTCTTCTTTACTA   2320
Grmzm2g356653B ----------------------------------------   2320

GRMZM2G356653  AAAAGAGACTCATCTTGTTAATTTAGTAATATATTTGTAT   2360
Grmzm2g356653B ----------------------------------------   2360

GRMZM2G356653  GTCCTGGGAAAAGATTGCTAGAGGAAAATTTGCTAAATTC   2400
Grmzm2g356653B ----------------------------------------   2400

GRMZM2G356653  TACAACTACACATAACCTTTGTTTTCTTTTTCATTTCAGC   2440
Grmzm2g356653B ----------------------------------------   2440

GRMZM2G356653  TATTGGTGAGTACTTACCGCTATGATGGACAAGACTTTTA   2480
Grmzm2g356653B ----------------------------------------   2480

GRMZM2G356653  TCATTTCTTTTTTAGCCAGATTGTGGTTAGATGTGAGATT   2520
Grmzm2g356653B ----------------------------------------   2520

GRMZM2G356653  ACTGTAGTTCATAGACCATAGCAGATGCTCGTAAGCAGAG   2560
Grmzm2g356653B ----------------------------------------   2560

GRMZM2G356653  GTTCGCTGCTGTAAATGGATTGATTTCTTGCTTTTCCAGA   2600
Grmzm2g356653B ----------------------------------------   2600

GRMZM2G356653  ACGCATTGGCAAAGATCATGTCGCTGTCTCAGCACAGCAC   2640
Grmzm2g356653B ----------------------------------------   2640

GRMZM2G356653  ACACGGTTTGCATCCATTCCTGTAGTGGACCAAGTGACTG   2680
Grmzm2g356653B ----------------------------------------   2680

GRMZM2G356653  TAGTATGAAGAAGCGCGTGTCATTTTCCTGATTAATTTAG   2720
Grmzm2g356653B ----------------------------------------   2720

GRMZM2G356653  CTGGTTCAACTCTGTTGGGGTCCCAAGTCTTGGCCATAAT   2760
Grmzm2g356653B ----------------------------------------   2760

GRMZM2G356653  TTGATGTAATTAAACTTCTTTAGACAATTGAGTACCTTTC   2800
Grmzm2g356653B ----------------------------------------   2800

GRMZM2G356653  TATGTGGATTTACATATCCCTTTGCCTTATAGTTTGGCAT   2840
Grmzm2g356653B ----------------------------------------   2840

GRMZM2G356653  ATTATTGCACTTCATGTTGACACACTTCTGATAGCATGTT   2880
Grmzm2g356653B ----------------------------------------   2880

GRMZM2G356653  TACCTTGGGATAAGATTTCTAATGTTTGCCAATATTGCCA   2920
Grmzm2g356653B ----------------------------------------   2920

GRMZM2G356653  AGTAAAATAAAATGTCAGTGGCTTCCTTGAACACCCAGAT   2960
Grmzm2g356653B ----------------------------------------   2960
```

```
GRMZM2G356653  TATTTGCTACCTTCCTTTTGCATTACCACTATAATTTTCA   3000
Grmzm2g356653B --------------t-------------------------   3000

GRMZM2G356653  AAGAAGGATGCTCTTTATCTTGTCTCTTTACAGGTTTGTG   3040
Grmzm2g356653B --------------c-------------c-----------   3040

GRMZM2G356653  TGGCGAAAGAAGATAGAGCGTGATGTATCTCAGGGACAGA   3080
Grmzm2g356653B ----------------------------------------   3080

GRMZM2G356653  AGGTGAATATTTCTGTTAAATCTGAGAAGAAGAAACAATA   3120
Grmzm2g356653B ----------------------------------------   3120

GRMZM2G356653  AGAAAGAATGGTACTATGAAACACTATATCTATTTCCATT   3160
Grmzm2g356653B ----------------------------------------   3160

GRMZM2G356653  GTTTATTAGTTGGAACAAAGCATATCATAGTCTTCTTACC   3200
Grmzm2g356653B ----------------------------------------   3200

GRMZM2G356653  TACTTCCAATGAATTAATAAGGTTCATAGCATCATATGTA   3240
Grmzm2g356653B ----------------------------------------   3240

GRMZM2G356653  TTATTTTATTTTCATCTATGCTTTTGTACTGTAGTGCACT   3280
Grmzm2g356653B ----------------------------------------   3280

GRMZM2G356653  GTACGCTTTGCTACCCAGAAAGCTTGTTATTTGGTCTTCT   3320
Grmzm2g356653B ----------------------------------------   3320

GRMZM2G356653  TCAAAAAGAAAATTGAAGATTATGCTTTTCTTGCAGCGCC   3360
Grmzm2g356653B ------------------g---------------------   3360

GRMZM2G356653  TAATCGTTATAGTTGCTCTATCAAATGTAGGCTGAAATAG   3400
Grmzm2g356653B ----------------------------------------   3400

GRMZM2G356653  AAAAGGTTAAGAAAAGAAGAGAAGAAAGAGCTATTGAAAA   3440
Grmzm2g356653B ----------------------------------------   3440

GRMZM2G356653  GACACAGCATGAAGAGGAAATGGTTTGTATTTGACAATCA   3480
Grmzm2g356653B ----------------------------------------   3480

GRMZM2G356653  TAATCTCTTTTATAATAATTTCGTAAGTTTATGTCTACCG   3520
Grmzm2g356653B ----------------------------------------   3520

GRMZM2G356653  TTTATTACATTCAGATATTTTTTGCATTTGGAATTAAATA   3560
Grmzm2g356653B ----------------------------------------   3560

GRMZM2G356653  CATATATTTGCCGTTTTGAAGACATTGCTAGCTAGAGAAA   3600
Grmzm2g356653B ----------------------------------------   3600

GRMZM2G356653  GAGCATGTGCCGAGTTTCAAGACTGGGAGAAGAAAGAGGA   3640
Grmzm2g356653B ----------------------------------------   3640

GRMZM2G356653  AGAGGTCAATATCTTAAAATCTGACGAACACCATTCAATT   3680
Grmzm2g356653B ----------------------------------------   3680

GRMZM2G356653  TGTGTTGGAGTTAATATAGAATTGTTATTTATCTACCATT   3720
Grmzm2g356653B ----------------------------------------   3720
```

```
GRMZM2G356653  TCTCATTTTGGAGTGCAGTTTCATTTTGACCAAAGCAAGG   3760
Grmzm2g356653B ----------------------------------------   3760

GRMZM2G356653  TCAGGTCTGAGATCAGGTTGCGGGAGGGGCGTACAAAGCC   3800
Grmzm2g356653B ----------------------------------------   3800

GRMZM2G356653  TATTGATGTTCTCCAAAAGAATCTTAATTTCTTAGATGAA   3840
Grmzm2g356653B --------------------------------c-------   3840

GRMZM2G356653  TTTGACATTGAGTTAAATGAACCCTATCTGGTGTTCAAGG   3880
Grmzm2g356653B ----------------------------------------   3880

GRMZM2G356653  TGAGCATCATTTGCTCATCCTTATCTAAGTGTAAGTCGAC   3920
Grmzm2g356653B ----------------------------------------   3920

GRMZM2G356653  TGAAAAAAGCTTTGTCCCTGCAGCACCCTACCTGTGTAAT   3960
Grmzm2g356653B ----------------------------------------   3960

GRMZM2G356653  TTTCTGTATGTTTTTTGAAGCCAGCTGTTGTATATTCTGA   4000
Grmzm2g356653B ----------------------------------------   4000

GRMZM2G356653  CACCATCGCACAGTACATCAAATGCATTTTATAACTTAGC   4040
Grmzm2g356653B ----------------------------------------   4040

GRMZM2G356653  AGTTCCTGAAATATGTGTTTTTTGCAATAAACTGGAGGAT   4080
Grmzm2g356653B ----------------------------------------   4080

GRMZM2G356653  GAGATGCACGAGGATGAAGGTTTACCTCCTTTTCTGATTA   4120
Grmzm2g356653B ----------------------------------------   4120

GRMZM2G356653  CTCAATCAACTATGTTTCCAAGATACTGAAGTGATTTCTG   4160
Grmzm2g356653B ----------------------------------------   4160

GRMZM2G356653  ATATTATGTTTTATTTGTAGATGATAAACGATATTCATCT   4200
Grmzm2g356653B ----------------------------------------   4200

GRMZM2G356653  GAGCCTATACCTCAACAGACAGATAACCAATTTGAAGAGG   4240
Grmzm2g356653B ----------------------------------------   4240

GRMZM2G356653  AAGATGGATCCTTTTCTCCACAGCTAATGCATGGCAACGA   4280
Grmzm2g356653B ----------------------------------------   4280

GRMZM2G356653  AGATGAAGATGCAATTGATCCTGAAGAAGACAAAGATGAG   4320
Grmzm2g356653B ----------------------------------------   4320

GRMZM2G356653  CTGGTAGAGTTCTATGTTAGGGCAAAAAGTTCTACTGAGA   4360
Grmzm2g356653B ----------------------------------------   4360

GRMZM2G356653  TTTTCTTTCATTTAGACAAACTTTCAAGCTTGTGCAGGCC   4400
Grmzm2g356653B ----------------------------------------   4400

GRMZM2G356653  TCCCTGTGTGTCTTACCCTAAGAGAGGACATTGA        4434
Grmzm2g356653B ----------------------------------        4434
```

Predicted coding sequence and protein sequence of GRMZM2G356653B base on GRMZM2G356653

>GRMZM2G356653B_coding_sequence
ATGATTTTTTTCTTGTCAGGAACATCTCTGGTTATCTGGAGTTGGCGGTGGCCTTTTCATTCATGGTTGCCTGCAC
TCATCCGGGTTGTAGCAATATCGGTCTCCAGTATTTTGTCATTAAATTTACTCTACTTCAAAAAACCTGACCTGGAA
AAAACTACAGAGACTGAACAAGGGGTGGCGCAGGCTATTCTTCGTGGAAATATCGATTTTAAGCGCGAACCCGC
CGCCGCCAGCCCGCCCACCACGCTCTTCGCTAGCTCGCCCACCGCGCCGCCGCCAGCCAGCCCATCGCGCTACA
GACGCGCTGCCGGTACCCTCTCCCTCTCTGCCCGGAAGTTCTTGGGTAACCATCTCACTACAGCACCAGGTATAA
GTGTGTCTCCGTCGGAGGAGAACATGCGAATTATTAATAGATATATTTATAGAAGGAAAAAGCAAAGGTCAGGGC
ACTGGTACTATTTCAGAAGCTATTGGTTTGTGTGGCGAAAGAAGATAGAGCGTGATGTATCTCAGGGACAGAAG
GCTGAAATAGAAAAGGTTAAGAAAAGAAGAGAAGAAAGAGCTATTGAAAAGACACAGCATGAAGAGGAAATG
ACATTGCTAGCTAGAGAAAGAGCATGTGCCGAGTTTCAAGACTGGGAGAAGAAAGAGGAAGAGTTTCATTTTG
ACCAAAGCAAGGTCAGGTCTGAGATCAGGTTGCGGGAGGGGCGTACAAAGCCTATTGATGTTCTCCAAAAGAA
TCTTAATTTCTCAGATGAATTTGACATTGAGTTAAATGAACCCTATCTGGTGTTCAAGGTGAGCATCATTTGCTCAT
CCTTATCTAAGTATGATAAACGATATTCATCTGAGCCTATACCTCAACAGACAGATAACCAATTTGAAGAGGAAGAT
GGATCCTTTTCTCCACAGCTAATGCATGGCAACGAAGATGAAGATGCAATTGATCCTGAAGAAGACAAAGATGA
GCTGGTAGAGTTCTATGTTAGGGCAAAAAGTTCTACTGAGATTTTCTTTCATTTAGACAAACTTTCAAGCTTGTGC
AGGCCTCCCTGTGTGTCTTACCCTAAGAGAGGACATTGA
> GRMZM2G356653B_protein_sequence
MIFFLSGTSLVIWSWRWPFHSWLPALIRVVAISVSSILSLNLLYFKKPDLEKTTETEQGVAQAILRGNIDFKREPAAASP
PTTLFASSPTAPPPASPSRYRRAAGTLSLSARKFLGNHLTTAPGISVSPSEENMRIINRYIYRRKKQRSGHWYYFRSYWF
VWRKKIERDVSQGQKAEIEKVKKRREERAIEKTQHEEEMTLLARERACAEFQDWEKKEEEFHFDQSKVRSEIRLREG
RTKPIDVLQKNLNFSDEFDIELNEPYLVFKVSIICSSLSKYDKRYSSEPIPQQTDNQFEEEDGSFSPQLMHGNEDEDAID
PEEDKDELVEFYVRAKSSTEIFFHLDKLSSLCRPPCVSYPKRGH*
