# Supplementary material for: B chromosome contains active genes and impacts the transcription of A chromosomes in maize (Zea mays L.)
Source: BMC Plant Biol. 2016 Apr 16;16:88. doi: 10.1186/s12870-016-0775-7 (PMC4833949; doi:10.1186/s12870-016-0775-7)
Supplement: Additional file 13: — Sequencing analysis of B-chromosome located LTR fragments. (DOCX 18 kb) [file 12870_2016_775_MOESM13_ESM.docx]

**Sequencing analysis of B-chromosome located LTR fragments**

1. Sequences of comp75688, comp75688_c6_seq20 is assembled with Trinity, comp75688_1900 is the longer sequence amplified with comp75688-1F/1R. Using the primer comp75688_Full_1F/1R, we sequenced the full length of comp75688 with B73+B (a5_12 and a5_13) and Starter+B (a4_1, a4_2 and a4_4).

>comp75688_c6_seq20 GGAAGCCAAGCGCCCACGTCTTCTTCCTCGCGGTGTCCAGCCTCAACTCAGATTCCCGGCGTCCTCTCCATGGCTCCCTACGCCCTCGTCTTCCCCCTGGTGCCGCGCGGGCGCGCCCCTGGAACCTCCTCGCTGCACGCAGCTGCTTCCCCCTCACTCCCCAGCCGATTTCTCTGCGCGCCCAGCCCAGCCACTCGCCATGGCTCCTCCCTGCGCGCGTCGAAGTTCCTCTGCTCGTCCCATCTCCCCAAAGCTTCTTCCTCCCTGCTCGGCGCTTCCTATTCCCAGCGCTCGACCGCCCCAGCTCGCGCCCAATCCTCCTGCTTGGATTTCGTCCAGATCGAGCTGCTCCCATGGCGATTTCCCCTCGGATTCCTCCCTGCTCGTGCCCCATCGCCATGAACGTGCGCGAGTTTCCACTGGTGGTCGTCGCGCTGGACACCTCCCCTCCGGTCTCCTCTCTCAAGCGCTGCTTCTTCTTCCTGTTGCCGCGCGCCCAGCCGCCTTTCCTTTTCCTCTCGACGCCCGGCCGAGCTCCTCCCTCGCCCATGGCCGCGGTTCTCCAAGTTCCCCTGCGCGCGCGGTCCTCTGTCCCTGCTCGCCCTCTGCGCGTGCTTCTCCCTGCTCCTATGCGCGCGCGGTTCTCTGCTCCCCCGCGCGCTCGTTCTGTTGCTTTTTCCAGCACGCCGCGCGCAGCTCCCTCTAGCTCTGACCATGCCGCGTCGCGTTCCCGTTCCCAGCCATGGTGGTCGATCCCTGCTGCCCAGCTCCTTCGGCACCCAGCCAGGCCGTCCTGGATATTGCGCGCTCCTCCCGGTTGCCATCGCTCGCCGTCGCCTCCTCGTGCTGGCCGACTAGTCTGTCGTGGTCGCACGCAGTTCCCTGCTCGGCCAGGATGGTCGCGTCGTCGATCCCGCCCGGACCTGCAGCCCCAACACACTGTTTCTCTGCTCGGTGCCAAGTATTCTACTACGTCACACCGTGTCCACAGTAAGCCGTGAAGACGCTACTATGCTCGCTGCTCGCCACCTGCCAACATAATGCAAGAACCGGCTATCGATCTTGTCTCTACCATCGCCTTCGCTTTACATCTTTGCTCCCGTTGTCGCGGAGACCCTGCCAGCCCTGCACGTCGTCCACGCTTTGTTGTGTTCGCGTGGAGGGGAATTCTGGGAAATTGGGTGAAGAAACCCGCATGGCGTTTACCGGGTGCTCGACAGATAGCTCATATCGGAGATCGTCGTCGTTCCGCGTGTCAGCAAGAAATTTCAAAAATCGGGTGAAGACGAAGCTAGCAGCGTGGTGTTCACCAAGTGCGCGACAAAAGCTCGGACATCCTGCGCGACTCAAGTTCGATTCGTCCGAATTATTCAATCGAAGAAAAGATCTTCAACAATTGATTACCGAAGAAAAGAAGGCCCCTAGATGACGTCAACCGGAACCCGTAGTCGTGATTGCGACTATCGCGTCGAATTAGTCAGGGTAAGTTGATTCATATTCCCGTTAACTGGGTCTATAAATAGAGTGAATAGAATTATTGGTCTCAATAAATGTTATTAATTTAGCACGTGTATATTGTGATGATCGATTGAGATAAATCGGTAAATCAAGTAATTAAGATACATAGTTTAAAGAGTTAATAAAAAGCTAGGAATAAAAATAGATTTTCTAGTGTAATATGTCAATTTGGTTAATGGATGTAGTCGATGCCCTGCTGTGATATCCATGGTGCTTAATGCTTGGTTTTAAGTGTTTCATGCTTTTTAGCGTAGATTATCTATATTATACTCGTTATACATGTGCATCATGCATCTCATGAGGTACGATAAATAATCACGTGATGCGGAAGAAGAGCCAAGTCGACCCCAAGCGCGGGCAAATCCGCAGGATAATGCTGATGGACGAACCTGCCCGATCCAGTGCTAGGACAAGGATGATCGTC

> comp75688_1900

CCTAGGAACCTCCTCGCTGCACGCAGCTGCTTCCCCCTCACTCCCCAGCCGATTTCTCTGCGCGCCCAGCCCAGCCACTCGCCATGGCTCCTCCCTGCGCGCGTCGAAGTTCCTCTGCTCGTCCCATCTCCCCAAAGCTTCTTCCTCCCTGCTCGGCGCTTCCTATTCCCAGCGCTCGACCGCCCCAGCTCGCGCCCAATCCTCCTGCTTGGATTTCGTCCAGATCGAGCTGCTCCCATGGCGATTTCCCCTCGGATTCCTCCCTGCTCGTGCCCCATCGCCATGAACGTGCGCGAGTTTCCACTGGTGGTCGTCGCGCTGGACACCTCCCCTCCGGTCTCCTCTCTCAAGCGCTGCTTCTTCTTCCTGTTGCCGCGCGCCCAGCCGCCTTTCCTTTTCCTCTCGACGCCCGGCCGAGCTCCTCCCTCGCCCATGGCCGCGGTTCTCCAAGTTCCCCTGCGCGCGCGGTCCTCTGTCCCTGCTCGCCCTCTGCGCGTGCTTCTCCCTGCTCCTATGCGCGCGCGGTTCTCTGCTCCCCCGCGCGCTCGTTCTGTTGCTTTTTCCAGCACGCCGCGCGCAGCTCCCTCTAGCTCTGACCATGCCGCGTCGCGTTCCCGTTCCCAGCCATGGTGGTCGATCCCTGCTGCCCAGCTCCTTCGGCACCCAGCCAGGCCGTCCTGGATATTGCGCGCTCCTCCCGGTTGCCATCGCTCGCCGTCGCCTCCTCGTGCTGGCCGACTAGTCTGTCGTGGTCGCACGCAGTTCCCTGCTCGGCCAGGATGGTCGCGTCGTCGATCCCGCCCGGACCTGCAGCCCCAACACACTGTTTCTCTGCTCGGTGCCAAGTATTCTACTACGTCACACCGTGTCCACAGTAAGCCGTGAAGACGCTACTATGCTCGCTGCTCGCCACCTGCCAACATAATGCAAGAACCGGCTATCGATCTTGTCTCTACCATCGCCTTCGCTTTACATCTTTGCTCCCGTTGTCGCGGAGACCCTGCCAGCCCTGCACGTCGTCCACGCTTTGTTGTGTTCGCGTGGAGGGGAATTCTGGGAAATTGGGTGAAGAAACCCGCATGGCGTTTACCGGGTGCTCGACAGATAGCTCATATCGGAGATCGTCGTCGTTCCGCGTGTCAGCAAGAAATTTCAAAAATCGGGTGAAGACGAAGCTAGCAGCGTGGTGTTCACCAAGTGCGCGACAAAAGCTCGGACATCCTGCGCGACTCAAGTTCGATTCGTCCGAATTATTCAATCGAAGAAAAGATCTTCAACAATTGATTACCGAAGAAAAGAAGGCCCCTAGATGACGTCAACCGGAACCCGTAGTCGTGATTGCGACTATCGCGTCGAATTAGTCAGGGTAAGTTGATTCATATTCCCGTTAACTGGGTCTATAAATAGAGTGAATAGAATTATTGGTCTCAATAAATGTTATTAATTTAGCACGTGTATATTGTGATGATCGATTGAGATAAATCGGTAAATCAAGTAATTAAGATACATAGTTTAAAGAGTTAATAAAAAGCTAGGAATAAAAATAGATTTTCTAGTGTAATATGTCAATTTGGTTAATGGATGTAGTCGATGCCCTGCTGTGATATCCATGGTGCTTAACGTTTGGTTTTTAAGTGTTTCATCCTTTTTAGTGTAGATTATCTATATTATACTCGTATACTCGTGCTCATACATGTGCATCATGCATCTCATGAGGTACGATAAATAATCACGTGATGCGGAAGAAGAGCCAAGTCGACCCCAAGCGCGGGCAAAT

>a5_12 [Full length sequence from B73+B]

GTCTTCTTCCTCGCGGTGTCCAGCCTCAACTCAGATTCCCGGCGTCCTCTCCATGGCTCCCTACGCCCTCGTCTTCCCCCTGGTGCCGCGCGGGCGCGCCCCTGGAACCTCCTCGCTGCACGCAGCTGCTTCCCCCTCACTCCCCAGCCGATTTCTCTGCGCGCCCAGCCCAGCCACTCGCCATGGCTCCTCCCTGCGCGCGTCGAAGTTCCTCTGCTCGTCCCATCTCCCCAAAGCTTCTTCCTCCCTGCTCGGCGCTTCCTATTCCCAGCGCTCGACCGCCCCAGCTCGCGCCCAATCCTCCTGCTTGGATTTCGTCCAGATCGAGCTGCTCCCATGGCGATTTCCCCTCGGATTCCTCCCTGCTCGTGCCCCATCGCCATGAACGTGCGCGAGTTTCCACTGGTGGTCGTCGCGCTGGACACCTCCCCTCCGGTCTCCTCTCTCAAGCGCTGCTTCTTCTTCCTGTTGCCGCGCGCCCAGCCGCCTTTCCTTTTCCTCTCGACGCCCGGCCGAGCTCCTCCCTCGCCCATGGCCGCGGTTCTCCAAGTTCCCCTGCGCGCGCGGTCCTCTGTCCCTGCTCGCCCTCTGCGCGTGCTTCTCCCTGCTCCTATGCGCGCGCGGTTCTCTGCTCCCCCGCGCGCTCGTTCTGTTGCTTTTTCCAGCACGCCGCGCGCAGCTCCCTCTAGCTCTGACCATGCCGCGTCGCGTTCCCGTTCCCAGCCATGGTGGTCGATCCCTGCTGCCCAGCTCCTTCGGCACCCAGCCAGGCCGTCCTGGATATTGCGCGCTCCTCCCGGTTGCCATCGCTCGCCGTCGCCTCCTCGTGCTGGCCGACTAGTCTGTCGTGGTCGCACGCAGTTCCCTGCTCGGCCAGGATGGTCGCGTCGTCGATCCCGCCCGGACCTGCAGCCCCAACACACTGTTTCTCTGCTCGGTGCCAAGTATTCTACTACGTCACACCGTGTCCACAGTAAGCCGTGAAGACGCTACTATGCTCGCTGCTCGCCACCTGCCAACATAATGCAAGAACCGGCTATCGATCTTGTCTCTACCATCGCCTTCGCTTTACATCTTTGCTCCCGTTGTCGCGGAGACCCTGCCAGCCCTGCACGTCGTCCACGCTTTGTTGTGTTCGCGTGGAGGGGAATTCTGGGAAATTGGGTGAAGAAACCCGCATGGCGTTTACCGGGTGCTCGACAGATAGCTCATATCGGAGATCGTCGTCGTTCCGCGTGTCAGCAAGAAATTTCAAAAATCGGGTGAAGACGAAGCTAGCAGCGTGGTGTTCACCAAGTGCGCGACAAAAGCTCGGACATCCTGCGCGACTCAAGTTCGATTCGTCCGAATTATTCAATCGAAGAAAAGATCTTCAACAATTGATTACCGAAGAAAAGAAGGCCCCTAGATGACGTCAACCGGAACCCGTAGTCGTGATTGCGACTATCGCGTCGAATTAGTCAGGGTAAGTTGATTCATATTCCCGTTAACTGGGTCTATAAATAGAGTGAATAGAATTATTGGTCTCAATAAATGTTATTAATTTAGCACGTGTATATTGTGATGATCGATTGAGATAAATCGGTAAATCAAGTAATTAAGATACATAGTTTAAAGAGTTAATAAAAAGCTAGGAATAAAAATAGATTTTCTAGTGTAATATGTCAATTTGGTTAATGGATGTAGTCGATGCCCTGCTGTGATATCCATGGTGCTTAACGTTTGGTTTTTAAGTGTTTCATCCTTTTTAGTGTAGATTATCTATATTATACTCGTATACTCGTGCTCATACATGTGCATCATGCATCTCATGAGGTACGATAAATAATCACGTGATGCGGAAGAAGAGCCAAGTCGACCCCAAGCGCGGGCAAATCCGCAGGATAATGCTGATGGACGAACCTGCCCGATCCAGTGCTAGGACAAGGATGATCGTCAAGTGGTCATCTAACAACACTAACCTAGTGTTACTCAGGCAAGCCCCGGTGCATTTGCCACCTCCTTGTGTTTTTAAAATCTTTTTTCACTTGCTTGATAAATTAGGTGATAGGAGTTGATTGCTAAAACAATTCCTGCATTACCTTCCTTGATCACCCGTTTTACAAAAGATTTTTGATGCTTAGCCTTGCTCTAGAAAAACAAAAGGTTTTGTTTTTACAAAAGATGTTGTGGCAAAAGTGGGTGGGATGTTTTCGAAAATAAAACTTGATGGTGGATCCATCATGGCCGTGATGAGTTCAACATCGGAAAAGATGTACCTCTGCCAGGTATCAAGTTTTTGGGTTGAAATGATTAAGCTGAGACCGGGCGGGTGACTTGCACGAGAAAGGAGTCTCGATGTAGTGTCTCCGTCTGAGTCGATTAAGGACCGTGTCGATGTAGGCTTGATGACCGAGGACCCTTTAACTGGTCACATGCCTCGTCATGGGTAAGCCTTGCCTCTGGCAGACTAAGGCCAGAATAAGATAACACGAAATGGGCGTGGAGCGGTGGCGAGAGTAGCGTGTACCCTCCATAGCAAGAGGCTGGACGGTGGTGTATTTGTGCTCTCGGTTTGCGTGAACCTGATCTGGTCTTAAGAACCCCGGTGGCGGGTTGACATATGCAAGGGTTATGTGCTACAAATGTCGTGTGATTGGGGATCCTCAGCTGAGTATAATCGATTCGGATCGCCGTACCTTCGCGGTTATGAAGACTTGGTCACTGACCTATACATAAAACTCCAGTAAAGATGAAGGGAATGATAAGAAATTGGCTAGTGCAGGTCAAGTGCTTGAACTAGGGTAGAAAGAACTCTAGTTGCAGGTAATTTTACTTAACTTGACAAATAAAACTAGATTTTTAAGGATCCACTTTAGTAAGCATTTCTGCAAAACAGAGTCTTTGATTATTGAGAAGCCTTACCTTGACTCCCTTAACCAGCATACCCTTGAGAGTCTTTTCTTTAGTCGGGTAAGACTTGCTGAGTAATTCCATACTCAGGGTTTTATTCCTTGTTGTTTTTCAGGTTCTAACTTTGTGCTGTTGTTGATGGTGTTAAGTGCCGGTGGGCTCGGCCTTCTTATATAAGTATACCCCGTCTTTATCTTCTTATTGAGGATGGTCACTTGAGCTAGCATATATTTCAAAACTATAAATAATTTATAGCAGTTCAAAGTTTCTTTCTTTGAATCACTGTAGTAATCACTCCGATCATGTACGAAGTAAAATTTTTGTAACTTGTAAAATTTGGTAATAAGATTTCCGCTGGAA

>a5_13 [Full length from B73+B]

GTCTTCTTCCTCGCGGTGTCCAGCCTCAACTCAGATTCCCGGCGTCCTCTCCATGGCTCCCTACGCCCTCGTCTTCCCCCTGGTGCCGCGCGGGCGCGCCCCTGGAACCTCCTCGCTGCACGCAGCTGCTTCCCCCTCACTCCCCAGCCGATTTCTCTGCGCGCCCAGCCCAGCCACTCGCCATGGCTCCTCCCTGCGCGCGTCGAAGTTCCTCTGCTCGTCCCATCTCCCCAAAGCTTCTTCCTCCCTGCTCGGCGCTTCCTATTCCCAGCGCTCGACCGCCCCAGCTCGCGCCCAATCCTCCTGCTTGGATTTCGTCCAGATCGAGCTGCTCCCATGGCGATTTCCCCTCGGATTCCTCCCTGCTCGTGCCCCATCGCCATGAACGTGCGCGAGTTTCCACTGGTGGTCGTCGCGCTGGACACCTCCCCTCCGGTCTCCTCTCTCAAGCGCTGCTTCTTCTTCCTGTTGCCGCGCGCCCAGCCGCCTTTCCTTTTCCTCTCGACGCCCGGCCGAGCTCCTCCCTCGCCCATGGCCGCGGTTCTCCAAGTTCCCCTGCGCGCGCGGTCCTCTGTCCCTGCTCGCCCTCTGCGCGTGCTTCTCCCTGCTCCTATGCGCGCGCGGTTCTCTGCTCCCCCGCGCGCTCGTTCTGTTGCTTTTTCCAGCACGCCGCGCGCAGCTCCCTCTAGCTCTGACCATGCCGCGTCGCGTTCCCGTTCCCAGCCATGGTGGTCGATCCCTGCTGCCCAGCTCCTTCGGCACCCAGCCAGGCCGTCCTGGATATTGCGCGCTCCTCCCGGTTGCCATCGCTCGCCGTCGCCTCCTCGTGCTGGCCGACTAGTCTGTCGTGGTCGCACGCAGTTCCCTGCTCGGCCAGGATGGTCGCGTCGTCGATCCCGCCCGGACCTGCAGCCCCAACACACTGTTTCTCTGCTCGGTGCCAAGTATTCTACTACGTCACACCGTGTCCACAGTAAGCCGTGAAGACGCTACTATGCTCGCTGCTCGCCACCTGCCAACATAATGCAAGAACCGGCTATCGATCTTGTCTCTACCATCGCCTTCGCTTTACATCTTTGCTCCCGTTGTCGCGGAGACCCTGCCAGCCCTGCACGTCGTCCACGCTTTGTTGTGTTCGCGTGGAGGGGAATTCTGGGAAATTGGGTGAAGAAACCCGCATGGCGTTTACCGGGTGCTCGACAGATAGCTCATATCGGAGATCGTCGTCGTTCCGCGTGTCAGCAAGAAATTTCAAAAATCGGGTGAAGACGAAGCTAGCAGCGTGGTGTTCACCAAGTGCGCGACAAAAGCTCGGACATCCTGCGCGACTCAAGTTCGATTCGTCCGAATTATTCAATCGAAGAAAAGATCTTCAACAATTGATTACCGAAGAAAAGAAGGCCCCTAGATGACGTCAACCGGAACCCGTAGTCGTGATTGCGACTATCGCGTCGAATTAGTCAGGGTAAGTTGATTCATATTCGCGTTAACTGGGTCTATAAATAGAGTGAATAGAATTATTGGTCTCAATAAATGTTATTAATTTAGCACGTGTATATTGTGATGATCGATTGAGATAAATCGGTAAATCAAGTAATTAAGATACATAGTTTAAAGAGTTAATAAAAAGCTAGGAATAAAAATAGATTTTCTAGTGTAATATGTCAATTTGGTTAATGGATGTAGTCGATGCCCTGCTGTGATATCCATGGTGCTTAACGTTTGGTTTTTAAGTGTTTCATCCTTTTTAGTGTAGATTATCTATATTATACTCGTATACTCGTGCTCATACATGTGCATCATGCATCTCATGAGGTACGATAAATAATCACGTGATGCGGAAGAAGAGCCAAGTCGACCCCAAGCGCGGGCAAATCCGCAGGATAATGCTGATGGACGAACCTGCCCGATCCAGTGCTAGGACAAGGATGATCGTCAAGTGGTCATCTAACAACACTAACCTAGTGTTACTCAGGCAAGCCCCGGTGCATTTGCCACCTCCTTGTGTTTTTAAAATCTTTTTTCACTTGCTTGATAAATTAGGTGATAGGAGTTGATTGCTAAAACAATTCCTGCATTACCTTCCTTGATCACCCGTTTTACAAAAGATTTTTGATGCTTAGCCTTGCTCTAGAAAAACAAAAGGTTTTGTTTTTACAAAAGATGTTGTGGCAAAAGTGGGTGGGATGTTTTCGAAAATAAAACTTGATGGTGGATCCATCATGGCCGTGATGAGTTCAACATCGGAAAAGATGTACCTCTGCCAGGTATCAAGTTTTTGGGTTGAAATGATTAAGCTGAGACCGGGCGGGTGACTTGCACGAGAAAGGAGTCTCGATGTAGTGTCTCCGTCTGAGTCGATTAAGGACCGTGTCGATGTAGGCTTGATGACCGAGGACCCTTTAACTGGTCACATGCCTCGTCATGGGTAAGCCTTGCCTCTGGCAGACTAAGGCCAGAATAAGATAACACGAAATGGGCGTGGAGCGGTGGCGAGAGTAGCGTGTACCCTCCATAGCAAGAGGCTGGACGGTGGTGTATTTGTGCTCTCGGTTTGCGTGAACCTGATCTGGTCTTAAGAACCCCGGTGGCGGGTTGACATATGCAAGGGTTATGTGCTACAAATGTCGTGTGATTGGGGATCCTCAGCTGAGTATAATCGATTCGGATCGCCGTACCTTCGCGGTTATGAAGACTTGGTCACTGACCTATACATAAAACTCCAGTAAAGATGAAGGGAATGATAAGAAATTGGCTAGTGCAGGTCAAGTGCTTGAACTAGGGTAGAAAGAACTCTAGTTGCAGGTAATTTTACTTAACTTGACAAATAAAACTAGATTTTTAAGGATCCACTTTAGTAAGCATTTCTGCAAAACAGAGTCTTTGATTATTGAGAAGCCTTACCTTGACTCCCTTAACCAGCATACCCTTGAGAGTCTTTTCTTTAGTCGGGTAAGACTTGCTGAGTAATTCCATACTCAGGGTTTTATTCCTTGTTGTTTTTCAGGTTCTAACTTTGTGCTGTTGTTGATGGTGTTAAGTGCCGGTGGGCTCGGCCTTCTTATATAAGTATACCCCGTCTTTATCTTCTTATTGAGGATGGTCACTTGAGCTAGCATATATTTCAAAACTATAAATAATTTATAGCAGTTCAAAGTTTCTTTCTTTGAATCACTGTAGTAATCACTCCGATCATGTACGAAGTAAAATTTTTGTAACTTGTAAAATTTGGTAATAAGATTTCCGCTGGAA

>a4_1 [Full length sequence from Starter+B]

GGAAGCCAAGCGCCCACGTCTTCTTCCTCGCGGTGTCCAGCCTCAACTCAGATTCCCGGCGTCCTCTCCATGGCTCCCTACGCCCTCGTCTTCCCCCTGGTGCCGCGCGGGCGCGCCCCTGGAACCTCCTCGCTGCACGCAGCTGCTTCCCCCTCACTCCCCAGCCGATTTCTCTGCGCGCCCAGCCCAGCCACTCGCCATGGCTCCTCCCTGCGCGCGTCGAAGTTCCTCTGCTCGTCCCATCTCCCCAAAGCTTCTTCCTCCCTGCTCGGCGCTTCCTATTCCCAGCGCTCGACCGCCCCAGCTCGCGCCCAATCCTCCTGCTTGGATTTCGTCCAGATCGAGCTGCTCCCATGGCGATTTCCCCTCGGATTCCTCCCTGCTCGTGCCCCATCGCCATGAACGTGCGCGAGTTTCCACTGGTGGTCGTCGCGCTGGACACCTCCCCTCCGGTCTCCTCTCTCAAGCGCTGCTTCTTCTTCCTGTTGCCGCGCGCCCAGCCGCCTTTCCTTTTCCTCTCGACGCCCGGCCGAGCTCCTCCCTCGCCCATGGCCGCGGTTCTCCAAGTTCCCCTGCGCGCGCGGTCCTCTGTCCCTGCTCGCCCTCTGCGCGTGCTTCTCCCTGCTCCTATGCGCGCGCGGTTCTCTGCTCCCCCGCGCGCTCGTTCTGTTGCTTTTTCCAGCACGCCGCGCGCAGCTCCCTCTAGCTCTGACCATGCCGCGTCGCGTTCCCGTTCCCAGCCATGGTGGTCGATCCCTGCTGCCCAGCTCCTTCGGCACCCAGCCAGGCCGTCCTGGATATTGCGCGCTCCTCCCGGTTGCCATCGCTCGCCGTCGCCTCCTCGTGCTGGCCGACTAGTCTGTCGTGGTCGCACGCAGTTCCCTGCTCGGCCAGGATGGTCGCGTCGTCGATCCCGCCCGGACCTGCAGCCCCAACACACTGTTTCTCTGCTCGGTGCCAAGTATTCTACTACGTCACACCGTGTCCACAGTAAGCCGTGAAGACGCTACTATGCTCGCTGCTCGCCACCTGCCAACATAATGCAAGAACCGGCTATCGATCTTGTCTCTACCATCGCCTTCGCTTTACATCTTTGCTCCCGTTGTCGCGGAGACCCTGCCAGCCCTGCACGTCGTCCACGCTTTGTTGTGTTCGCGTGGAGGGGAATTCTGGGAAATTGGGTGAAGAAACCCGCATGGCGTTTACCGGGTGCTCGACAGATAGCTCATATCGGAGATCGTCGTCGTTCCGCGTGTCAGCAAGAAATTTCAAAAATCGGGTGAAGACGAAGCTAGCAGCGTGGTGTTCACCAAGTGCGCGACAAAAGCTCGGACATCCTGCGCGACTCAAGTTCGATTCGTCCGAATTATTCAATCGAAGAAAAGATCTTCAACAATTGATTACCGAAGAAAAGAAGGCCCCTAGATGACGTCAACCGGAACCCGTAGTCGTGATTGCGACTATCGCGTCGAATTAGTCAGGGTAAGTTGATTCATATTCCCGTTAACTGGGTCTATAAATAGAGTGAATAGAATTATTGGTCTCAATAAATGTTATTAATTTAGCACGTGTATATTGTGATGATCGATTGAGATAAATCGGTAAATCAAGTAATTAAGATACATAGTTTAAAGAGTTAATAAAAAGCTAGGAATAAAAATAGATTTTCTAGTGTAATATGTCAATTTGGTTAATGGATGTAGTCGATGCCCTGCTGTGATATCCATGGTGCTTAACGTTTGGTTTTTAAGTGTTTCATCCTTTTTAGTGTAGATTATCTATATTATACTCGTATACTCGTGCTCATACATGTGCATCATGCATCTCATGAGGTACGATAAATAATCACGTGATGCGGAAGAAGAGCCAAGTCGACCCCAAGCGCGGGCAAATCCGCAGGATAATGCTGATGGACGAACCTGCCCGATCCAGTGCTAGGACAAGGATGATCGTCAAGTGGTCATCTAACAACACTAACCTAGTGTTACTCAGGCAAGCCCCGGTGCATTTGCCACCTCCTTGTGTTTTTAAAATCTTTTTTCACTTGCTTGATAAATTAGGTGATAGGAGTTGATTGCTAAAACAATTCCTGCATTACCTTCCTTGATCACCCGTTTTACAAAAGATTTTTGATGCTTAGCCTTGCTCTAGAAAAACAAAAGGTTTTGTTTTTACAAAAGATGTTGTGGCAAAAGTGGGTGGGATGTTTTCGAAAATAAAACTTGATGGTGGATCCATCATGGCCGTGATGAGTTCAACATCGGAAAAGATGTACCTCTGCCAGGTACCAAGTTTTTGGGTTGAAATGATTAAGCTGAGACCGGGCGGGTGACTTGCACGAGAAAGGAGTCTCGATGTAGTGTCTCCGTCTGAGTCGATTAAGGACCGTGTCGATGTAGGCTTGATGACCGAGGACCCTTTAACTGGTCACATGCCTCGTCATGGGTAAGCCTTGCCTCTGGCAGACTAAGGCCAGAATAAGATAACACGAAATGGGCGTGGAGCGGTGGCGAGAGTAGCGTGTACCCTCCATAGCAAGAGGCTGGACGGTGGTGTATTTGTGCTCTCGGTTTGCGTGAACCTGATCTGGTCTTAAGAACCCCGGTGGCGGGTTGACATATGCAAGGGTTATGTGCTACAAATGTCGTGTGATTGGGGATCCTCAGCTGAGTATAATCGATTCGGATCGCCGTACCTTCGCGGTTATGAAGACTTGGTCACTGACCTATACATAAAACTCCAGTAAAGATGAAGGGAATGATAAGAAATTGGCTAGTGCAGGTCAAGTGCTTGAACTAGGGTAGAAAGAACTCTAGTTGCAGGTAATTTTACTTAACTTGACAAATAAAACTGGATTTTTAAGGATCCACTTTAGTAAGCATTTCTGCAAAACAGAGTCTTTGATTATTGAGAAGCCTTACCTTGACTCCCTTAACCAGCATACCCTTGAGAGTCTTTTCTTTAGTCGGGTAAGACTTGCTGAGTAATTCCATACTCAGGGTTTTATTCCTTGTTGTTTTTCAGGTTCTAACTTTGTGCTGTTGTTGATGGTGTTAAGTGCCGGTGGGCTCGGCCTTCTTATATAAGTATACCCCGTCTTTATCTTCTTATTGAGGATGGTCACTTGAGCTAGCATATATTTCAAAACTATAAATAATTTATAGCAGTTCAAAGTTTCTTTCTTTGAATCACTGTAGTAATCACTCCGATCATGTACGAAGTAAAATTTTTGTAACTTGTAAAATTTGGTAATAAGATTTCCGCTGGAACGGGCAATTCTAAGCACACT

>a4_2 [Full length sequence from Starter+B]

GTCTTCTTCCTCGCGGTGTCCAGCCTCAACTCAGATTCCCGGCGTCCTCTCCATGGCTCCCTACGCCCTCGTCTTCCCCCTGGTGCCGCGCGGGCGCGCCCCTGGAACCTCCTCGCTGCACGCAGCTGCTTCCCCCTCACTCCCCAGCCGATTTCTCTGCGCGCCCAGCCCAGCCACTCGCCATGGCTCCTCCCTGCGCGCGTCGAAGTTCCTCTGCTCGTCCCATCTCCCCAAAGCTTCTTCCTCCCTGCTCGGCGCTTCCTATTCCCAGCGCTCGACCGCCCCAGCTCGCGCCCAATCCTCCTGCTTGGATTTCGTCCAGATCGAGCTGCTCCCATGGCGATTTCCCCTCGGATTCCTCCCTGCTCGTGCCCCATCGCCATGAACGTGCGCGAGTTTCCACTGGTGGTCGTCGCGCTGGACACCTCCCCTCCGGTCTCCTCTCTCAAGCGCTGCTTCTTCTTCCTGTTGCCGCGCGCCCAGCCGCCTTTCCTTATCCTCTCGACGCCCGGCCGAGCTCCTCCCTCGCCCATGGCCGCGGTTCTCCAAGTTCCCCTGCGCGCGCGGTCCTCTGTCCCTGCTCGCCCTCTGCGCGTGCTTCTCCCTGCTCCTATGCGCGCGCGGTTCTCTGCTCCCCCGCGCGCTCGTTCTGTTGCTTTTTCCAGCACGCCGCGCGCAGCTCCCTCTAGCTCTGACCATGCCGCGTCGCGTTCCCGTTCCCAGCCATGGTGGTCGATCCCTGCTGCCCAGCTCCTTCGGCACCCAGCCAGGCCGTCCTGGATATTGCGCGCTCCTCCCGGTTGCCATCGCTCGCCGTCGCCTCCTCGTGCTGGCCGACTAGTCTGTCGTGGTCGCACGCAGTTCCCTGCTCGGCCAGGATGGTCGCGTCGTCGATCCCGCCCGGACCTGCAGCCCCAACACACTGTTTCTCTGCTCGGTGCCAAGTATTCTACTACGTCACACCGTGTCCACAGTAAGCCGTGAAGACGCTACTATGCTCGCTGCTCGCCACCTGCCAACATAATGCAAGAACCGGCTATCGATCTTGTCTCTACCATCGCCTTCGCTTTACATCTTTGCTCCCGTTGTCGCGGAGACCCTGCCAGCCCTGCACGTCGTCCACGCTTTGTTGTGTTCGCGTGGAGGGGAATTCTGGGAAATTGGGTGAAGAAACCCGCATGGCGTTTACCGGGTGCTCGACAGATAGCTCATATCGGAGATCGTCGTCGTTCCGCGTGTCAGCAAGAAATTTCAAAAATCGGGTGAAGACGAAGCTAGCAGCGTGGTGTTCACCAAGTGCGCGACAAAAGCTCGGACATCCTGCGCGACTCAAGTTCGATTCGTCCGAATTATTCAATCGAAGAAAAGATCTTCAACAATTGATTACCGAAGAAAAGAAGGCCCCTAGATGACGTCAACCGGAACCCGTAGTCGTGATTGCGACTATCGCGTCGAATTAGTCAGGGTAAGTTGATTCATATTCCCGTTAACTGGGTCTATAAATAGAGTGAATAGAATTATTGGTCTCAATAAATGTTATTAATTTAGCACGTGTATATTGTGATGATCGATTGAGATAAATCGGTAAATCAAGTAATTAAGATACATAGTTTAAAGAGTTAATAAAAAGCTAGGAATAAAAATAGATTTTCTAGTGTAATATGTCAATTTGGTTAATGGATGTAGTCGATGCCCTGCTGTGATATCCATGGTGCTTAACGTTTGGTTTTTAAGTGTTTCATCCTTTTTAGTGTAGATTATCTATATTATACTCGTATACTCGTGCTCATACATGTGCATCATGCATCTCATGAGGTACGATAAATAATCACGTGATGCGGAAGAAGAGCCAAGTCGACCCCAAGCGCGGGCAAATCCGCAGGATAATGCTGATGGACGAACCTGCCCGATCCAGTGCTAGGACAAGGATGATCGTCAAGTGGTCATCTAACAACACTAACCTAGTGTTACTCAGGCAAGCCCCGGTGCATTTGCCACCTCCTTGTGTTTTTAAAATCTTTTTTCACTTGCTTGATAAATTAGGTGATAGGAGTTGATTGCTAAAACAATTCCTGCATTACCTTCCTTGATCACCCGTTTTACAAAAGATTTTTGATGCTTAGCCTTGCTCTAGAAAAACAAAAGGTTTTGTTTTTACAAAAGATGTTGTGGCAAAAGTGGGTGGGATGTTTTCGAAAATAAAACTTGATGGTGGATCCATCATGGCCGTGATGAGTTCAACATCGGAAAAGATGTACCTCTGCCAGGTACCAAGTTTTTGGGTTGAAATGATTAAGCTGAGACCGGGCGGGTGACTTGCACGAGAAAGGAGTCTCGATGTAGTGTCTCCGTCTGAGTCGATTAAGGACCGTGTCGATGTAGGCTTGATGACCGAGGACCCTTTAACTGGTCACATGCCTCGTCATGGGTAAGCCTTGCCTCTGGCAGACTAAGGCCAGAATAAGATAACACGAAATGGGCGTGGAGCGGTGGCGAGAGTAGCGTGTACCCTCCATAGCAAGAGGCTGGACGGTGGTGTATTTGTGCTCTCGGTTTGCGTGAACCTGATCTGGTCTTAAGAACCCCGGTGGCGGGTTGACATATGCAAGGGTTATGTGCTACAAATGTCGTGTGATTGGGGATCCTCAGCTGAGTATAATCGATTCGGATCGCCGTACCTTCGCGGTTATGAAGACTTGGTCACTGACCTATACATAAAACTCCAGTAAAGATGAAGGGAATGATAAGAAATTGGCTAGTGCAGGTCAAGTGCTTGAACTAGGGTAGAAAGAACTCTAGTTGCAGGTAATTTTACTTAACTTGACAAATAAAACTGGATTTTTAAGGATCCACTTTAGTAAGCATTTCTGCAAAACAGAGTCTTTGATTATTGAGAAGCCTTACCTTGACTCCCTTAACCAGCATACCCTTGAGAGTCTTTTCTTTAGTCGGGTAAGACTTGCTGAGTAATTCCATACTCAGGGTTTTATTCCTTGTTGTTTTTCAGGTTCTAACTTTGTGCTGTTGTTGATGGTGTTAAGTGCCGGTGGGCTCGGCCTTCTTATATAAGTATACCCCGTCTTTATCTTCTTATTGAGGATGGTCACTTGAGCTAGCATATATTTCAAAACTATAAATAATTTATAGCAGTTCAAAGTTTCTTTCTTTGAATCACTGTAGTAATCACTCCGATCATGTACGAAGTAAAATTTTTGTAACTTGTAAAATTTGGTAATAAGATTTCCGCTGGAA

>a4_4 [Full length sequence from Starter+B]

GTCTTCTTCCTCGCGGTGTCCAGCCTCAACTCAGATTCCCGGCGTCCTCTCCATGGCTCCCTACGCCCTCGTCTTCCCCCTGGTGCCGCGCGGGCGCGCCCCTGGAACCTCCTCGCTGCACGCAGCTGCTTCCCCCTCACTCCCCAGCCGATTTCTCTGCGCGCCCAGCCCAGCCACTCGCCATGGCTCCTCCCTGCGCGCGTCGAAGTTCCTCTGCTCGTCCCATCTCCCCAAAGCTTCTTCCTCCCTGCTCGGCGCTTCCTATTCCCAGCGCTCGACCGCCCCAGCTCGCGCCCAATCCTCCTGCTTGGATTTCGTCCAGATCGAGCTGCTCCCATGGCGATTTCCCCTCGGATTCCTCCCTGCTCGTGCCCCATCGCCATGAACGTGCGCGAGTTTCCACTGGTGGTCGTCGCGCTGGACACCTCCCCTCCGGTCTCCTCTCTCAAGCGCTGCTTCTTCTTCCTGTTGCCGCGCGCCCAGCCGCCTTTCCTTTTCCTCTCGACGCCCGGCCGAGCTCCTCCCTCGCCCATGGCCGCGGTTCTCCAAGTTCCCCTGCGCGCGCGGTCCTCTGTCCCTGCTCGCCCTCTGCGCGTGCTTCTCCCTGCTCCTATGCGCGCGCGGTTCTCTGCTCCCCCGCGCGCTCGTTCTGTTGCTTTTTCCAGCACGCCGCGCGCAGCTCCCTCTAGCTCTGACCATGCCGCGTCGCGTTCCCGTTCCCAGCCATGGTGGTCGATCCCTGCTGCCCAGCTCCTTCGGCACCCAGCCAGGCCGTCCTGGATATTGCGCGCTCCTCCCGGTTGCCATCGCTCGCCGTCGCCTCCTCGTGCTGGCCGACTAGTCTGTCGTGGTCGCACGCAGTTCCCTGCTCGGCCAGGATGGTCGCGTCGTCGATCCCGCCCGGACCTGCAGCCCCAACACACTGTTTCTCTGCTCGGTGCCAAGTATTCTACTACGTCACACCGTGTCCACAGTAAGCCGTGAAGACGCTACTATGCTCGCTGCTCGCCACCTGCCAACATAATGCAAGAACCGGCTATCGATCTTGTCTCTACCATCGCCTTCGCTTTACATCTTTGCTCCCGTTGTCGCGGAGACCCTGCCAGCCCTGCACGTCGTCCACGCTTTGTTGTGTTCGCGTGGAGGGGAATTCTGGGAAATTGGGTGAAGAAACCCGCATGGCGTTTACCGGGTGCTCGACAGATAGCTCATATCGGAGATCGTCGTCGTTCCGCGTGTCAGCAAGAAATTTCAAAAATCGGGTGAAGACGAAGCTAGCAGCGTGGTGTTCACCAAGTGCGCGACAAAAGCTTGGACATCCTGCGCGACTCAAGTTCGATTCGTCCGAATTATTCAATCGAAGAAAAGATCTTCAACAATTGATTACCGAAGAAAAGAAGGCCCCTAGATGACGTCAACCGGAACCCGTAGTCGTGATTGCGACTATCGCGTCGAATTAGTCAGGGTAAGTTGATTCATATTCCCGTTAACTGGGTCTATAAATAGAGTGAATAGAATTATTGGTCTCAATAAATGTTATTAATTTAGCACGTGTATATTGTGATGATCGATTGAGATAAATCGGTAAATCAAGTAATTAAGATACATAGTTTAAAGAGTTAATAAAAAGCTAGGAATAAAAATAGATTTTCTAGTGTAATATGTCAATTTGGTTAATGGATGTAGTCGATGCCCTGCTGTGATATCCATGGTGCTTAACGTTTGGTTTTTAAGTGTTTCATCCTTTTTAGTGTAGATTATCTATATTATACTCGTATACTCGTGCTCATACATGTGCATCATGCATCTCATGAGGTACGATAAATAATCACGTGATGCGGAAGAAGAGCCAAGTCGACCCCAAGCGCGGGCAAATCCGCAGGATAATGCTGATGGACGAACCTGCCCGATCCAGTGCTAGGACAAGGATGATCGTCAAGTGGTCATCTAACAACACTAACCTAGTGTTACTCAGGCAAGCCCCGGTGCATTTGCCACCTCCTTGTGTTTTTAAAATCTTTTTTCACTTGCTTGATAAATTAGGTGATAGGAGTTGATTGCTAAAACAATTCCTGCATTACCTTCCTTGATCACCCGTTTTACAAAAGATTTTTGATGCTTAGCCTTGCTCTAGAAAAACAAAAGGTTTTGTTTTTACAAAAGATGTTGTGGCAAAAGTGGGTGGGATGTTTTCGAAAATAAAACTTGATGGTGGATCCATCATGGCCGTGATGAGTTCAACATCGGAAAAGATGTACCTCTGCCAGGTACCAAGTTTTTGGGTTGAAATGATTAAGCTGAGACCGGGCGGGTGACTTGCACGAGAAAGGAGTCTCGATGTAGTGTCTCCGTCTGAGTCGATTAAGGACCGTGTCGATGTAGGCTTGATGACCGAGGACCCTTTAACTGGTCACATGCCTCGTCATGGGTAAGCCTTGCCTCTGGCAGACTAAGGCCAGAATAAGATAACACGAAATGGGCGTGGAGCGGTGGCGAGAGTAGCGTGTACCCTCCATAGCAAGAGGCTGGACGGTGGTGTATTTGTGCTCTCGGTTTGCGTGAACCTGATCTGGTCTTAAGAACCCCGGTGGCGGGTTGACATATGCAAGGGTTATGTGCTACAAATGTCGTGTGATTGGGGATCCTCAGCTGAGTATAATCGATTCGGATCGCCGTACCTTCGCGGTTATGAAGACTTGGTCACTGACCTATACATAAAACTCCAGTAAAGATGAAGGGAATGATAAGAAATTGGCTAGTGCAGGTCAAGTGCTTGAACTAGGGTAGAAAGAACTCTAGTTGCAGGTAATTTTACTTAACTTGACAAATAAAACTGGATTTTTAAGGATCCACTTTAGTAAGCATTTCTGCAAAACAGAGTCTTTGATTATTGAGAAGCCTTACCTTGACTCCCTTAACCAGCATACCCTTGAGAGTCTTTTCTTTAGTCGGGTAAGACTTGCTGAGTAATTCCATACTCAGGGTTTTATTCCTTGTTGTTTTTCAGGTTCTAACTTTGTGCTGTTGTTGATGGTGTTAAGTGCCGGTGGGCTCGGCCTTCTTATATAAGTATACCCCGTCTTTATCTTCTTATTGAGGATGGTCACTTGAGCTAGCATATATTTCAAAACTATAAATAATTTATAGCAGTTCAAAGTTTCTTTCTTTGAATCACTGTAGTAATCACTCCGATCATGTACGAAGTAAAATTTTTGTAACTTGTAAAATTTGGTAATAAGATTTCCGCTGGAA

2. Sequences of comp74447, comp74447_c2_seq14 is the assembled sequence with unmapped reads, comp74447_B73_B and comp74447_Oat_B are B-located sequences from B73+B and starter+B, respectively.

>comp74447_c2_seq14

AAGACTCCAATTCCCTTTCAATCTTTGTTGCAGTGAAGAGGTACTTGATCCCTCTACCCAACCATCAGTTAGATAGCTCCATCCTCTTGATGAGCAGACACTGGAGTAGACTCCTCTTCAGAATCTCCTTCTTGGCACTCTTTGCCGAGCATAGAACAAGTAGTACACGACCAAGAATACTTGTGGGAAACCTTGTCCACGGGGAGGTGAAAAACGGTTGGGTTTGGTCACCACAGGCAAGAGGTTGGCATTCATCTCTGGAGTAAAACAAACTTGGTGGCTGAAAGAGAA

>comp74447_B73_B

GATTGAATCTCCTTCTTGGCACTCTACATGAAATGTATGTCCATGAATGCTTATACTCATGTTATGAAAGTGCAACTTGTAAAGAAGACAATATTAAGCACTTCCCACTACACCTTCCCACAAGGTGCACAAAAAGATTAACAAAATAATAGGGGATCTTGTAACACCAAGAATCCAAAGTCTACACCTTCAAACTATGACCACCAGGGGACTAGAACACACTAGATTTCATCCAAAGATCAAGATCTCATTTGATAAACTTGGCTCTACATTCCCAAGCTCTAATCCATTCTTGCATTAGGGATTTATCTTATCTATTATGGACCCTGCAAAGCTTTCTGAGAAAATTGCTTACAAACAGCCTCAAACTTTTACCACAAGCTCAAATGACCAAAACAAGGTTACTTAGAAATTTCCATAAGTTTTGGCACTGTACTTTTATTTCTAAAATCCAAACGGTCCTTCCAAAAGCACTTTTAAATCCCTTAATATTTGGTAACCGACCTAAAAATTCGAAAACCAACTTTACCAGAGAGCTTTTGATTTTATACAGCCACTAAACTTTGTTCCATGATTTTTAGAATTTTCTATAGCACCAGACCAACTTAGTTGGCCAAGCCTGCTCCCAAACCATAGCACTACCAGTTTGCTACAGTAGTCCAAGGTCAAAATCATACAAAACACAAGCTATGGTGCACCAAAACATAACAAAAACACTGAAACATTTCAATTTTAGAAGTCTGAAACAGCACATGTTTAATCTGACAGAGTTTTTCAACTCCTTTTTCTCTACATTCTTTACAGTAGCAAGCCAACTCTCTGTATGACACTTTCTCTCCTTGAAATGGTCTTTCACTTTGCTATACTATAGAATTCTTACCCCAATTCTTCATGGATTAATTACCACAAGGACCCAAATTTGAATAAAAACACACAAATTCAGACTTAAATATTATTGTAAGTCTGAATCCCAACACCTGACAGCCCATATTTTGGCTACTTTTACTCCCAAATTTATGTGGAATCAGGTCCATCTCACTTTAGCAAACTTGTTCACTTTACTATGGTCTACAACTTTGCTATGGAACCCTTAATCCAAAACCCCATTCATCAGTCACAAATGAATTCCAAACTAGGCATACTGCTGCAATTCAGTTCATAGAACTGAATAAAAAATTCAGTTGTGTCCAACCTGACAGTCCAACTTTAACCAACCTTTTCTCCAAGTTTTACATAGCAACAGACCAAGATCACATACTAAAGTTATTCCTTAGATATAGCTCTACTATTTTGGTATAGCACCCCTATGTTGAAAACATTAGAACAATTTTTATGAAGCACCAAAGTGGAGCTCAACACACTAAAACCTAGTTTCAGCCTTATGCACTAAGTCATCCAGAAATCTGACAGCCTATGTTTGAGCAAATTTCACACCTAGTTCCAGGCAACAGACAGTGGTAGAGTACAAGTAGTAAATGGTGTGAGCTTCACTGACCTTTGCCGAGCATAGAACAAGTAGTACACGACCAAGAATACTTGTGGGAAACCTTGTCCACGGGGAGGTGAAAAACGGTTGGGTTTGGTCACCACAGGCAAGAGGTTGGCATTCATCTCTGGAGTAAAACAAACTTGGTGGCAATCACTAGTGAATTC

>comp74447_Oat_B

GATTGAATCTCCTTCTTGGCACTCTACATGAAATGTATGTCCATGAATGCTTATACTCATGTTATGAAAGTGCAACTTGTAAAGAAGACAATATTAAGCACTTCCCACTACACCTTCCCACAAGGTGCACAAAAAGATTAACAAAATAATAGGGGATCTTGTAACACCAAGAATCCAAAGTCTACACCTTCAAACTATGACCACCAGGGGACTAGAACACACTAGATTTCATCCAAAGATCAAGATCTCATTTGATAAACTTGGCTCTACATTCCCAAGCTCTAATCCATTCTTGCATTAGGGATTTATCTTATCTATTATGGACCCTGCAAAGCTTTCTGAGAAAATTGCTTACAAACAGCCTCAAACTTTTACCACAAGCTCAAATGACCAAAACAAGGTTACTTAGAAATTTCCATAAGTTTTGGCACTGTACTTTTATTTCTAAAATCCAAACGGTCCTTCCAAAAGCACTTTTAAATCCCTTAATATTTGGTAACCGACCTAAAAATTCGAAAACCAACTTTACCAGAGAGCTTTTGATTTTATACAGCCACTAAACTTTGTTCCATGATTTTTAGAATTTTCTATAGCACCAGACCAACTTAGTTGGCCAAGCCTGCTCCCAAACCATAGCACTACCAGTTTGCTACAGTAGTCCAAGGTCAAAATCATACAAAACACAAGCTATGGTGCACCAAAACATAACAAAAACACTGAAACATTTCAATTTTAGAAGTCTGAAACAGCACATGTTTAATCTGACAGAGTTTTTCAACTCCTTTTTCTCTACATTCTTTACAGTAGCAAGCCAACTCTCTGTATGACACTTTCTCTCCTTGAAATGGTCTTTCACTTTGCTATACTATAGAATTCTTACCCCAATTCTTCATGGATTAATTACCACAAGGACCCAAATTTGAATAAAAACACACAAATTCAGACTTAAATATTATTGTAAGTCTGAATCCCAACACCTGACAGCCCATATTTTGGCTACTTTTACTCCCAAATTTATGTGGAATCAGGTCCATCTCACTTTAGCAAACTTGTTCACTTTACTATGGTCTACAACTTTGCTATGGAACCCTTAATCCAAAACCCCATTCATCAGTCACAAATGAATTCCAAACTAGGCATACTGCTGCAATTCAGTTCATAGAACTGAATAAAAAATTCAGTTGTGTCCAACCTGACAGTCCAACTTTAACCAACCTTTTCTCCAAGTTTTACATAGCAACAGACCAAGATCACATACTAAAGTTATTCCTTAGATATAGCTCTACTATTTTGGTATAGCACCCCTATGTTGAAAACATTAGAACAATTTTTATGAAGCACCAAAGTGGAGCTCAACACACTAAAACCTAGTTTCAGCCTTATGCACTAAGTCATCCAGAAATCTGACAGCCTATGTTTGAGCAAATTTCACACCTAGTTCCAGGCAACAGACAGTGGTAGAGTACAAGTAGTAAATGGTGTGAGCTTCACTGACCTTTGCCGAGCATAGAACAAGTAGTACACGACCAAGAATACTTGTGGGAAACCTTGTCCACGGGGAGGTGAAAAACGGTTGGGTTTGGTCACCACAGGCAAGAGGTTGGCATTCATCTCTGGAGTAAAACAAACTTGGTGGCAATCACTAGTGAATTC

3. Comp30393 is a 500bp sequence, the B-located sequence has many SNPs compared to its A-genomic counterpart, comp30393_c0_seq1 is the predicted sequence. We got 500bp sequence with comp30393-2F/2R, comp30393_Oat_B and comp30393_B73_B are genomic sequence from Starter+B and B73+B, they have no difference with the B-derived cDNA (comp30393_B73_B_derived_cDNA) but have several SNPs with the A-located sequence of gDNA/cDNA (comp30393_B73_0B).

>comp30393_c0_seq1

CTAATTACTTTTACTGGATTTTCTAACTCTCTGGACAGGGCACATAAATATCAAAAACTACAGGGGCTATCACGCAAAAGATCATAAGACTCAGGATTTCCCCCCGCACTGGATGACGGGTTAATTGTGGCGAAGGGCAGGGGCTCTTAAGTAAGAATGCCAAGCGACGAGGTACGATTTTGCCCTGGCTGCCCGATTAGAGATGGAGGGCCCAGATCACATTGGGACAGCATCGAACCGGTACGCATCACCTATCGTTGGATCAAGATCCAACGGTGCCAACTTTAAACACTAGGGATCCGACCACGGCCGTAAAACAGGGATCGACGGCTCTCGTTTACCCGCGAAGGGGTACGAGCCGGTTTCAATCTCGACCGTATAACCCCCGATCAATGGTCGCCAGTGTCTCTTCCTCCTCCAACCAGCCACCGCGGAGGATGCCAGACGGCGACAGCGCCATTGCTGGCAATTCCCTTCCCCGCGATCTCCCCTCGTGGTTCTCC

AACTGTAACACCCCAGGTTCCACAAACAACCCGGAATGCTACTGAACTACACTTCCGACGTCCATATATAAAATTTCGGCACTTTGCAAGATTTCATAAATGAGTGGGCAACAGGGTATAAACATATATCATGACAAAAACATACTAAGTATTATTAATTGGCTAGCAAGGGAAGATAAACATACGACCTGAACTGAATTAACCAGTGCGCACGATTATATAAAAAAACAAACTTCCTTTGACAATAAGTTTCTTATTAAATCATGGCTGCGCCTGGGCAGCATTATTGTGAGAGTGAAAGTGGGCGTGCTGCTACTCCCCTCATTCCCAACATGCATCAAGTACCGGTGATTGGTGTCGTCCTCAACCAGCGCGACGCAGGGCGGAAACGCAGCGTGCTTCAAGCCCTGATGACAAATCCCACGAGCTCCACCTAGATTCAAGAACGCGCGGCGCGTACCGGTGAACCCTGGTCACCCAGCGAAGTCCCCACCATCGGCACCACGGCTGCAGGGCCGTCCTCTCCTCCTCGCTCAGCTTCAACGCGGTGGCCACAACTTCTCTCCCTCTACTACTCTGCTTGGCTCTCATCTCATGGCGATGGTGGTGCTTTGTGCTTCCTCTGATGATGGACAAGGGTCACGTTGCTTTGGTTTTATACTCCGGAGGAGAGGCTTAGCCTTATCTAGATAGCTTGGTAGTTGTAACGTCCTCGACGAAAGGTTCGGGGCCCTACGATTGCGCAACCAGATTCGGTTCCGTGGGTGAACGACCGCGCTAGGGGACCAACTGGCCAGTGCTAGATCAACATGGGCGCGCACTGCCAGATACCGTGGCGTAGACGAGTTCCAT

>comp30393_Oat_B GGATTTTCTAACTCTCTGGACAGGGCACATAAATATCAAAAACTACAGGGGCTATCACGCAAAAGATCATAAGACTCAGGATTTCCCCCCGCACTGGATGACGGGTTAATTGTGGCGAAGGGCAGGGGCTCTTAAGTAAGAATGCCAAGCGACGAGGTACGATTTTGCCCTGGCTGCCCGATTAGAGATGGAGGGCCCAGATCACATTGGGACAGCATCGAACCGGTACGCATCACCTATCGTTGGATCAAGATCCAACGGTGCCAACTTTAAACACTAGGGATCCGACCACGGCCGTAAAACAGGGATCGACGGCTCTCGTTTACCCGCGAAGGGGTACGAGCCGGTTTCAATCTCGACCGTATAACCCCCGATCAATGGTCGCCAGTGTCTCTTCCTCCTCCAACCAGCCACCGCGGAGGATGCCAGACGGCGACAGCGCCATTGCTGGCAATTCCCTTCCCCGCGATCTCCCCTCGTGGTT

> comp30393_B73_B

GGATTTTCTAACTCTCTGGACAGGGCACATAAATATCAAAAACTACAGGGGCTATCACGCAAAAGATCATAAGACTCAGGATTTCCCCCCGCACTGGATGACGGGTTAATTGTGGCGAAGGGCAGGGGCTCTTAAGTAAGAATGCCAAGCGACGAGGTACGATTTTGCCCTGGCTGCCCGATTAGAGATGGAGGGCCCAGATCACATTGGGACAGCATCGAACCGGTACGCATCACCTATCGTTGGATCAAGATCCAACGGTGCCAACTTTAAACACTAGGGATCCGACCACGGCCGTAAAACAGGGATCGACGGCTCTCGTTTACCCGCGAAGGGGTACGAGCCGGTTTCAATCTCGACCGTATAACCCCCGATCAATGGTCGCCAGTGTCTCTTCCTCCTCCAACCAGCCACCGCGGAGGATGCCAGACGGCGACAGCGCCATTGCTGGCAATTCCCTTCCCCGCGATCTCCCCTCGTGGTT

>comp30393_B73_B_derived_cDNA

GGATTTTCTAACTCTCTGGACAGGGCACATAAATATCAAAAACTACAGGGGCTATCACGCAAAAGATCATAAGACTCAGGATTTCCCCCCGCACTGGATGACGGGTTAATTGTGGCGAAGGGCAGGGGCTCTTAAGTAAGAATGCCAAGCGACGAGGTACGATTTTGCCCTGGCTGCCCGATTAGAGATGGAGGGCCCAGATCACATTGGGACAGCATCGAACCGGTACGCATCACCTATCGTTGGATCAAGATCCAACGGTGCCAACTTTAAACACTAGGGATCCGACCACGGCCGTAAAACAGGGATCGACGGCTCTCGTTTACCCGCGAAGGGGTACGAGCCGGTTTCAATCTCGACCGTATAACCCCCGATCAATGGTCGCCAGTGTCTCTTCCTCCTCCAACCAGCCACCGCGGAGGATGCCAGACGGCGACAGCGCCATTGCTGGCAATTCCCTTCCCCGCGATCTCCCCTCGTGGTT

>comp30393_B73_0B GGATTTTCTAACTCTCTGGACAGGGCGCATTAATATCAAAAACTACAGGGGCTATCATGCAAAAGATCATAAGACTCAGGATTTCCCCCCGCACTGGACGACGGGTTAATTGTGGCGAAGGGTAGGGGCTCTTAAGTAAGAATGCCAAGCGAAGGGGTACGATTAAGACCTGGCCGCCCGATTAGAGATGGAGGGCCCAGATCACATCGGAACAGCATCGAACCGGTACGCGTCACCTATCGTCGGATCAAGATCCAACGGTGCCTACTTTAAACACTAGGGATCCGACCACGGCCGTAAAACACGGGATCGATGGCTCTTGTTTACCCGCGAAGGGGTACGAGACAGTTTCAATCTCGACCGTATAACCCCCGATCAATGGTCACCAGTGTCTCTTCTTCCTCCAACTAGCCACCGCGGCGGATGCCAGATGGCGGCAGCGCCATTGCTGGCAATTCCCTTCCCCGCGATCTCCCCTCGTGGTT
